# Supplementary material for: Triple tandem trimer immunogens for HIV-1 and influenza nucleic acid-based vaccines
Source: NPJ Vaccines. 2024 Apr 6;9:74. doi: 10.1038/s41541-024-00862-8 (PMC10998906; doi:10.1038/s41541-024-00862-8)
Supplement: Supplementary file 1 — Supplementary Material [file 41541_2024_862_MOESM1_ESM.pdf]

Supplementary Material

Triple tandem trimer immunogens for HIV-1 and influenza nucleic acid-based vaccines

del Moral-Sánchez et al.

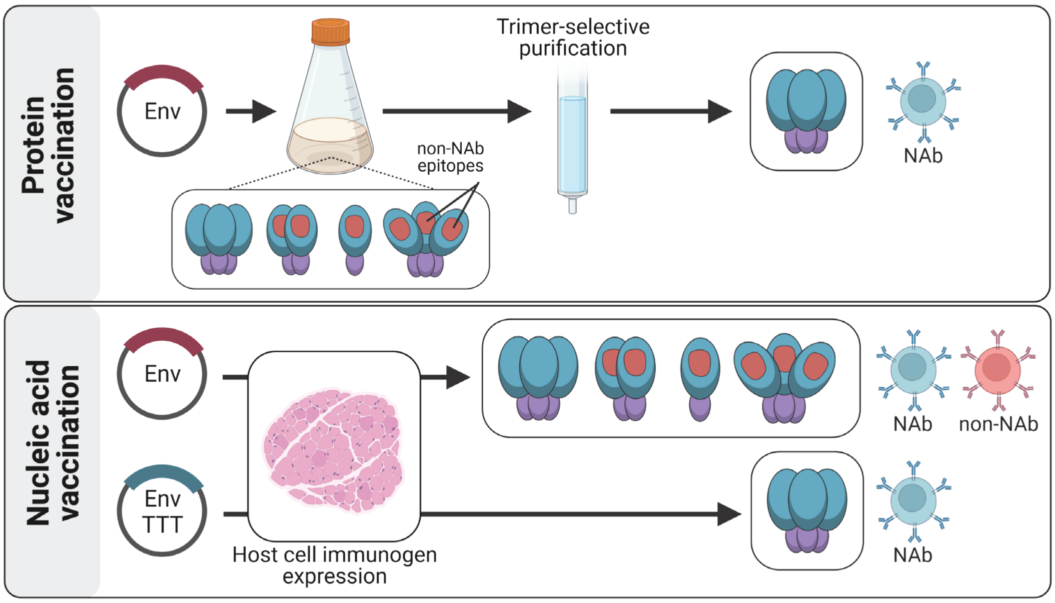

Supplementary Fig. 1 | Value of constructs that express only as trimers for nucleic acid vaccination approaches. Compared to other HIV-1 envelope constructs, such as SOSIP and SC (Env, in purple), triple tandem trimer constructs (Env TTT, in blue) express only as trimers, avoiding the participation of inner non-NAb epitopes in the immune response.

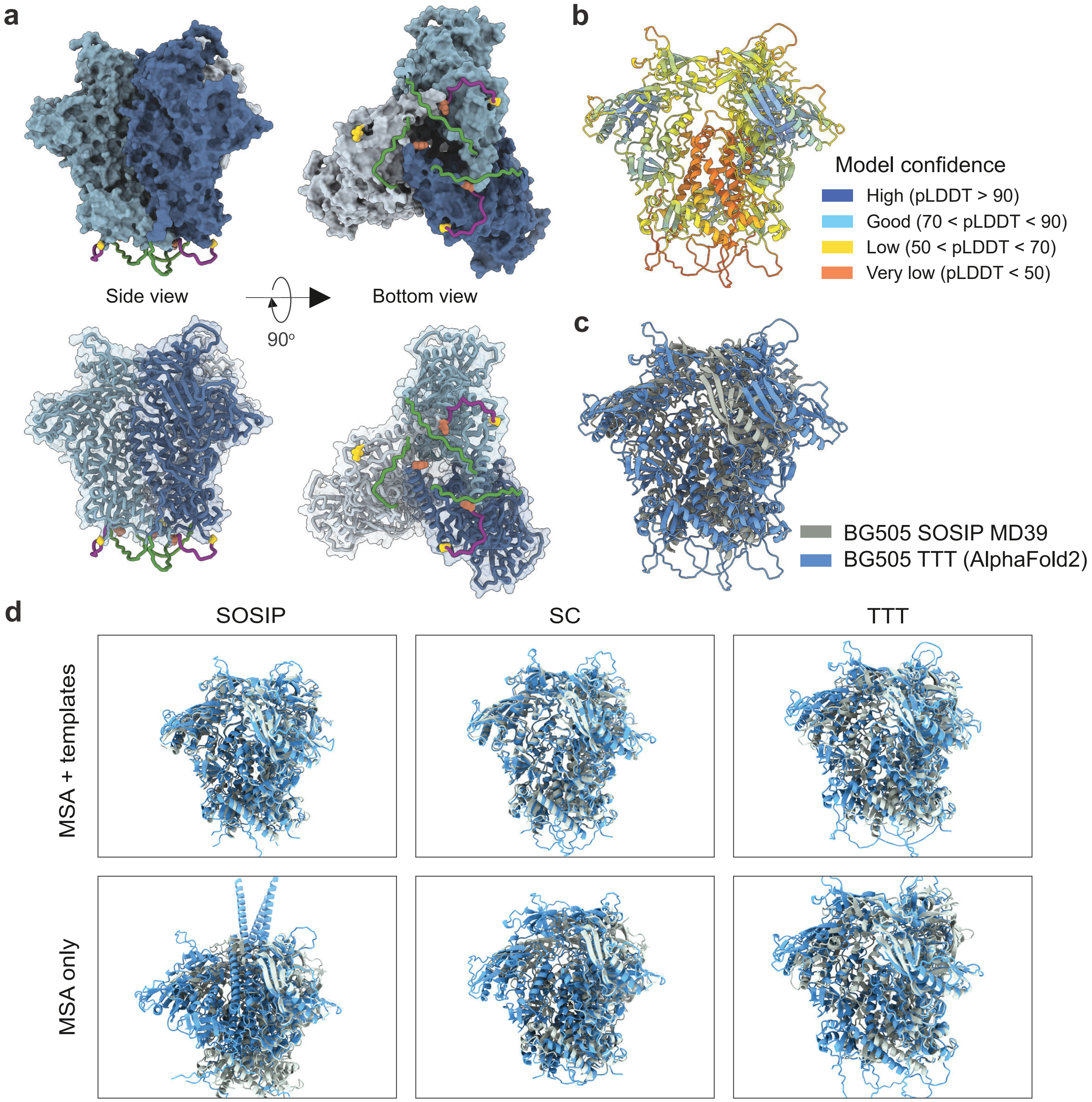

Supplementary Fig. 2 | AlphaFold2-predicted structure of BG505 TTT. a,b Predicted BG505 TTT structure, with SC (green) and TTT (purple) linkers at the base of the trimer **(a)** or colored according to the per-residue estimate of the local confidence of the prediction (pLDDT value) **(b). c** Alignment of the predicted BG505 TTT structure to the experimentally determined structure of a native-like BG505 SOSIP MD39 trimer (PDB 7L8D). **d** AlphaFold2 models of BG505 SOSIP, SC and TTT proteins obtained using multiple sequence alignment (MSA) and PDB templates, or only MSA, as input for the predictions. TTT models were generated using AlphaFol2-monomer, while SOSIP and SC models required AlphaFold2-multimer. All three proteins were predicted to fold with a native-like conformation when both MSA and PDB templates were used. However, when no PDB templates were used, SC and TTT were still predicted to fold with a native-like conformation, but the SOSIP model showed an aberrant post-fusion-like conformation.

a

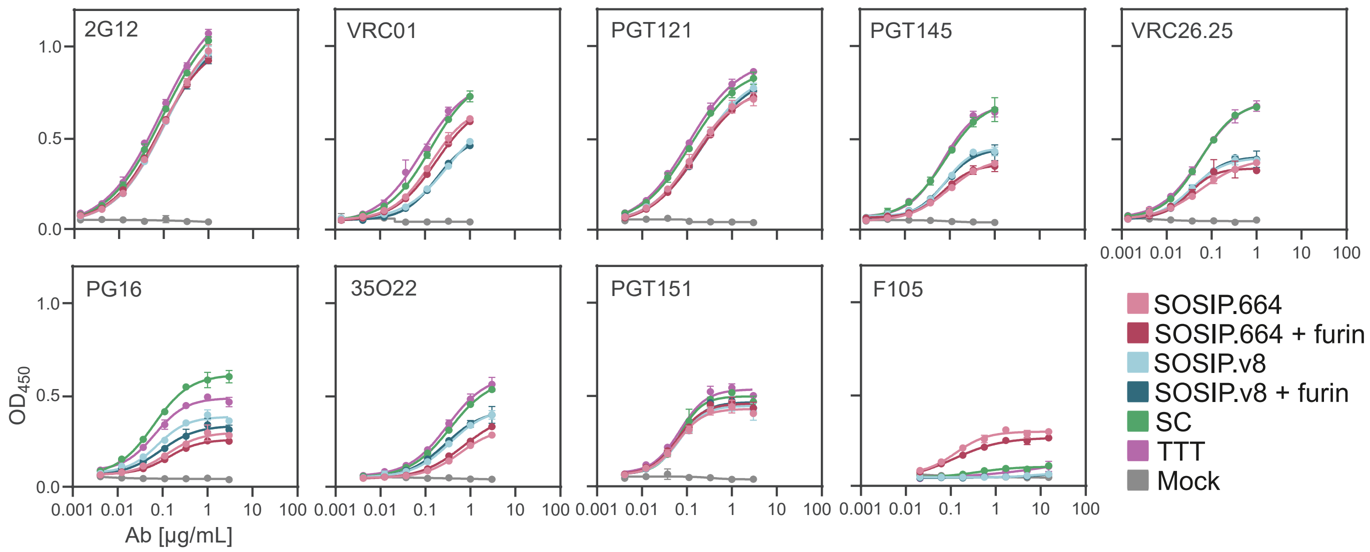

Supplementary Fig. 3 | StrepTactinXT ELISA with supernatants of HEK293T cells transfected with BG505 SOSIP.664, SOSIP.v8, SC and TTT constructs against a panel of bNAbs (2G12, VRC01, PGT121, PGT145, VRC26.25, PG16, 35O22, PGT151) and non-NAbs (F105). Dots and error bars represent the average and standard deviation of values obtained in duplicate.

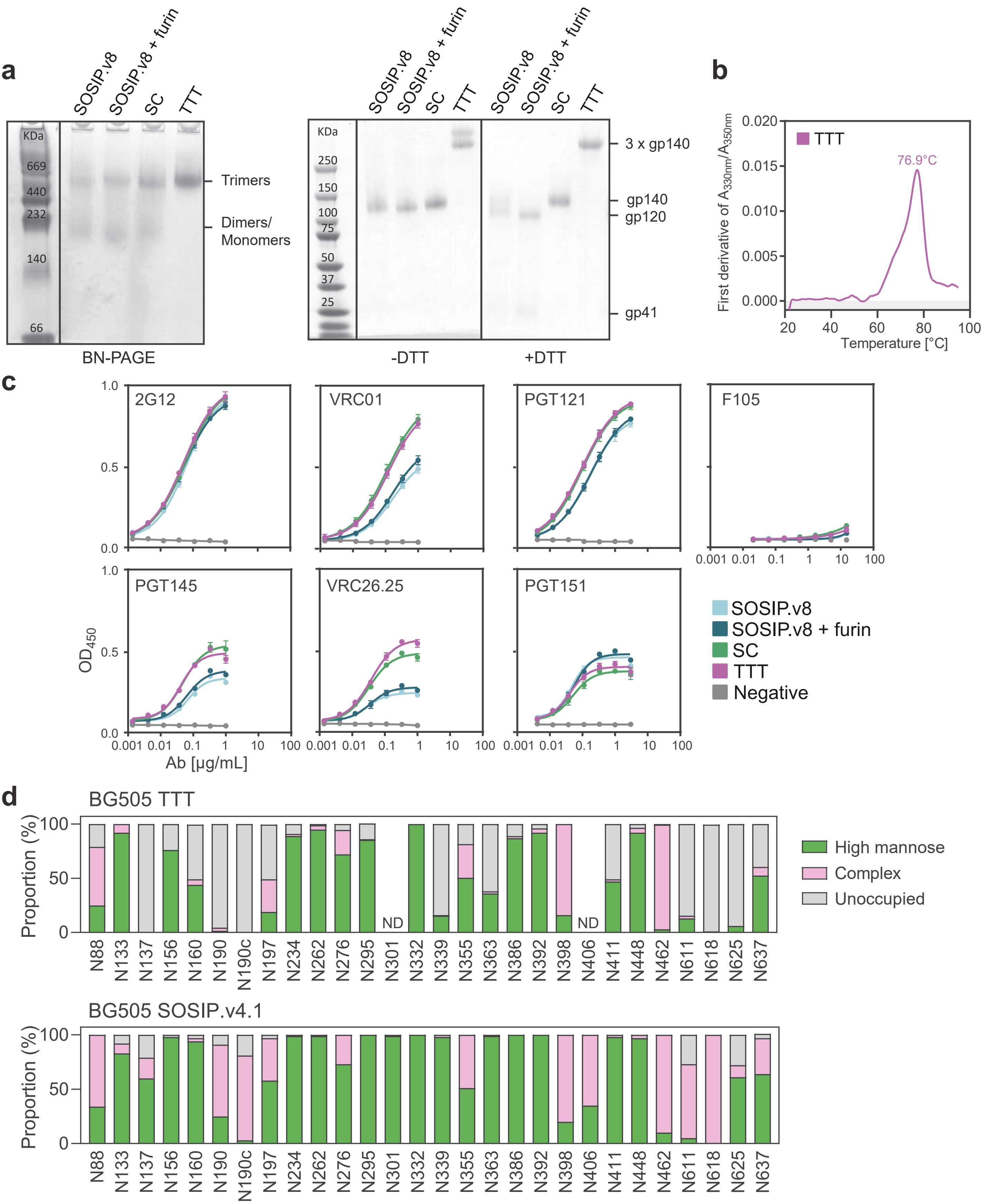

Supplementary Fig. 4 | Biophysical characterization of BG505 TTT purified protein. a BN-PAGE, non-reducing (-DTT) and reducing (+DTT) SDS-PAGE analysis of StrepTactinXT-purified SOSIP.v8, SC and TTT protein preparations. The single band above 250 kDa in reducing SDS-PAGE confirmed that BG505 TTT forms covalently linked trimers. **b** Denaturing profile of GNL-purified TTT protein, obtained by nanoDSF and used to determine the T_m value presented on **Table 1. c** StrepTactinXT ELISA with StrepTactinXT-purified SOSIP.v8, SC and TTT protein preparations against a panel of bNAbs (2G12, VRC01, PGT121, PGT145, VRC26.25, PGT151) and non-NAbs (F105). Dots and error bars represent the average and standard deviation of values obtained in duplicate. **d** Site-specific glycan analysis of GNL-purified BG505 TTT. Values represented are specified in **Supplementary Table 2.** Site-specific glycan analysis of 2G12-purified BG505 SOSIP.v4.1 protein, from a previous **(continues on next page)**

(continued from previous page) study[1], is presented for comparison. PNGS are displayed as aligned with HxB2. Data could not be determined (ND) for sites N301 and N406. The glycan modifications on the remaining sites were classified into three categories: high mannose (corresponding to any composition containing two HexNAc residues, or three HexNAc and at least 5 hexoses), complex, or unoccupied. The proportion of peptides and glycopeptides corresponding to each of these categories was colored green for high mannose, pink for complex and grey for unoccupied. It is important to note that, as the amino acid sequence for all subunits is identical, this data represents an average of the glycosylation across all three protomers that comprise the trimer, and it is not possible to assign particular glycan occupancy and processing states to a particular protomer in the trimer. This analysis revealed that the processing of BG505 TTT was comparable with that of SOSIP trimers, and that key glycan bnAb epitopes are conserved across both formats. For instance, the key N332 glycan supersite and the N262 site, which has been shown to stabilize the structure of Env, contained almost exclusively oligomannose-type glycans. Furthermore, sites that are occupied by fully processed, complex-type glycans are conserved across both platforms, such as at N398 and N462. Additionally, several sites displayed prominent levels of unoccupied asparagines, most notably on gp41 and in the V1V2 region. These sites are similarly underoccupied on SOSIP trimers[1]. In addition to these sites, the BG505 TTT displayed reduced occupancy at N160, which might be caused by the fact that the TTT was not purified by PGT145, which recognizes oligomannose-type glycans at this site. Additionally, N339, N363 and N411 were more unoccupied on BG505 TTT compared to BG505 SOSIP.

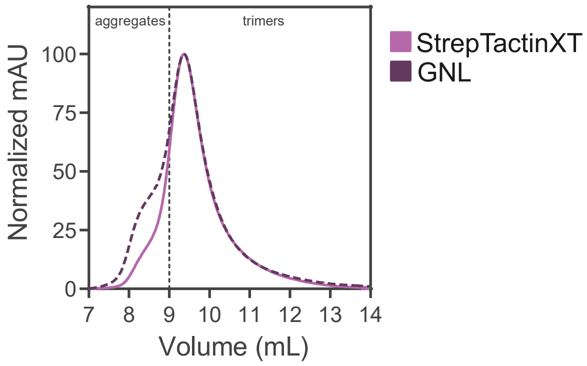

Supplementary Fig. 5 | SEC profiles of StrepTactinXT- and GNL-purified BG505 TTT protein preparations expressed in HEK293F cells on a Superdex 200 Increase 10/300 GL column.

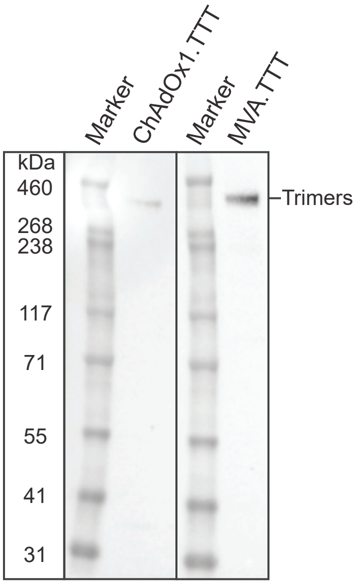

Supplementary Fig. 6 | Expression of ChAdOx1- and MVA-vectored BG505 TTT in HeLa cells. TTT expression was confirmed by Western blot analysis of HeLA cell-infected lysate, using an anti-HIV-1 Env monoclonal (ARP3119) primary antibody and a peroxidase-conjugated goat anti-mouse secondary antibody followed by chemiluminescence.

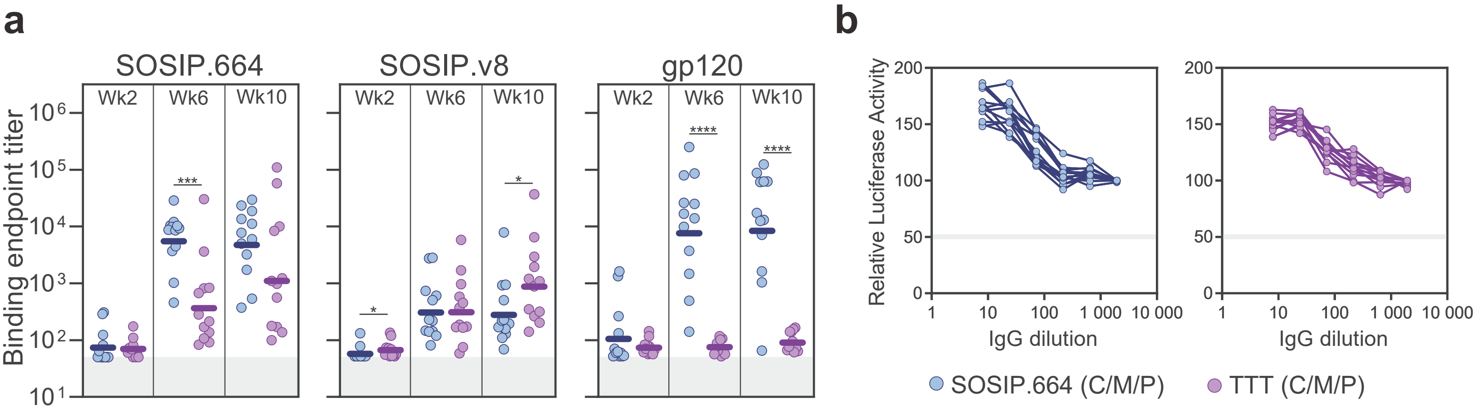

Supplementary Fig. 7 | Immunogenicity of viral-vectored TTT in mice. a Endpoint antibody-binding titers of mice sera over time against SOSIP.664, SOSIP.v8 and gp120 proteins, as measured by D7324-capture ELISA. Median values are represented by horizontal lines. Statistical differences between groups were determined using a two-tailed Mann-Whitney U test (*p < 0.05, **p < 0.01, ***p < 0.001, ****p < 0.0001). Individual values are detailed in **Supplementary Table 4. b** Neutralization of an autologous BG505/T332N pseudovirus by IgGs purified from week 10 mice sera. Dots represent mean values of replicate samples, determined by a TZM-bl assay.

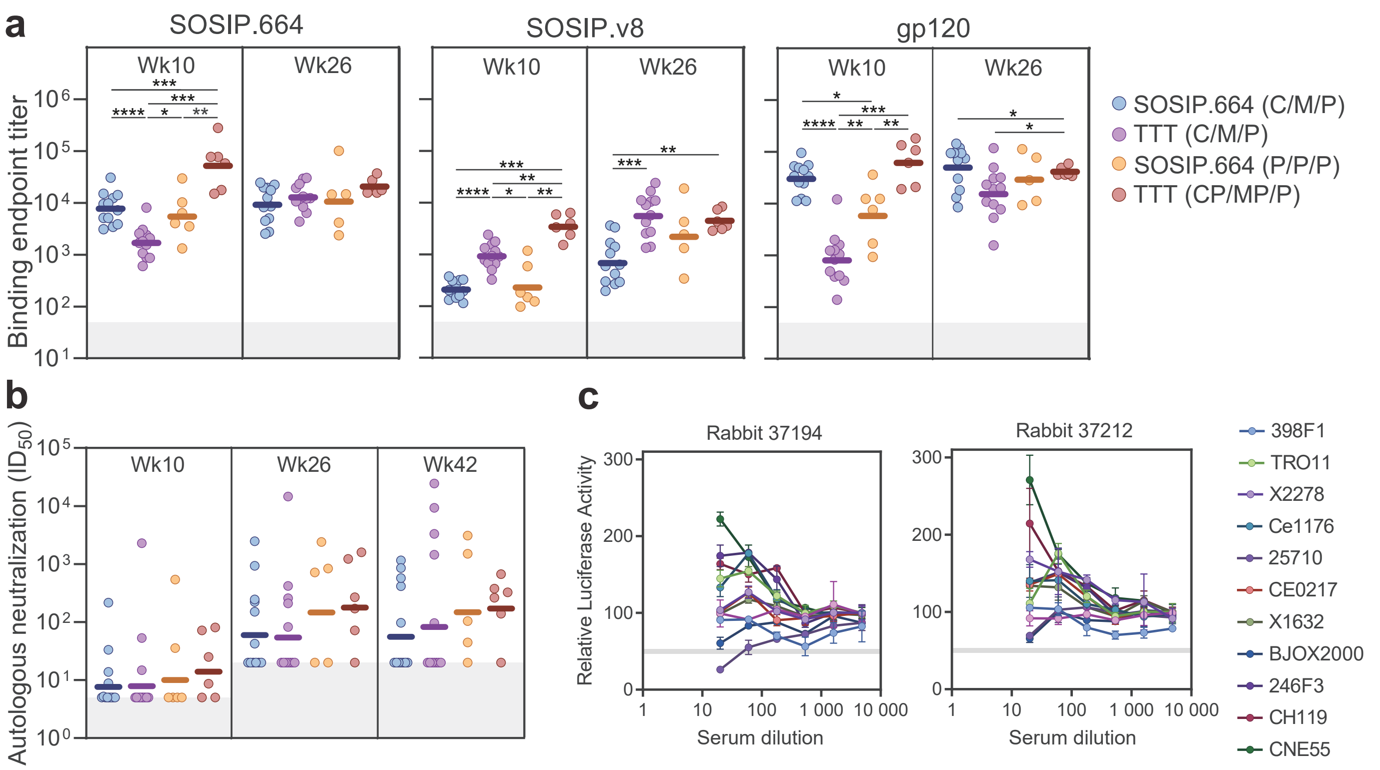

Supplementary Fig. 8 | Immunogenicity of viral-vectored TTT in rabbits. a Endpoint antibody binding titers at weeks 10 and 26 against SOSIP.664, SOSIP.v8 and gp120 proteins, as measured by D7324-capture ELISA. **b** Midpoint neutralization titers (ID_{50}) at weeks 10, 26 and 42 for sera of the immunized rabbits against a BG505/T332N autologous pseudovirus. **c** Neutralization of a panel of heterologous pseudoviruses by the week 42 sera of rabbits 37194 and 34212. Dots represent mean values of replicate samples determined by a TZM-bl assay. Nine of the viruses used are part of the global panel of HIV-1 Env reference strains[2]. Horizontal lines **(a,b)** represent median **(a)** and geometric mean **(b)** values. Individual values **(a,b)** are detailed in **Supplementary Tables 5,6.** Statistical differences between groups **(a,b)**, determined by a two-tailed Mann-Whitney U test, are represented by asterisks (*p < 0.05, **p < 0.01, ***p < 0.001, ****p < 0.0001).

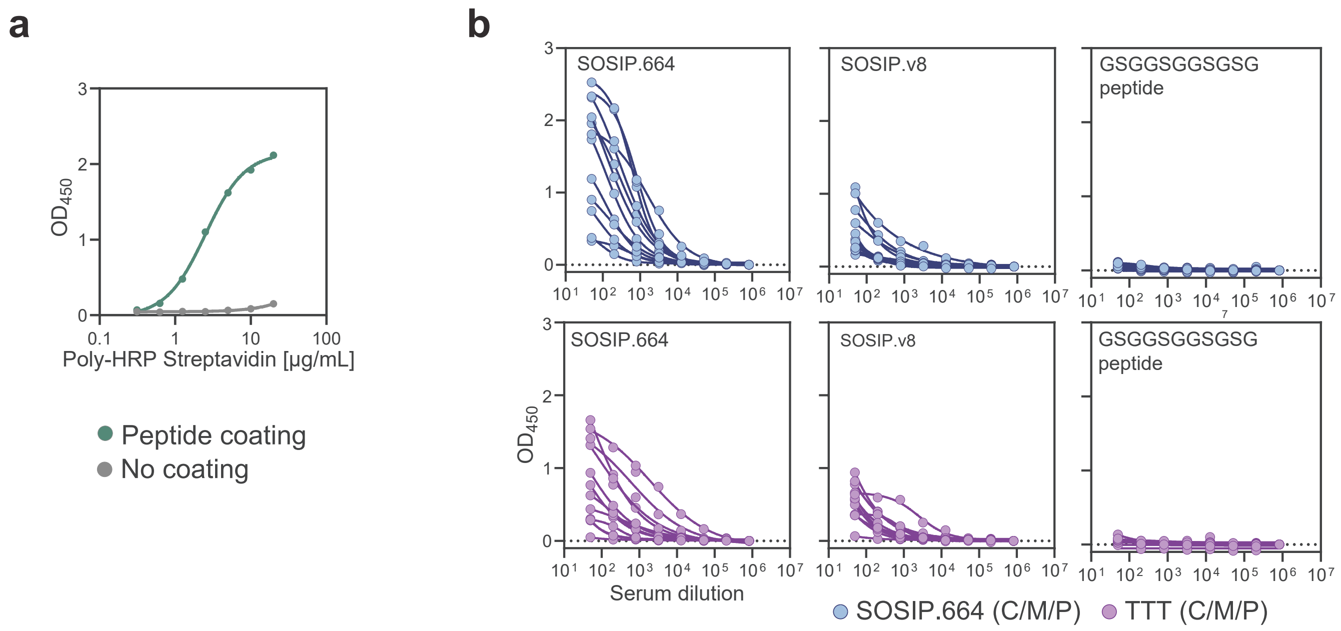

Supplementary Fig. 9 | Immunogenicity of the TTT linkers. a TTT linker-like GSGGSGGSGSG peptide directly immobilized on a half-area 96-well ELISA plate. The peptide is N-terminally biotinylated, which allows detection using Poly-HRP streptavidin. **b** Binding of week 10 mice sera to the GSGGSGGSGSG peptide and to D7324-captured SOSIP.664 and SOSIP.v8 proteins.

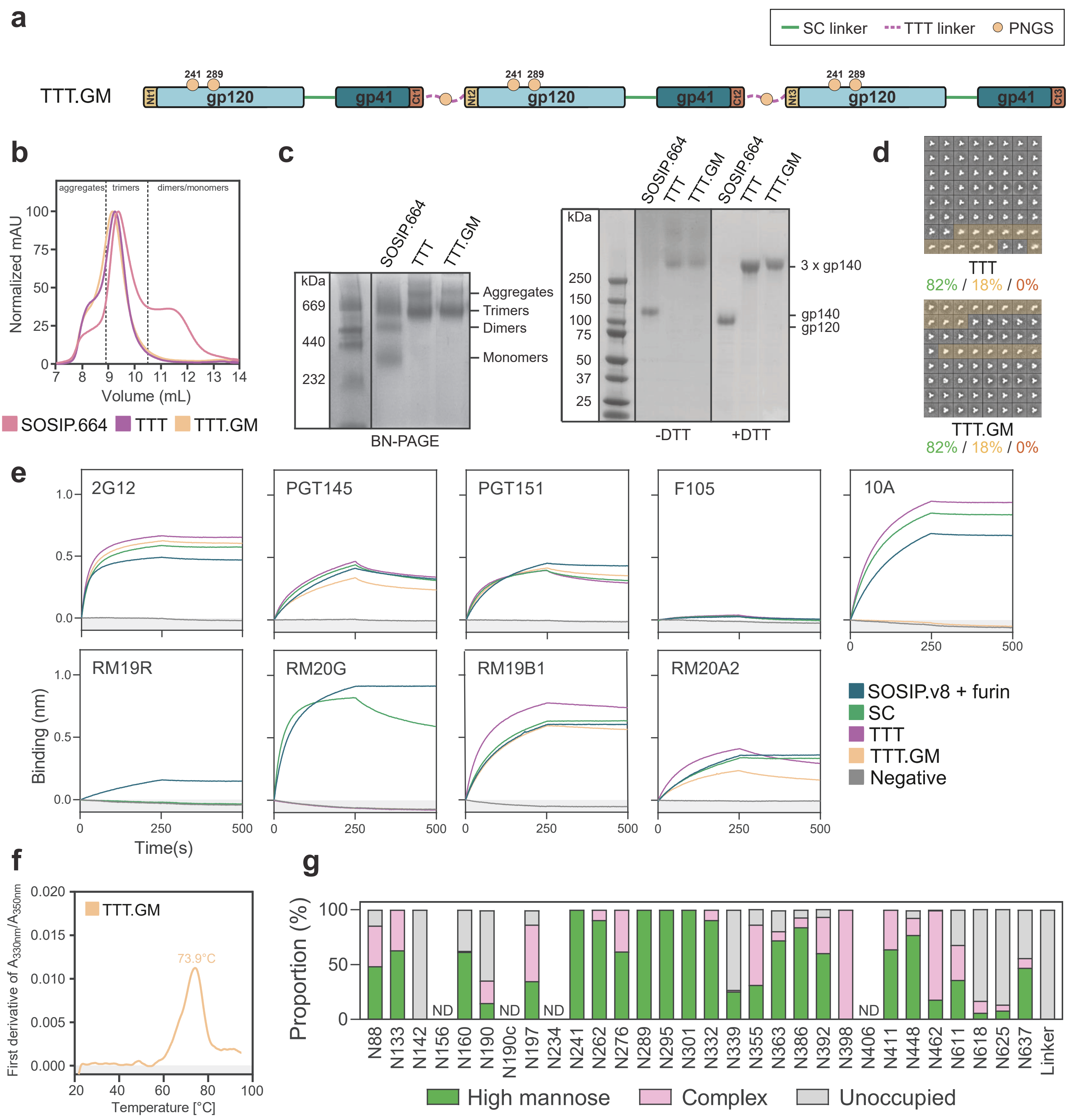

Supplementary Fig. 10 | Design and biophysical characterization of BG505 TTT.GM construct. a Linear representation of the BG505 TTT.GM construct, derived from BG505 TTT **(Fig. 1a)** by including PNGS motifs at positions 241 and 289 and on the TTT linkers. **b** SEC profile of GNL-purified BG505 SOSIP.664, TTT and TTT.GM protein preparations on a Superdex 200 Increase 10/300 GL column. **c** BN-PAGE, non-reducing (-DTT) and reducing (+DTT) SDS-PAGE analyses of GNL-purified SOSIP.664, TTT and TTT.GM protein preparations. **d** nsEM-generated 2D class averages generated by nsEM analysis of the GNL-purified TTT and TTT.GM proteins. Percentages of native-like trimers (green), malformed trimers (yellow) and monomers/dimers (red) are indicated. 2D class averages representing non-native-like particles are shaded in the corresponding yellow or red colors. **e** ProtA BLI assay with GNL-purified SOSIP, SC, TTT and TTT.GM protein preparations against a panel of bNAbs (2G12, PGT145, PGT151), non-NAbs (F105), 241/289 hole-targeting (10A) and base hole-targeting (RM19R, RM20G, RM19B1 and RM20A2) antibodies. **f** Denaturing profile of GNL-purified TTT.GM protein, obtained by nanoDSF and used to determine the T_m value presented on **Table 1. g** Site-specific glycan analysis of GNL-purified TTT.GM protein. Values are specified in **Supplementary Table 7.** PNGS are displayed as aligned with HxB2. Data could not be determined (ND) for sites N156, N190c, N234 and N406. **(continues on next page)**

(continued from previous page) The glycan modifications on the remaining sites were classified into three categories: high mannose (corresponding to any composition containing two HexNAc residues, or three HexNAc and at least 5 hexoses), complex, or unoccupied. The proportion of peptides and glycopeptides corresponding to each of these categories was colored green for high mannose, pink for complex and grey for unoccupied. It is important to note that as the amino acid sequence for all subunits is identical, this data represents an average of the glycosylation across all three protomers that comprise the trimer, and it is not possible to assign particular glycan occupancy and processing states to a particular protomer in the trimer. As expected, the newly incorporated N241 and N289 sites were efficiently populated by oligomannose-type glycans, which explained the 10A binding abrogation. Furthermore, the insertion of these sites appeared to subtly influence the processing of sites around the 241 and 289 positions and increased the abundance of oligomannose-type glycans at N611, as previously reported[3]. The PNGS incorporated in the TTT linkers were, however, largely unoccupied.

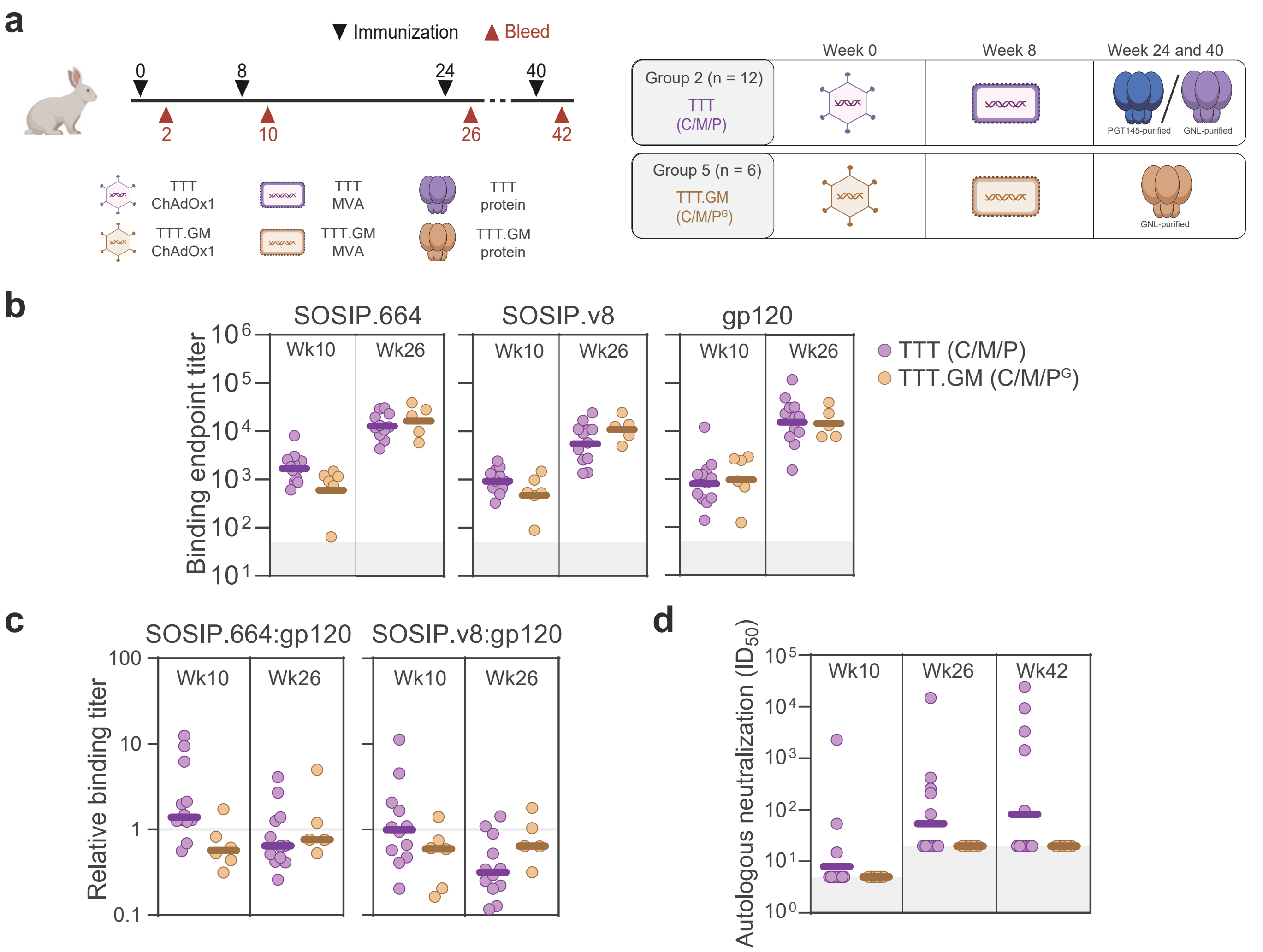

Supplementary Fig. 11 | Immunogenicity of viral-vectored TTT.GM in rabbits. a Immunization schedule of rabbits vaccinated with TTT.GM. A group of six NZW rabbits were vaccinated (black arrows) at weeks 0, 8, 24 and 40 with the immunogens indicated on the table. Antibody responses were evaluated (red arrows) at weeks 10, 26 and 42. **b** Endpoint antibody binding titers at weeks 10 and 26 against SOSIP.664, SOSIP.v8 and gp120 proteins, as measured by D7324-capture ELISA. **c** Ratios between trimer (SOSIP.664 or SOSIP.v8)-binding and gp120-binding titers **d** Midpoint neutralization titers (ID_{50}) at weeks 10, 26 and 42 for sera of the immunized rabbits against a BG505/T332N autologous pseudovirus. Horizontal lines represent median **(b,c)** and geometric mean **(d)** values. Individual values **(b,d)** are detailed in **Supplementary Tables 5,6.** No statistical differences between groups **(b-d)** were found, as estimated by a two-tailed Mann-Whitney U test.

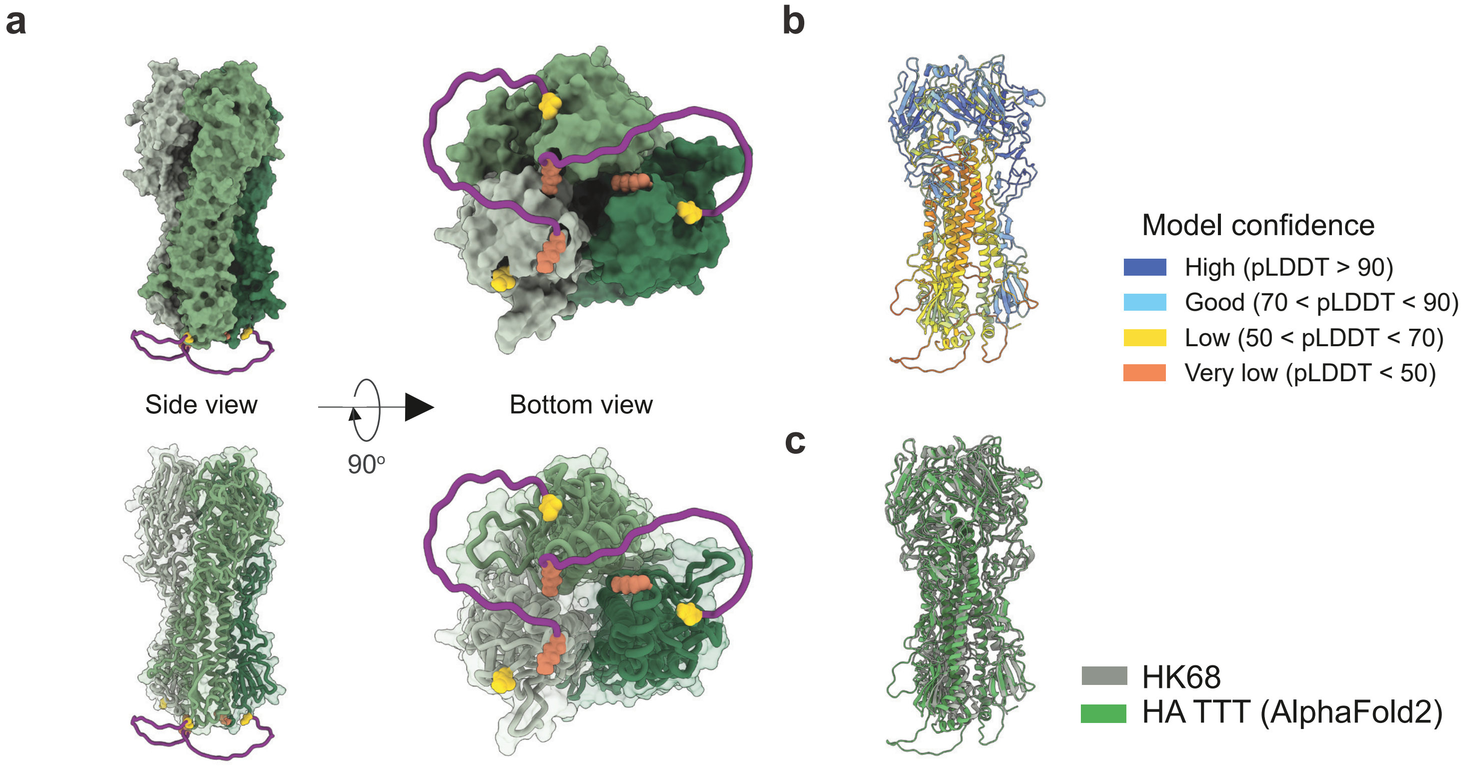

Supplementary Fig. 12 | AlphaFold2-predicted structure of H3 HA TTT. a,b Predicted HA TTT structure, with TTT (purple) linkers at the base of the trimer **(a)** or colored according to the per-residue estimate of the local confidence of the prediction (pLDDT value) **(b). c** Alignment of the predicted HA TTT structure to the experimentally determined structure of a native-like HK68 (A/Hong Kong/1/1968(H3N2), PDB 6NHP) HA trimer.

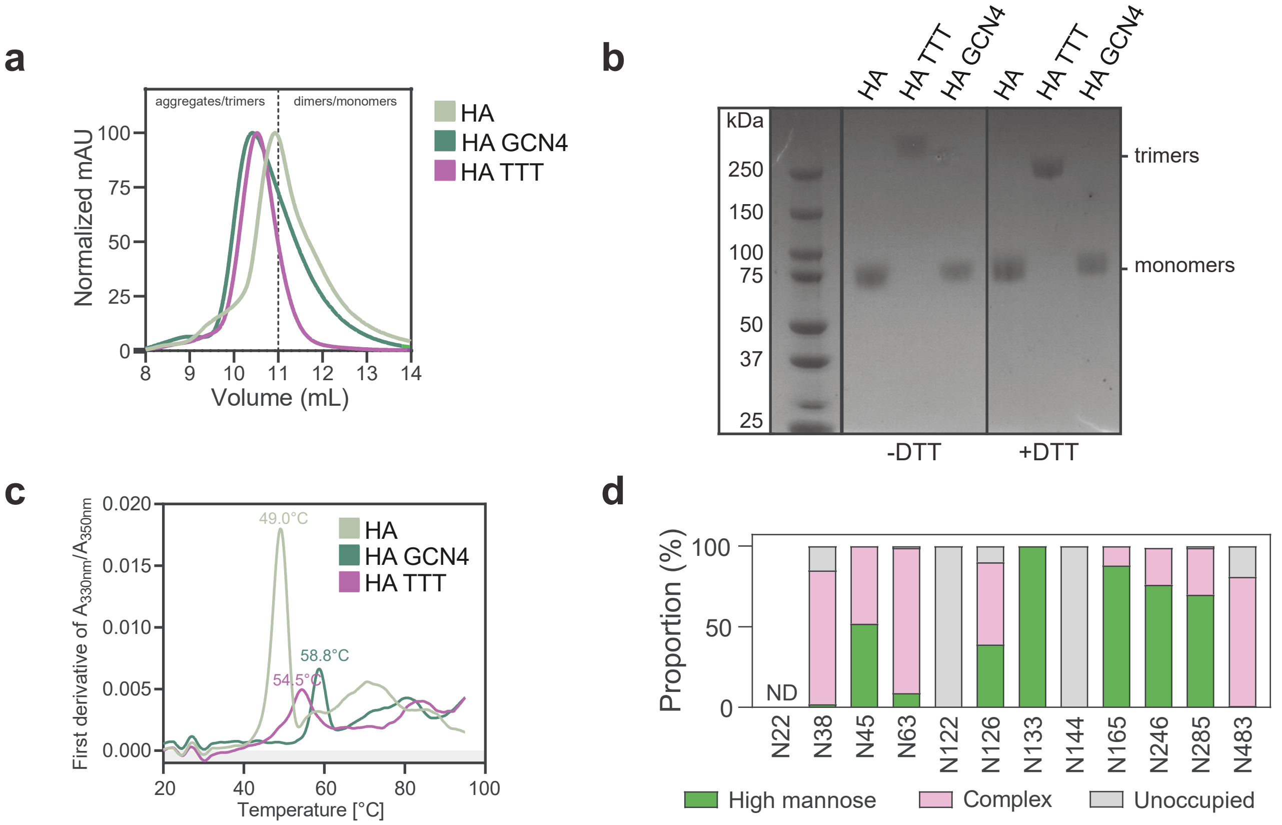

Supplementary Fig. 13 | Biophysical characterization of the H3 HA TTT protein. a SEC profiles of StrepTactinXT-purified H3 HA, HA GCN4 and HA TTT protein preparations expressed in HEK293F cells on a Superdex 200 Increase 10/300 GL column. **b** Non-reducing (-DTT) and reducing (+DTT) SDS-PAGE analyses of StrepTactinXT-purified HA, HA GCN4 and HA TTT proteins. The single band above 250 kDa in reducing SDS-PAGE confirmed that HA TTT forms covalently linked trimers. **c** Denaturing profiles of the purified HA, HA GCN4 and HA TTT proteins, obtained by nanoDSF and used to determinate the T_m values presented on **Table 1. d** Site-specific glycan analysis of purified HA TTT. Values are specified in **Supplementary Table 8.** PNGS are displayed according to H3 numbering. Data could not be determined (ND) for site N22. The glycan modifications on the remaining sites were classified into three categories: high mannose (corresponding to any composition containing two HexNAc residues, or three HexNAc and at least 5 hexoses), complex, or unoccupied. The proportion of peptides and glycopeptides corresponding to each of these categories was colored green for high mannose, pink for complex and grey for unoccupied. It is important to note that as the amino acid sequence for all subunits is identical, this data represents an average of the glycosylation across all three protomers that comprise the trimer, and it is not possible to assign particular glycan occupancy and processing states to a particular protomer in the trimer. This site-specific glycan analysis revealed diverse distributions of oligomannose- and complex-type glycans at different sites. Underprocessed oligomannose glycans were dominant at N133, N165, N246 and N285, while N38, N63 and N483 were occupied mostly with complex glycans. N45 and N126 showed high abundance of both oligomannose and complex glycans. Overall, the glycosylation of the HA TTT corresponds well with that of H3 HA as analyzed previously[4,5]. Both these studies revealed high levels of underprocessed oligomannose-type glycans at N133, N165, N246 and N285, while mixtures or complex glycans were present at other sites. Sites N122 and N144 were not occupied by a glycan on HA TTT, but this is also consistent with previous observations using trimeric HA protein[5], implying that the underoccupancy at these sites is not a result of the TTT format, but rather a consequence of the surrounding primary sequence and/or structure.

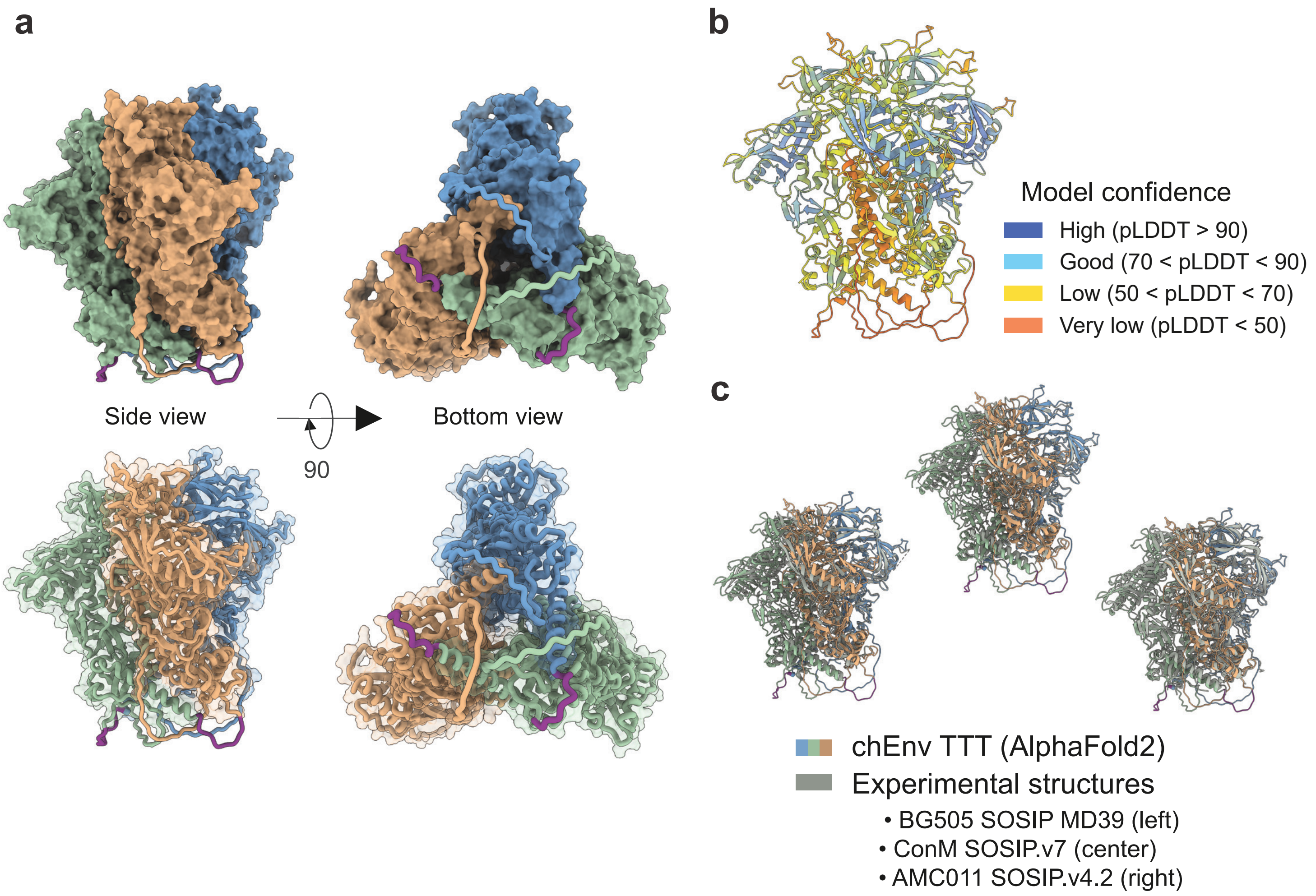

Supplementary Fig. 14 | AlphaFold2-predicted structure of chEnv TTT. a Predicted chEnv TTT structure, with SC (green) and TTT (purple) linkers at the base of the trimer. BG505, ConM and AMC011 protomers are colored in blue, green and orange, respectively. **b** Predicted structure colored according to the per-residue estimate of the local confidence of the prediction (pLDDT value). **c** Alignment of the predicted chEnv TTT structure to the experimentally determined structures of native-like BG505 SOSIP MD39 (PDB 7L8D), ConM SOSIP.v7 (PDB 6IEQ) and AMC011 SOSIP.v4.2 (PDB 6NC3).

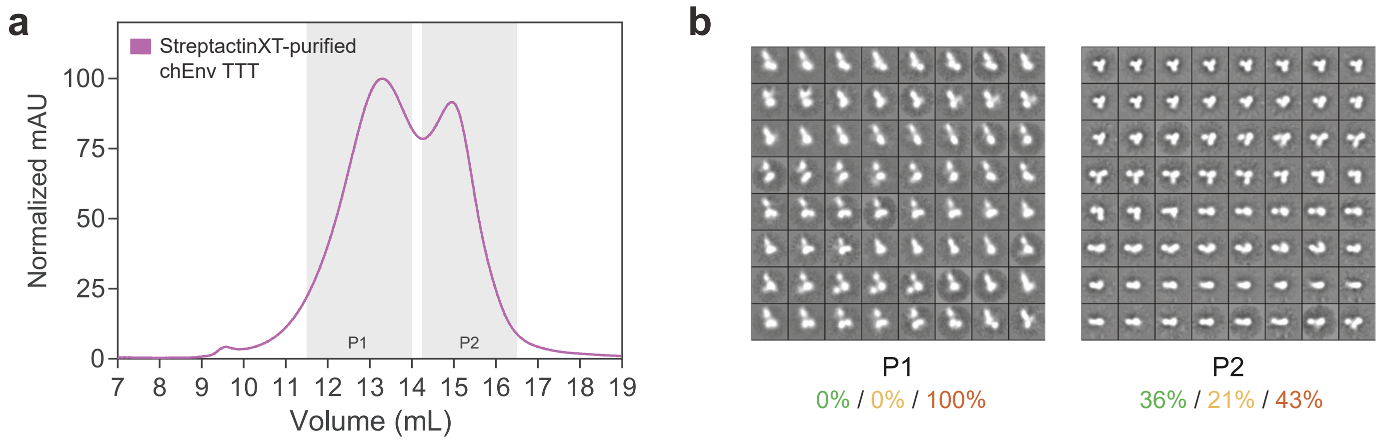

Supplementary Fig. 15 | a SEC profile of the StreptactinXT-purified chEnv TTT protein expressed in HEK293F cells on a Superose 6 Increase 10/300 GL column. **b** nsEM-generated 2D class averages of fraction pools (P1 and P2) corresponding to each of the peaks in the SEC profile, with the percentages of native-like trimers (green), malformed trimers (yellow) and monomers/dimers (red) indicated.

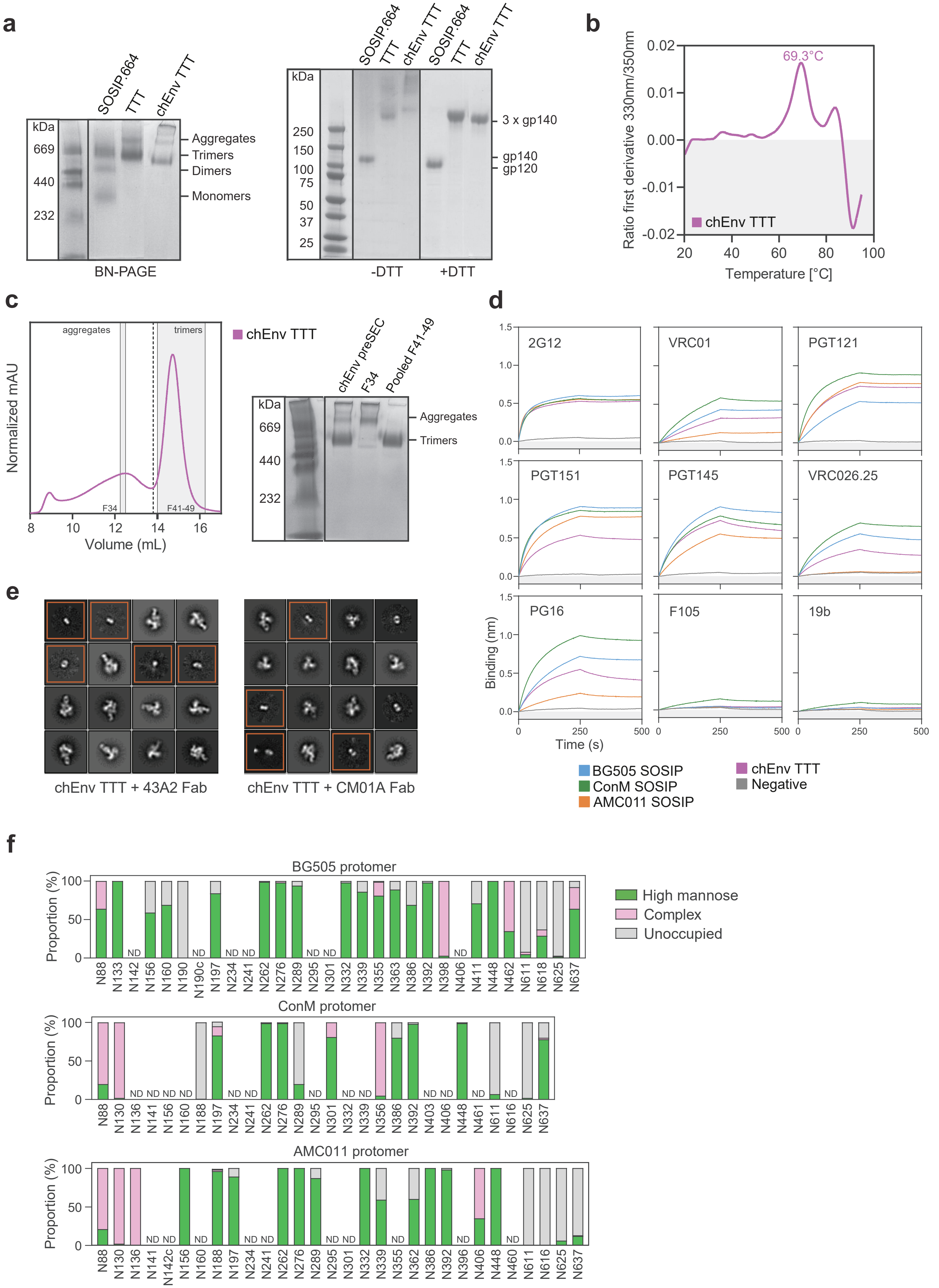
a
SOSIP.664
TTT
chEnv TTT
kDa
669
440
232
Aggregates
Trimers
Dimers
Monomers
BN-PAGE
250
150
100
75
50
37
25
3 x gp140
gp140
gp120
-DTT
+DTT
b
0.02
0.01
0.00
-0.01
-0.02
Ratio first derivative 330nm/350nm
69.3°C
chEnv TTT
20
40
60
80
100
Temperature [°C]
c
aggregates
trimers
F34
F41-49
Normalized mAU
8
10
12
14
16
Volume (mL)
chEnv TTT
chEnv preSEC
F34
Pooled F41-49
kDa
669
440
232
Aggregates
Trimers
d
2G12
VRC01
PGT121
PGT151
PGT145
VRC026.25
PG16
F105
19b
1.5
1.0
0.5
0.0
Binding (nm)
0
250
500
Time (s)
BG505 SOSIP
ConM SOSIP
AMC011 SOSIP
chEnv TTT
Negative
e
chEnv TTT + 43A2 Fab
chEnv TTT + CM01A Fab
f
BG505 protomer
Proportion (%)
100
50
0
N88 N133 N142 N156 N160 N190 N190c N197 N234 N241 N262 N276 N289 N295 N301 N332 N339 N355 N363 N386 N392 N398 N406 N411 N448 N462 N611 N618 N625 N637
ND
High mannose
Complex
Unoccupied
ConM protomer
N88 N130 N136 N141 N156 N160 N188 N197 N234 N241 N262 N276 N289 N295 N301 N332 N339 N356 N386 N392 N403 N406 N448 N461 N611 N616 N625 N637
AMC011 protomer
N88 N130 N136 N141 N142c N156 N160 N188 N197 N234 N241 N262 N276 N289 N295 N301 N332 N339 N355 N362 N386 N392 N396 N406 N448 N460 N611 N616 N625 N637

Supplementary Fig. 16 | Biophysical characterization of the chEnv TTT protein. a BN-PAGE, non-reducing (-DTT) and reducing (+DTT) SDS-PAGE analyses of PGT145-purified chEnv TTT protein. GNL-purified SOSIP.664 and BG505 TTT were included as controls. The single band above 250 kDa in reducing SDS-PAGE confirmed that chEnv TTT forms covalently linked trimers, similarly to BG505 TTT. **b** Denaturing profile of the PGT145-purified chEnv TTT protein, obtained by nanoDSF and used to determine the T_m value presented on **Table 1. c** SEC profile of the PT145-purified chEnv TTT protein expressed in HEK293F cells on a Superose 6 Increase 10/300 GL column (left), showing substantial aggregation, and BN-PAGE (right) of the input protein and specific (pools of) fractions. **d** ProtA BLI assay with PGT145+SEC-purified chEnv TTT protein against a panel of bNAbs (2G12, VRC01, PGT121, PGT151, PGT145, VRC026.25, PG16) and non-NAbs (F105, 19b). PGT145-purified BG505 SOSIP.v5.2, ConM SOSIP.v7 and AMC011 SOSIP.v9 were included as homotrimeric controls. The experiment was performed in duplicate, and the curves shown correspond to one of these repetitions. **e** Representative 2D class averages of chEnv TTT protein in complex with 43A2 and CM01A Fabs, sorted in reverse by number of particles in each class. Classes with unbound Fab are marked in red. **f** Site-specific glycan analysis of PGT145-purified chEnv TTT protein. Values are specified in **Supplementary Table 9.** PNGS are displayed as aligned with HxB2. Data could not be determined (ND) for several sites. The glycan modifications on the remaining sites were classified into three categories: high mannose (corresponding to any composition containing two HexNAc residues, or three HexNAc and at least 5 hexoses), complex, or unoccupied. The proportion of peptides and glycopeptides corresponding to each of these categories was colored green for high mannose, pink for complex and grey for unoccupied. This analysis revealed a glycosylation profile similar to the one of BG505 TTT **(Supplementary Fig. 4d),** with the N332 glycan supersite populated almost exclusively by oligomannose-type glycans and gp41 asparagines prominently unoccupied. Sites N88, N130 and N136 in the N-termini of the second (ConM) and third (AMC011) protomers contained a high proportion of complex-type glycans. Remarkably, this high proportion of complex-type glycans in site N130 has been previously observed in a membrane-bound unmodified AMC011 trimer, but not in a soluble SOSIP counterpart[6].

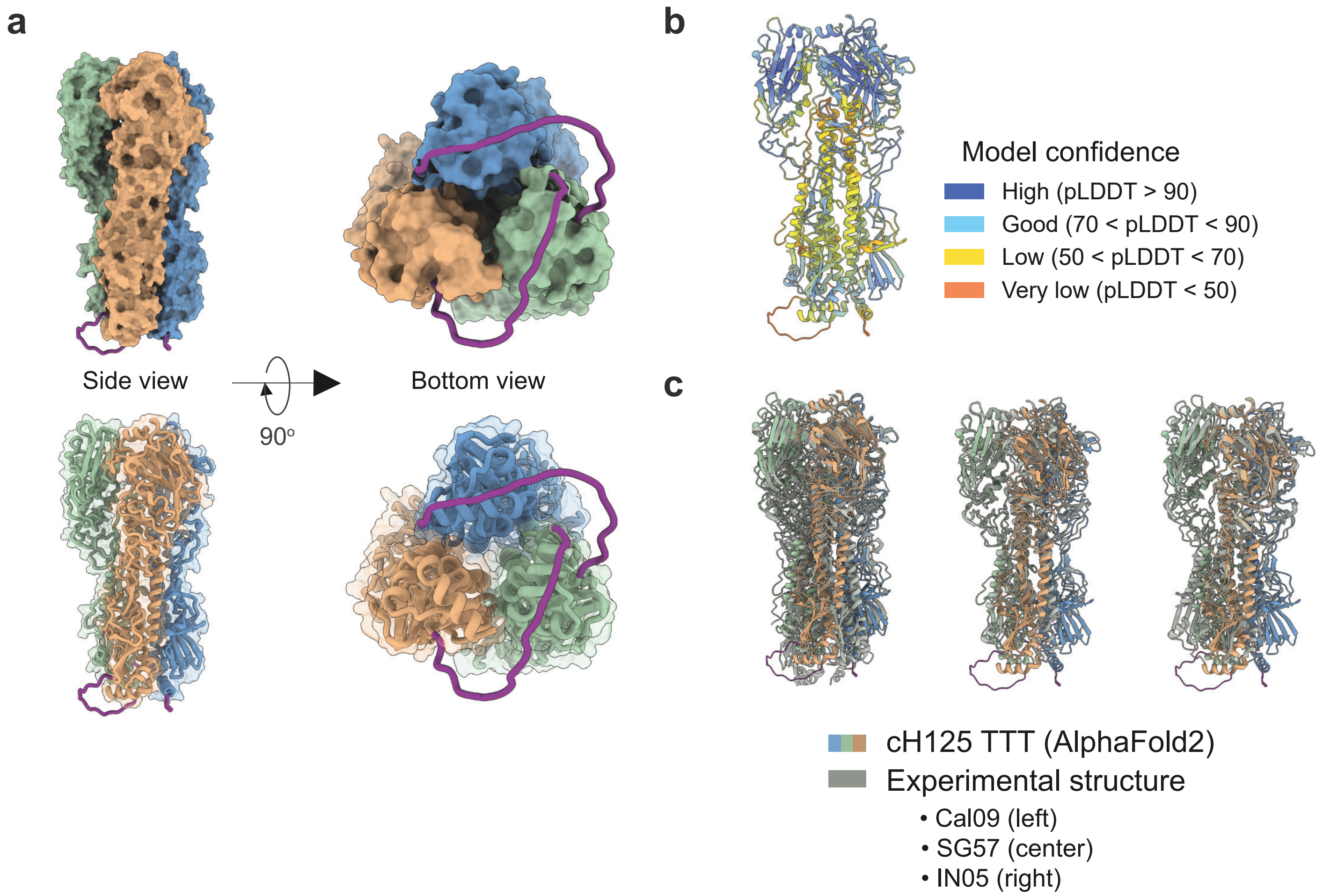

Supplementary Fig. 17 | AlphaFold2-predicted structure of cH125 TTT. a Predicted cH125 TTT structure, with TTT (purple) linkers at the base of the trimer and NL09 (A/Netherlands/602/2009(H1N1)), SG57 (A/Singapore/1/1957(H2N2)) and IN05 (A/Indonesia/5/2005(H5N1)) protomers colored in blue, green and orange, respectively. **b** Predicted structure colored according to the per-residue estimate of the local confidence of the prediction (pLDDT value). **c** Alignment of the predicted cH125 TTT structure to the experimentally determined structures of Cal09 (A/California/04/2009(H1N1), PDB 3LZG), SG57 (PDB 2WR7) and IN05 (PDB 4K62) native-like trimers.

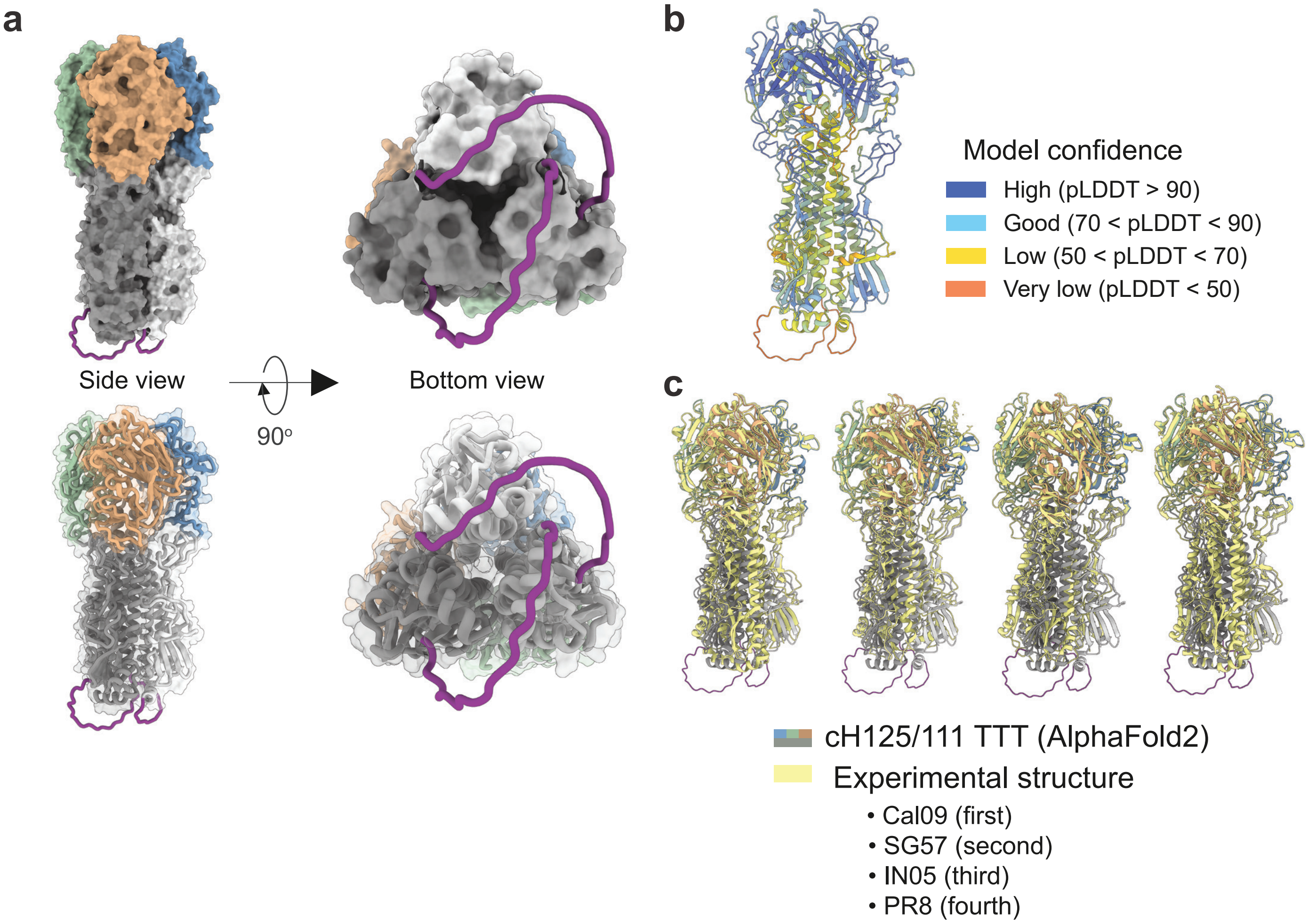

Supplementary Fig. 18 | AlphaFold2-predicted structure of cH125/111 TTT. **a** Predicted cH125/111 TTT structure, with TTT (purple) linkers at the base of the trimer and NL09 (A/Netherlands/602/2009(H1N1)), SG57 (A/Singapore/1/1957(H2N2)), IN05 (A/Indonesia/5/2005(H5N1)) and PR8 (A/Puerto Rico/8/1934/Mount Sinai(H1N1)) regions colored in blue, green, orange and grey, respectively. **b** Predicted structure colored according to the per-residue estimate of the local confidence of the prediction (pLDDT value). **c** Alignment of the predicted cH125/111 TTT structure to the experimentally determined structures of Cal09 (A/California/04/2009(H1N1), PDB 3LZG), SG57 (PDB 2WR7), IN05 (PDB 4K62) and PR8 (PDB 6WCR) native-like trimers.

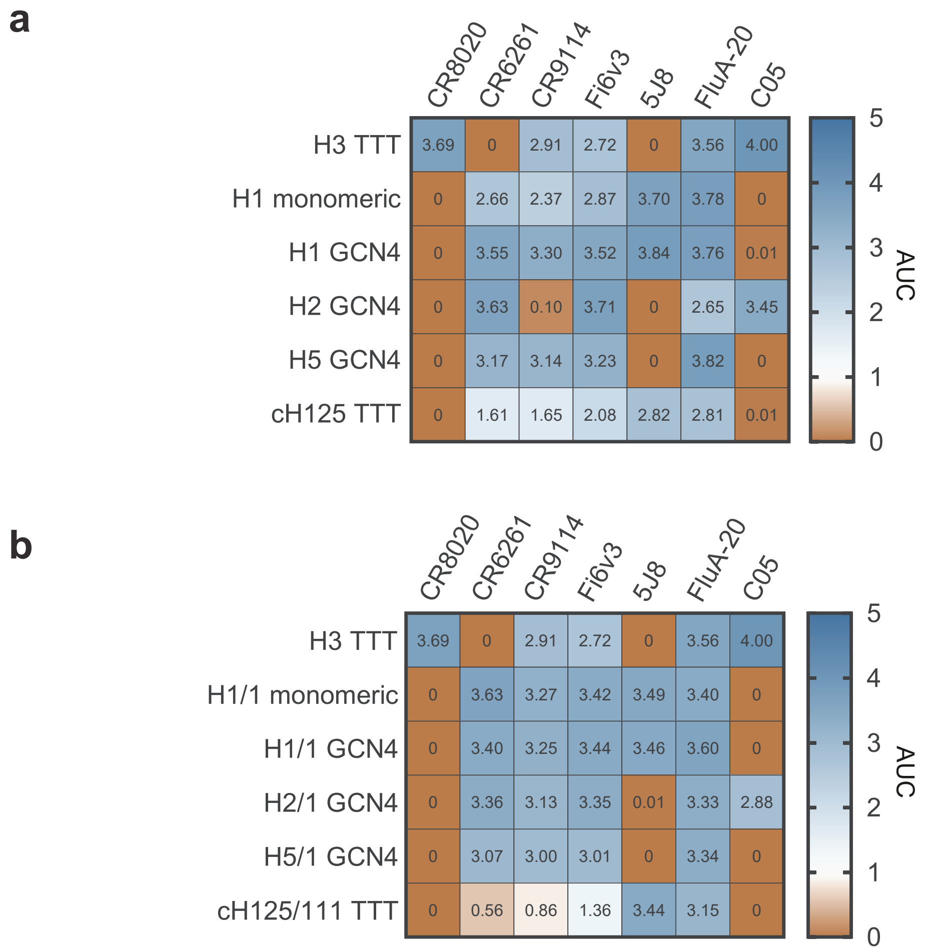

Supplementary Fig. 19 | StrepTactinXT ELISA with supernatants of HEK293T cells transfected with cH125 TTT and ch125/111 TTT against a panel of stem- (CR8020, CR6261, CR9114, FI6v3) and head-specific (5J8, FluA-20, C05). Heatmap values represent the area under the curve (AUC) of the ELISA binding curves.

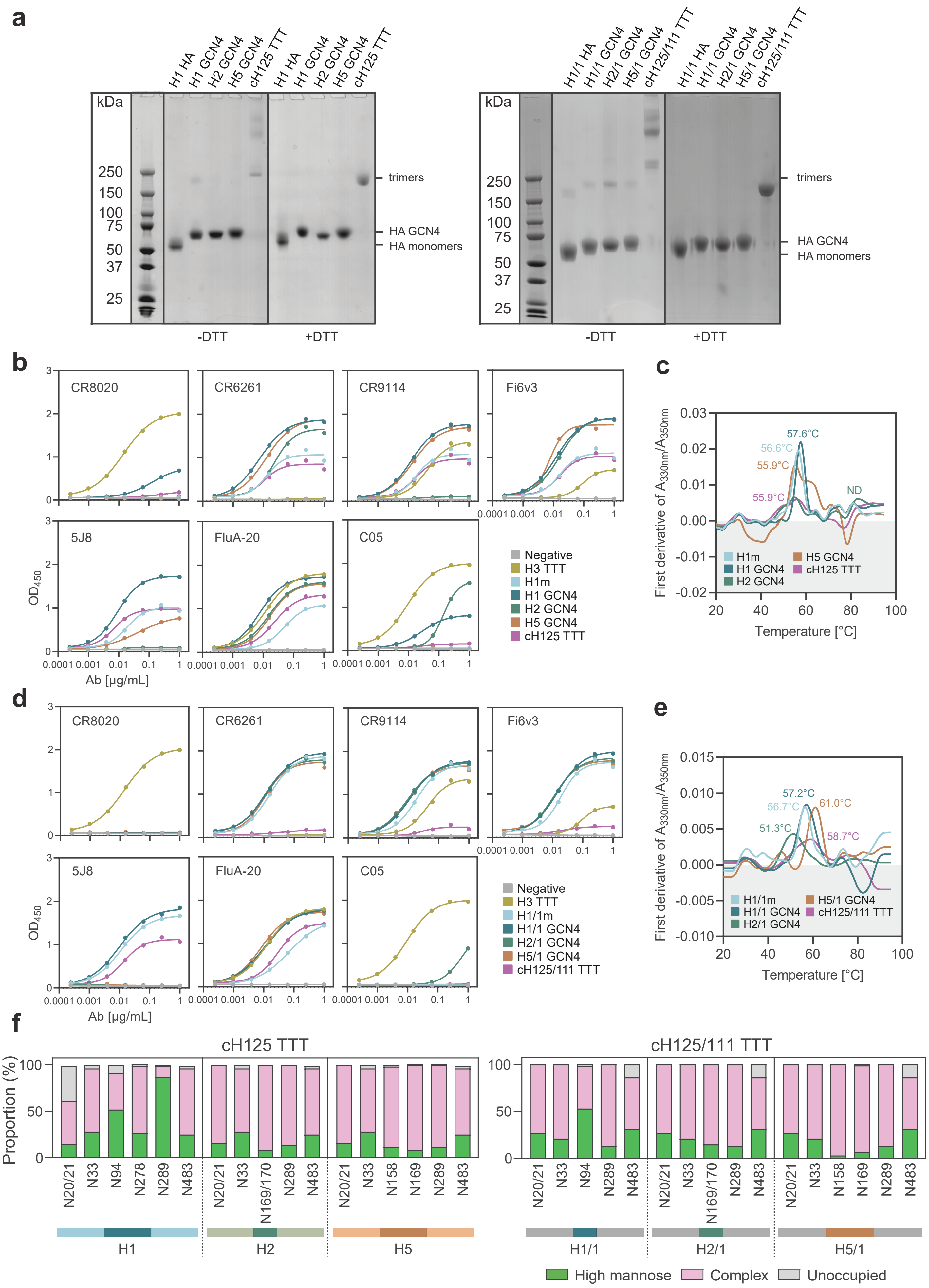

a
H1 HA
H1 GCN4
H2 GCN4
H5 GCN4
cH125 TTT
kDa
250
150
100
75
50
37
25
trimers
HA GCN4
HA monomers
-DTT
+DTT
H1/1 HA
H1/1 GCN4
H2/1 GCN4
H5/1 GCN4
cH125/111 TTT
b
CR8020
CR6261
CR9114
Fi6v3
5J8
FluA-20
C05
OD450
Ab [µg/mL]
Negative
H3 TTT
H1m
H1 GCN4
H2 GCN4
H5 GCN4
cH125 TTT
c
First derivative of A330nm/A350nm
Temperature [°C]
57.6°C
56.6°C
55.9°C
55.9°C
ND
d
H1/1m
H1/1 GCN4
H2/1 GCN4
H5/1 GCN4
cH125/111 TTT
e
57.2°C
56.7°C
61.0°C
51.3°C
58.7°C
f
cH125 TTT
cH125/111 TTT
Proportion (%)
N20/21
N33
N94
N278
N289
N483
N169/170
N158
N169
H1
H2
H5
H1/1
H2/1
H5/1
High mannose
Complex
Unoccupied

Supplementary Fig. 20 | Biophysical characterization of the chimeric HA TTT proteins. a BN-PAGE, non-reducing (-DTT) and reducing (+DTT) SDS-PAGE analyses of StrepTactinXT-purified cH125 TTT and cH125/111 TTT proteins and the corresponding monomeric and GCN4-trimerized control proteins. The single band around 250 kDa in reducing SDS-PAGE confirmed that both cH125 TTT and cH125/111 TTT form covalently linked trimers. **b,d** StrepTactinXT ELISA assay with cH125 TTT **(b)** and cH125/111 TTT **(d)** against a panel of stem- (CR8020, CR6261, CR9114, Fi6v3) and head-specific (5J8, FluA-20, C05). **c,e** Denaturing profile of StrepTactinXT-purified cH125 TTT **(c)** and cH125/111 TTT **(e)** proteins, obtained by nanoDSF and used to determine the T_m values presented on **Table 1. f** Site-specific glycan analysis of StreptactinXT-purified cH125 TTT and cH125/111 TTT proteins. Values are specified in **Supplementary Table 10.** PNGS are displayed according to H3 numbering. The glycan modifications were classified into three categories: high mannose (corresponding to any composition containing two HexNAc residues, or three HexNAc and at least 5 hexoses), complex, or unoccupied. The proportion of peptides and glycopeptides corresponding to each of these categories was colored green for high mannose, pink for complex and grey for unoccupied. The analysis revealed that most sites were effectively populated by glycans, and only the N20/21 site on the first protomer of cH125 TTT and the N483 sites of all three protomers of cH125/111 TTT presented some unoccupancy. The majority of sites were dominated by complex glycans, probably due to the lower density of glycosylation sites compared to the H3 HA TTT protein **(Supplementary Fig. 13d).**

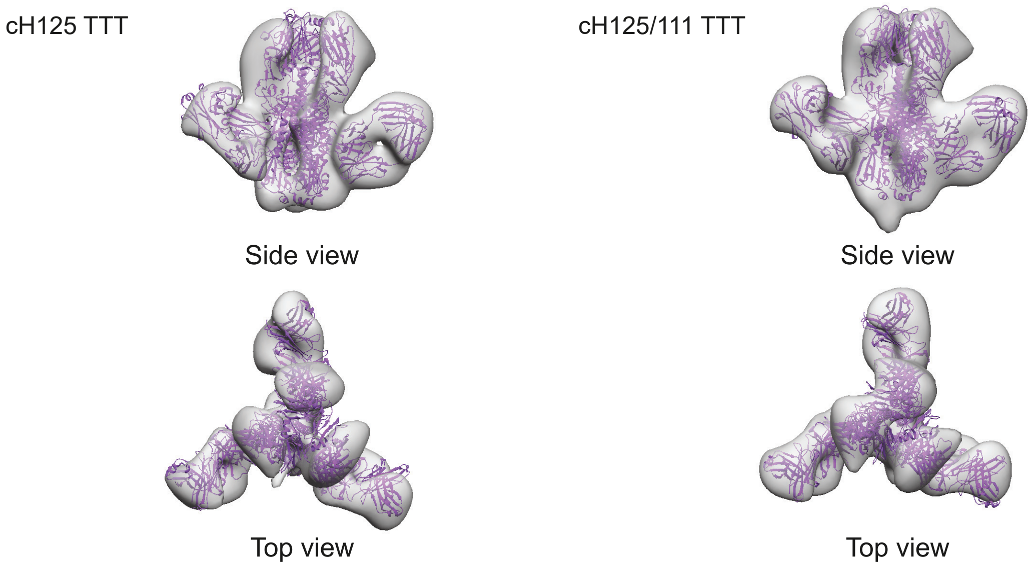

Supplementary Fig. 21 | nsEM-generated 3D models of chimeric HA TTT proteins using C1 symmetry. The models are superimposed to the coordinates of an HA trimer in complex with CR9114 (PDB 4FQV).

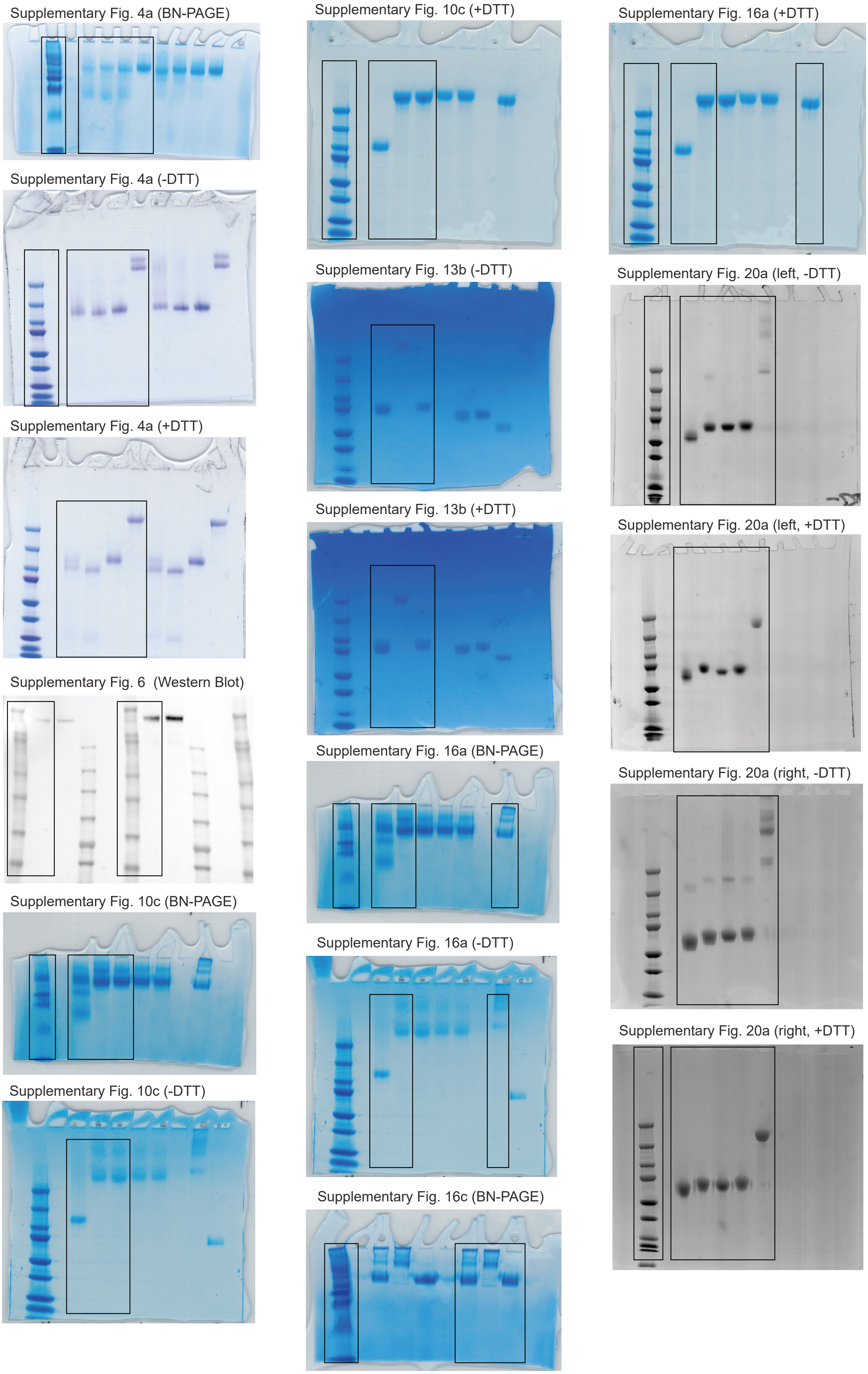

Supplementary Fig. 22 | Uncropped images corresponding to the BN-PAGE, SDS-PAGE and Western Blot analyses. The cropped areas are indicated using black rectangles.

Included Not included

		501C-605C[a]	I559P[a]	R6[a]	ΔMPER[a]	64K/66R[b]	315Q[b]	316W[b]	535M[b]	543Q[b]	543N[b]	72C-564C[c]	73C-561C[c]	49C-555C[c]	47D[d]	49E[d]	65K[d]	106T[d]	165L[d]	429R[d]	432Q[d]	500R[d]	106E[e]	271I[e]	288L[e]	304V[e]	319Y[e]	363Q[e]	519S[e]	568D[e]	570H[e]	585H[e]	569G[f]
SOSIP.v2	SOSIP.v2	Included	Included	Included	Included	Not included	Not included	Not included	Not included	Not included	Not included	Not included	Not included	Not included	Not included	Not included	Not included	Not included	Not included	Not included	Not included	Not included	Not included	Not included	Not included	Not included	Not included	Not included	Not included	Not included	Not included	Not included	Not included
SOSIP.v3	SOSIP.v3.1	Included	Included	Included	Included	Not included	Included	Not included	Included	Included	Not included	Not included	Not included	Not included	Not included	Not included	Not included	Not included	Not included	Not included	Not included	Not included	Not included	Not included	Not included	Not included	Not included	Not included	Not included	Not included	Not included	Not included	Not included
	SOSIP.v3.2	Included	Included	Included	Included	Not included	Included	Not included	Included	Not included	Included	Not included	Not included	Not included	Not included	Not included	Not included	Not included	Not included	Not included	Not included	Not included	Not included	Not included	Not included	Not included	Not included	Not included	Not included	Not included	Not included	Not included	Not included
SOSIP.v4	SOSIP.v4.1	Included	Included	Included	Included	Included	Included	Included	Included	Included	Included	Not included	Not included	Not included	Not included	Not included	Not included	Not included	Not included	Not included	Not included	Not included	Not included	Not included	Not included	Not included	Not included	Not included	Not included	Not included	Not included	Not included	Not included
	SOSIP.v4.2	Included	Included	Included	Included	Not included	Included	Included	Included	Included	Included	Not included	Not included	Not included	Not included	Not included	Not included	Not included	Not included	Not included	Not included	Not included	Not included	Not included	Not included	Not included	Not included	Not included	Not included	Not included	Not included	Not included	Not included
SOSIP.v5	SOSIP.v5.1	Included	Included	Included	Included	Included	Included	Included	Included	Included	Included	Included	Not included	Not included	Not included	Not included	Not included	Not included	Not included	Not included	Not included	Not included	Not included	Not included	Not included	Not included	Not included	Not included	Not included	Not included	Not included	Not included	Not included
	SOSIP.v5.2	Included	Included	Included	Included	Included	Included	Included	Included	Included	Included	Not included	Included	Not included	Not included	Not included	Not included	Not included	Not included	Not included	Not included	Not included	Not included	Not included	Not included	Not included	Not included	Not included	Not included	Not included	Not included	Not included	Not included
SOSIP.v6	SOSIP.v6	Included	Included	Included	Included	Included	Included	Included	Included	Included	Included	Not included	Included	Included	Not included	Not included	Not included	Not included	Not included	Not included	Not included	Not included	Not included	Not included	Not included	Not included	Not included	Not included	Not included	Not included	Not included	Not included	Not included
SOSIP.v7	SOSIP.v7	Included	Included	Included	Included	Included	Included	Included	Included	Included	Included	Not included	Included	Not included	Included	Included	Included	Included	Included	Included	Included	Included	Not included	Not included	Not included	Not included	Not included	Not included	Not included	Not included	Not included	Not included	Not included
SOSIP.v8	SOSIP.v8.1	Included	Included	Included	Included	Included	Included	Included	Included	Included	Included	Not included	Included	Not included	Included	Included	Included	Included	Included	Included	Included	Included	Not included	Not included	Not included	Not included	Not included	Not included	Included	Included	Included	Included	Not included
	SOSIP.v8.2	Included	Included	Included	Included	Included	Included	Included	Included	Included	Included	Not included	Included	Not included	Included	Included	Included	Not included	Included	Included	Included	Included	Included	Included	Included	Included	Included	Included	Included	Included	Included	Included	Not included
	SOSIP.v8.3	Included	Included	Included	Included	Included	Included	Included	Included	Included	Included	Not included	Included	Not included	Included	Included	Included	Not included	Included	Included	Included	Included	Included	Included	Included	Included	Included	Included	Included	Included	Included	Included	Included
	SOSIP.v8.4	Included	Included	Included	Included	Included	Included	Included	Included	Included	Included	Not included	Not included	Not included	Included	Included	Included	Not included	Included	Included	Included	Included	Included	Included	Included	Included	Included	Included	Included	Included	Included	Included	Not included

Supplementary Table 1 | Modifications included in the different SOSIP versions, up to SOSIP.v8.4. Mutations are described in [a]Sanders et al. 2013[7], [b]Dey et al. 2008[8] and de Taeye et al. 2015[9], [c]de la Peña et al. 2017[10], [d]Guenaga et al. 2015[11], [e]Steichen et al. 2016[12], [f]Guenaga et al. 2017[13]. The variant 64K (v4.1) is used for BG505 and B41; while the variant 66R (v4.2) is used for the rest of strains. 72C-564C (v5.1) is an alternative disulfide bond that works as well as 73C-561C (v5.2), but we generally use the latter. All the mutations from Guenaga et al. 2015 are naturally present in BG505 wild-type. The 363Q mutation is not used for BG505 SOSIP.v8.4 trimers, as it introduces a glycan hole.

BG505 TTT	N88	N133	N137	N156	N160	N190	N190c	N197	N234	N262	N276	N295	N301	N332	N339	N355	N363	N386	N392	N398	N406	N411	N448	N462	N611	N618	N625	N637
M9Glc	0	0	0	0	0	0	0	0	0	9	0	0		0	0	0	0	0	0	0		0	0	0	0	0	0	0
M9	0	11	0	63	11	0	0	13	65	66	0	63		83	7	0	24	60	9	0		0	32	0	0	0	0	0
M8	0	41	0	0	13	0	0	2	14	7	7	13		12	6	1	5	10	59	0		0	30	0	1	0	0	8
M7	0	11	0	0	6	0	0	1	3	4	13	0		0	1	2	2	6	9	0		7	10	0	1	0	0	21
M6	3	7	0	0	1	0	0	0	1	2	9	2		1	0	2	1	3	3	0		8	4	0	1	0	1	4
M5	15	12	0	13	8	1	0	2	5	6	33	3		3	1	34	2	7	9	11		30	10	1	6	1	2	14
M4	2	6	0	0	3	0	0	0	0	0	3	4		0	0	1	0	1	2	0		1	3	0	0	0	0	0
M3	0	4	0	0	2	0	0	0	0	0	3	0		0	0	1	0	0	1	0		0	1	0	0	0	0	0
FM	0	0	0	0	0	0	0	0	0	0	0	0		0	0	0	0	0	0	0		0	0	0	0	0	0	0
Hybrid	4	0	0	0	0	0	0	0	0	0	3	0	n.d.	0	0	5	0	1	0	0	n.d.	1	2	0	2	0	3	4
Fhybrid	0	0	0	0	0	0	0	0	0	0	0	0		0	0	5	0	0	0	5		0	0	0	0	0	0	1
HexNAc(3)(x)	6	0	0	0	0	0	0	0	0	0	12	0		0	0	1	0	0	0	0		0	0	0	0	0	0	1
HexNAc(3)(F)(x)	0	0	0	0	1	0	0	0	0	1	2	0		0	0	9	0	0	0	0		1	0	7	0	0	0	0
HexNAc(4)(x)	14	0	0	0	0	0	0	6	0	0	1	0		0	0	0	0	0	0	0		0	0	0	0	0	0	0
HexNAc(4)(F)(x)	11	7	0	0	3	1	0	1	2	2	4	0		0	0	12	1	1	1	41		1	3	68	1	0	0	6
HexNAc(5)(x)	12	0	0	0	0	0	0	16	0	0	0	0		0	0	0	0	0	0	0		0	0	0	0	0	0	0
HexNAc(5)(F)(x)	10	1	0	0	1	1	0	1	0	0	2	0		0	0	8	0	0	0	43		0	0	19	2	0	0	1
HexNAc(6+)(x)	0	0	0	0	0	0	0	4	0	0	0	0		0	0	0	0	0	0	0		0	0	0	0	0	0	0
HexNAc(6+)(F)(x)	1	0	0	0	0	0	0	1	0	0	0	0		0	0	1	0	0	0	0		0	0	1	0	0	0	0
Unoccupied	21	0	100	24	50	95	100	51	9	1	5	14		0	84	18	62	11	4	0		51	3	0	84	99	94	39
Core	0	0	0	0	0	0	0	0	0	1	2	0		0	0	1	0	1	2	0		1	0	2	0	0	0	0

BG505.v4.1	N88	N133	N137	N156	N160	N190	N190c	N197	N234	N262	N276	N295	N301	N332	N339	N355	N363	N386	N392	N398	N406	N411	N448	N462	N611	N618	N625	N637
M9	0			21	4	0		3	62	63	0	79		63	70	0	73	73				0	44	0	1	0		0
M8	0			43	38	2		13	28	34	29	19		23	26	5	22	27				47	43	1	0	0		10
M7	2			31	29	5		12	4	2	22	1		12	1	9	2	0				30	7	1	1	0		15
M6	6			2	14	3		7	2	1	7	0		1	0	5	1	0				10	1	1	1	0		13
M5	23	83	60	1	7	15	3	15	3	0	7	0	99	0	0	15	0	0	100	20	35	10	1	7	0	0	61	9
M4	0			0	1	0		0	0	0	1	0		0	0	3	0	0				0	0	0	0	0		0
M3	0			0	0	0		0	0	0	0	0		0	0	0	0	0				0	0	0	0	0		0
Hybrid	3			0	0	0		3	0	0	7	0		0	0	5	0	0				1	0	0	1	0		8
FHybrid	0			0	0	0		4	0	0	1	0		0	0	9	0	0				0	0	0	0	0		8
A1	6			0	1	1		1	0	0	2	0		0	0	0	0	0				1	0	0	1	0		1
FA1	0			0	0	5		6	0	0	0	0		0	0	7	0	0				1	0	4	2	0		6
A2/A1B	14			0	0	0		0	0	0	7	0		0	0	0	0	0				0	0	0	0	0		0
FA2/FA1B	20	9	19	0	2	31	78	24	1	0	8	0	1	0	1	18	1	0	0	80	65	0	2	38	12	45	11	23
A3/A2B	2			0	0	0		0	0	0	1	0		0	0	0	0	0				0	0	0	0	0		0
FA3/FA2B	22			0	1	26		7	0	0	8	0		0	1	21	0	0				0	0	44	45	49		2
A4/A3B	0			0	0	0		0	0	0	0	0		0	0	0	0	0				0	0	0	0	0		0
FA4/FA3B	1			0	0	3		0	0	0	1	0		0	0	2	0	0				0	0	2	9	6		0
Unoccupied	0	8	21	2	3	9	19	3	0	0	0	0	0	0	0	0	0	0	0	0	0	0	0	0	27	0	28	4

Supplementary Table 2 | Site-specific glycan analysis of GNL-purified BG505 TTT. Values indicate the percentage of each type of glycan (rows) at each glycosylation site (columns) and are represented in **Supplementary Fig. 4d.** Site-specific glycan analysis of 2G12+SEC-purified BG505 SOSIP.v4.1 protein, from a previous study[1], is presented for comparison.

**Data collection**	BG505 TTT + PGT124 Fab + 35O22 scFv
Beamline	SSRL12-1
Wavelength (Å)	0.9795
Space group	C2
Unit cell parameters	
a, b, c (Å)	357.6, 245.0, 207.7
α, β, γ (°)	90, 125.1, 90
Resolution (Å)	50.0-5.80 (5.90-5.80)[a]
Unique reflections	34,433 (3,202)[a]
Redundancy	6.8 (5.7)[a]
Completeness (%)	97.8 (94.0)[a]
$\langle I/\sigma_I \rangle$	12.5 (1.0)[a]
R_{sym}[b] (%)	22.1 (>100)[a]
R_{pim}[b] (%)	9.1 (79.4)[a]
$CC_{1/2}$[c] (%)	99.2 (66.5)[a]
**Refinement statistics**	
Resolution (Å)	40.9-5.80
Reflections (work)	34,133
Reflections (test)	3,169
R_{cryst}[d] / R_{free}[e] (%)	25.5/29.2
No. of atoms	29,653
Average *B*-values (Å^2)	359
Wilson *B*-value (Å^2)	224
**RMSD from ideal geometry**	
Bond length (Å)	0.003
Bond angle (°)	0.57
**Ramachandran statistics (%)**[f]	
Favored	94.6
Outliers	0.2
**PDB code**	8TGO

[a] Numbers in parentheses refer to the highest-resolution shell.
[b] $R_{sym} = \Sigma_{hkl} \Sigma_i | I_{hkl,i} - \langle I_{hkl} \rangle | / \Sigma_{hkl} \Sigma_i I_{hkl,i}$ and $R_{pim} = \Sigma_{hkl} (1/(n-1))^{1/2} \Sigma_i | I_{hkl,i} - \langle I_{hkl} \rangle | / \Sigma_{hkl} \Sigma_i I_{hkl,i}$, where $I_{hkl,i}$ is the scaled intensity of the ith measurement of reflection h, k, l, $\langle I_{hkl} \rangle$ is the average intensity for that reflection, and *n* is the redundancy.
[c] $CC_{1/2}$ = Pearson correlation coefficient between two random half datasets.
[d] $R_{cryst} = \Sigma_{hkl} | F_o - F_c | / \Sigma_{hkl} | F_o | \times 100$, where F_o and F_c are the observed and calculated structure factors, respectively.
[e] R_{free} was calculated as for R_{cryst}, but on a test set comprising 10% of the data excluded from refinement.
[f] From MolProbity.

Supplementary Table 3 | X-ray data collection and refinement statistics.

Group	Regimen	Mouse	Wk2			Wk6			Wk10		
			SOSIP.664	SOSIP.v8	gp120	SOSIP.664	SOSIP.v8	gp120	SOSIP.664	SOSIP.v8	gp120
1	SOSIP.664 (C/M/P)	M1	<50	<50	68	10489	137	72932	23194	188	113316
		M2	<50	<50	<50	11930	579	23067	4959	124	6651
		M3	<50	<50	<50	3754	77	3501	3218	105	11948
		M4	81	<50	<50	28634	2552	1379	29485	7208	54414
		M5	<50	<50	57	9736	207	12997	6034	150	15939
		M6	<50	<50	58	4457	141	15462	1741	161	968
		M7	308	50	1251	8613	2617	24206	18429	881	55631
		M8	<50	<50	52	1020	480	463	537	207	1503
		M9	<50	126	253	8592	134	77461	11330	482	56062
		M10	285	<50	60	460	114	135	13412	217	80076
		M11	124	<50	1525	9400	218	221371	367	66	63
		M12	65	75	125	10297	694	9165	7781	886	11475
2	TTT (C/M/P)	M13	50	71	74	3639	547	75	171	329	65
		M14	64	130	55	808	1585	69	1242	249	74
		M15	64	57	137	90	177	66	100	304	65
		M16	61	68	77	83	56	<50	975	1120	61
		M17	70	<50	56	139	354	55	567	830	136
		M18	72	<50	61	169	166	63	137	136	92
		M19	<50	55	65	836	426	72	109639	34122	160
		M20	<50	54	113	283	163	103	56939	6092	147
		M21	172	54	58	107	73	86	178	193	70
		M22	70	65	79	678	157	55	9950	1015	59
		M23	82	51	67	212	910	112	1088	1839	135
		M24	110	114	55	30145	5355	98	8313	2750	73

Binding endpoint titers	
(white)	<50(LOD)
(grey)	50-500
(light yellow)	501-5000
(orange)	5001-50000
(dark orange)	>50000

Supplementary Table 4 | Endpoint antibody-binding titers of mice sera over time against SOSIP.664, SOSIP.v8 and gp120 proteins, as measured by D7324-capture ELISA. Values are represented in **Supplementary Fig. 7a.**

			Wk10			Wk26		
Group	Regimen	Rabbit	SOSIP.664	SOSIP.v8	gp120	SOSIP.664	SOSIP.v8	gp120
1A	SOSIP.664 (C/M/P)	37184	12191	116	51929	19542	595	120036
		37185	3467	132	12416	8589	635	74675
		37186	30726	182	95982	12565	197	71857
		37187	7247	203	34014	8309	1661	69168
		37188	9650	270	24601	22292	3613	94765
		37189	3115	315	11244	2746	422	13058
1B	SOSIP.664 (C/M/P^G)	37202	9769	197	48897	19829	1393	96015
		37203	5150	327	25881	17523	3313	94316
		37204	14279	310	54623	2555	265	8518
		37205	5114	146	33514	5041	292	30397
		37206	15088	374	44583	24623	1042	143830
		37207	3922	157	11319	4505	300	18232
2A	TTT (C/M/P)	37190	1716	1551	139	6382	1398	1563
		37191	883	909	1589	12282	4186	18928
		37192	2415	1764	390	8428	2555	20111
		37193	1072	779	835	4342	1337	5338
		37194	2071	1145	1050	11946	2714	23261
		37195	3037	662	323	29300	5687	10871
2B	TTT (C/M/P^G)	37208	868	679	411	12132	10492	9637
		37209	2598	942	1994	19573	9008	30734
		37210	1829	1320	1246	10707	10930	7696
		37211	602	320	487	13199	11086	31968
		37212	8207	2420	11940	30531	23966	118372
		37213	1548	506	1236	23152	16771	49400
3	SOSIP.664 (P/P/P)	37178	6335	593	7576	14475	4279	28326
		37179	29677	1167	36364	100974	18846	112702
		37180	3512	99	1687	4148	1316	11224
		37181	10317	149	12351	14733	2431	77063
		37182	4059	123	12401	ND	ND	ND
		37183	1331	181	925	2361	339	9322
4	TTT (CP/MP/P)	37196	17883	2419	18842	27284	8363	49040
		37197	57186	6378	106234	37230	7382	59086
		37198	15147	1520	20701	17897	4324	34182
		37199	76859	3993	135124	15740	2866	36042
		37200	77210	3332	62310	17786	3573	36404
		37201	282744	5904	182871	15834	3097	38028
5	TTT.GM (C/M/P^G)	37214	1190	966	689	38943	13835	7779
		37215	904	473	2902	9842	8234	13053
		37216	64	90	122	ND	ND	ND
		37217	1494	1478	2453	20808	12474	39662
		37218	744	530	906	27926	24225	23464
		37219	1139	532	2602	5825	4884	7664

Binding endpoint titers	
(white)	<50(LOD)
(grey)	50-500
(light yellow)	501-5000
(orange)	5001-50000
(dark orange)	>50000

Supplementary Table 5 | Endpoint antibody binding titers of rabbit at weeks 10 and 26 against SOSIP.664, SOSIP.v8 and gp120 proteins, as measured by D7324-capture ELISA. Values are represented in **Supplementary Fig. 8a.**

Group	Regimen	Rabbit	Wk10 MLV Neg. Control	Wk10 BG505 Clade A Tier 2	Wk26 MLV Neg. Control	Wk26 BG505 Clade A Tier 2	Wk42 MLV Neg. Control	Wk42 BG505 Clade A Tier 2
1A	SOSIP.664 (C/M/P)	37184	<5	<5	<20	<20	<20	<20
		37185	<5	<5	<20	<20	<20	<20
		37186	<5	<5	<20	<20	<20	<20
		37187	5	216	<20	2468	<20	1154
		37188	<5	<5	<20	934	<20	415
		37189	<5	<5	<20	<20	<20	<20
1B	SOSIP.664 (C/M/P^G)	37202	14	33	<20	151	<20	<20
		37203	6	14	<20	43	<20	855
		37204	<5	9	<20	223	<20	572
		37205	<5	<5	<20	<20	<20	<20
		37206	<5	5	<20	246	<20	97
		37207	<5	<5	<20	<20	<20	<20
2A	TTT (C/M/P)	37190	<5	<5	<20	<20	<20	<20
		37191	<5	<5	<20	420	<20	1442
		37192	<5	<5	<20	<20	<20	96
		37193	<5	53	<20	257	<20	3313
		37194	<5	15	<20	<20	<20	9282
		37195	<5	<5	<20	210	<20	<20
2B	TTT (C/M/P^G)	37208	<5	<5	<20	<20	<20	<20
		37209	<5	<5	40	82	<20	<20
		37210	<5	<5	<20	<20	<20	<20
		37211	<5	<5	<20	<20	<20	<20
		37212	<5	2277	<20	14611	<20	24440
		37213	<5	<5	<20	<20	<20	<20
3	SOSIP.664 (P/P/P)	37178	<5	<5	<20	717	<20	3097
		37179	<5	541	<20	2398	<20	1509
		37180	<5	<5	<20	<20	<20	<20
		37181	<5	36	<20	834	<20	104
		37182	<5	<5	ND	ND	ND	ND
		37183	<5	<5	<20	<20	<20	45
4	TTT (CP/MP/P)	37196	<5	<5	<20	1598	<20	376
		37197	<5	25	<20	1223	<20	326
		37198	30	72	<20	<20	<20	<20
		37199	<5	81	<20	172	<20	671
		37200	<5	<5	<20	267	<20	140
		37201	<5	9	<20	72	<20	264
5	TTT.GM (C/M/P^G)	37214	<5	<5	<20	<20	<20	<20
		37215	<5	<5	<20	<20	<20	<20
		37216	<5	<5	ND	ND	ND	ND
		37217	<5	<5	<20	<20	<20	<20
		37218	<5	<5	<20	<20	<20	<20
		37219	<5	<5	<20	<20	<20	<20

Neutralization titers (ID50)
<LOD
LOD-40
41-100
101-1000
>1000

Supplementary Table 6 | Midpoint neutralization titers (ID_{50}) over time for sera of the immunized rabbits against an autologous BG505/T332N pseudovirus. ID_{50} values were determined in a TZM-bl neutralization assay and are represented in **Supplementary Fig. 8b.**

BG505 TTT.GM	N88	N133	N137	N156	N160	N190	N190c	N197	N234	N241	N262	N276	N289	N295	N301	N332	N339	N355	N363	N386	N392	N398	N406	N411	N448	N462	N611	N618	N625	N637	Linker
M9Glc	0	0	0	n.d.	0	0	n.d.	0	n.d.	0	2	0	0	0	0	0	0	0	0	0	0	0	n.d.	0	0	0	0	0	0	0	0
M9	0	0	0		10	0		1		0	40	0	50	0	0	47	15	0	35	23	0	0		0	22	0	1	0	0	0	0
M8	1	0	0		36	0		12		27	30	1	0	0	0	23	6	1	21	23	31	0		12	17	0	1	0	1	3	0
M7	4	0	0		10	0		4		24	8	5	43	0	0	2	1	1	5	9	5	0		13	8	0	7	1	0	5	0
M6	13	0	0		1	1		3		21	6	16	0	0	0	4	0	2	2	6	3	0		0	7	0	2	0	3	7	0
M5	15	55	0		2	10		11		28	4	26	7	100	100	10	2	19	6	16	14	0		37	17	15	12	1	1	25	0
M4	0	7	0		0	0		1		0	0	6	0	0	0	1	0	2	0	2	2	0		0	3	0	3	0	0	1	0
M3	0	0	0		0	1		1		0	0	1	0	0	0	1	0	0	1	2	3	0		1	1	0	0	0	0	0	0
FM	0	0	0		0	1		0		0	0	0	0	0	0	0	0	0	0	0	0	0		0	0	0	6	1	0	0	0
Hybrid	15	0	0		1	1		2		0	0	1	0	0	0	1	0	3	1	3	0	0		0	2	0	3	0	2	6	0
Fhybrid	0	0	0		0	1		2		0	0	5	0	0	0	1	0	2	1	0	2	0		0	1	1	2	0	0	1	0
HexNAc(3)(x)	5	0	0		0	0		1		0	0	3	0	0	0	0	0	1	0	0	0	0		0	0	0	5	2	5	0	0
HexNAc(3)(F)(x)	1	1	0		0	2		3		0	0	4	0	0	0	1	0	8	1	0	3	43		5	2	1	1	0	0	1	0
HexNAc(4)(x)	11	0	0		0	0		8		0	0	0	0	0	0	0	0	0	0	0	0	0		0	0	0	0	0	0	0	0
HexNAc(4)(F)(x)	10	34	0		1	8		3		0	6	31	0	0	0	4	1	29	5	4	21	0		30	11	42	13	7	0	5	0
HexNAc(5)(x)	6	0	0		0	0		23		0	0	0	0	0	0	0	0	0	0	0	0	0		0	0	0	0	0	0	0	0
HexNAc(5)(F)(x)	2	0	0		0	6		2		0	3	0	0	0	0	4	0	8	2	1	6	57		0	1	34	8	0	0	2	0
HexNAc(6+)(x)	1	0	0		0	0		10		0	0	0	0	0	0	0	0	0	0	0	0	0		0	0	0	0	0	0	0	0
HexNAc(6+)(F)(x)	1	2	0		0	1		0		0	1	0	0	0	0	0	0	9	0	0	0	0		0	0	3	3	0	0	0	0
Unoccupied	14	0	100		38	64		13		0	0	0	0	0	0	0	73	14	19	7	7	0		0	7	1	32	83	87	44	100
Core	0	0	0		0	3		1		0	0	0	0	0	0	1	0	1	1	4	3	0		0	1	2	1	1	0	0	0

Supplementary Table 7 | Site-specific glycan analysis of the GNL-purified BG505 TTT.GM protein preparation. Values indicate the percentage of each type of glycan (rows) at each glycosylation site (columns) and are represented in **Supplementary Fig. 10g.**

HA TTT	N22	N38	N45	N63	N122	N126	N133	N144	N165	N246	N285	N483
M9Glc	n.d.	0	0	0	0	0	0	0	0	0	0	0
M9		0	0	0	0	0	0	0	8	20	2	0
M8		0	0	0	0	1	4	0	13	17	5	0
M7		0	0	0	0	1	0	0	13	2	8	0
M6		0	6	0	0	2	0	0	10	2	6	0
M5		2	27	7	0	30	96	0	38	24	44	0
M4		0	0	0	0	0	0	0	0	0	0	0
M3		0	0	0	0	0	0	0	0	0	0	0
FM		0	0	0	0	0	0	0	0	0	0	1
Hybrid		0	19	1	0	2	0	0	5	6	5	0
Fhybrid		0	0	0	0	3	0	0	1	4	0	0
HexNAc(3)(x)		0	29	1	0	1	0	0	1	2	5	0
HexNAc(3)(F)(x)		3	1	2	0	10	0	0	3	3	8	0
HexNAc(4)(x)		0	7	1	0	2	0	0	1	5	0	0
HexNAc(4)(F)(x)		38	4	36	0	20	0	0	4	7	9	40
HexNAc(5)(x)		0	7	3	0	0	0	0	0	2	0	0
HexNAc(5)(F)(x)		29	0	45	0	19	0	0	3	5	0	36
HexNAc(6+)(x)		0	0	0	0	0	0	0	0	0	0	0
HexNAc(6+)(F)(x)		9	0	0	0	0	0	0	0	0	0	2
Unoccupied		15	0	1	100	10	0	100	0	0	1	19
Core		3	0	2	0	0	0	0	1	0	6	2

Supplementary Table 8 | Site-specific glycan analysis of the StreptactinXT-purified H3 HA TTT protein preparation. Values indicate the percentage of each type of glycan (rows) at each glycosylation site (columns) and are represented in **Supplementary Fig. 13d.**

BG505 protomer	N88	N133	N142	N156	N160	N190	N190c	N197	N234	N241	N262	N276	N289	N295	N301	N332	N339	N355	N363	N386	N392	N398	N406	N411	N448	N462	N611	N618	N625	N637
M9Glc	0	0	n.d.	0	0	0	n.d.	0	n.d.	n.d.	0	0	0	n.d.	n.d.	0	0	0	0	0	0	0	n.d.	0	0	0	0	0	0	0
M9	0	0		0	0	0		10			67	1	22			8	14	1	8	11	16	0		0	62	0	0	0	0	0
M8	1	0		15	3	0		48			28	24	25			2	7	4	2	3	27	0		3	18	0	0	0	0	4
M7	3	0		5	3	0		16			4	19	15			0	1	6	0	1	4	0		0	6	0	0	0	0	10
M6	15	0		0	3	0		7			0	14	20			0	0	7	0	1	2	0		4	11	0	0	0	0	8
M5	16	11		0	4	0		0			0	31	7			0	0	42	0	2	3	3		7	2	0	0	0	0	7
M4	3	0		0	1	0		0			0	1	2			0	0	2	0	0	1	0		0	0	0	0	0	0	1
M3	0	0		0	0	0		0			0	0	1			0	0	0	0	0	0	0		0	0	0	0	0	0	0
FM	0	0		0	0	0		0			0	0	0			0	0	0	0	0	0	1		0	0	0	0	0	0	0
Hybrid	25	0		0	0	0		0			0	7	0			0	0	9	0	0	0	0		0	0	0	0	0	0	5
Fhybrid	0	0		0	0	0		0			0	0	0			0	0	6	0	0	0	0		0	0	0	0	0	0	4
HexNAc(3)(x)	5	0		0	0	0		0			0	1	0			0	0	1	0	0	0	0		0	0	0	0	0	0	0
HexNAc(3)(F)(x)	0	0		0	0	0		0			0	0	0			0	0	7	0	0	0	24		0	0	6	0	1	0	7
HexNAc(4)(x)	26	0		0	0	0		0			0	1	0			0	0	0	0	0	0	0		0	0	0	0	0	0	0
HexNAc(4)(F)(x)	2	0		0	0	0		0			0	0	0			0	0	6	0	0	0	29		0	0	33	0	4	0	19
HexNAc(5)(x)	3	0		0	0	0		0			0	0	0			0	0	0	0	0	0	0		0	0	0	0	0	0	0
HexNAc(5)(F)(x)	1	0		0	0	0		0			0	0	0			0	0	5	0	0	0	44		0	0	25	3	3	0	2
HexNAc(6+)(x)	0	0		0	0	0		0			0	0	0			0	0	0	0	0	0	0		0	0	0	0	0	0	0
HexNAc(6+)(F)(x)	0	0		0	0	0		0			0	0	0			0	0	0	0	0	0	0		0	0	0	0	0	0	0
Unoccupied	0	0		41	31	100		16			1	0	6			2	14	1	11	31	2	0		29	0	0	92	63	97	8
Core	0	89		39	55	0		3			0	1	2			87	64	3	78	51	46	0		58	1	35	5	28	1	26

conM protomer	N88	N130	N136	N141	N156	N160	N188	N197	N234	N241	N262	N276	N289	N295	N301	N332	N339	N356	N386	N392	N403	N406	N448	N461	N611	N616	N625	N637
M9Glc	0	0	n.d.	n.d.	n.d.	n.d.	0	0	n.d.	n.d.	0	0	0	n.d.	0	n.d.	n.d.	0	0	0	n.d.	n.d.	0	n.d.	0	n.d.	0	0
M9	0	0					0	0			66	2	1		0			0	23	4			80		0		0	11
M8	0	0					0	0			27	54	5		0			1	29	8			13		0		0	18
M7	0	0					0	0			6	21	6		4			0	13	0			3		0		0	12
M6	5	0					0	0			0	9	3		6			0	9	0			1		0		0	14
M5	15	2					0	2			0	10	5		6			2	5	1			1		2		0	10
M4	0	0					0	0			0	1	0		40			0	1	0			0		0		0	1
M3	0	0					0	0			0	0	0		5			0	0	0			0		0		0	0
FM	0	0					0	0			0	0	0		0			0	0	0			0		0		0	0
Hybrid	0	0					0	1			0	2	0		8			0	0	0			0		0		0	7
Fhybrid	0	0					0	1			0	0	0		9			2	0	0			0		0		0	2
HexNAc(3)(x)	0	1					0	0			0	0	0		0			0	0	0			0		0		0	0
HexNAc(3)(F)(x)	0	11					0	3			0	0	0		11			6	0	0			0		0		0	0
HexNAc(4)(x)	14	0					0	0			0	0	0		0			0	0	0			0		0		0	0
HexNAc(4)(F)(x)	53	50					0	7			0	0	0		8			27	0	0			0		0		0	2
HexNAc(5)(x)	0	0					0	0			0	0	0		0			0	0	0			0		0		0	0
HexNAc(5)(F)(x)	13	36					0	2			0	0	0		0			61	0	0			0		0		0	0
HexNAc(6+)(x)	0	0					0	0			0	0	0		0			0	0	0			0		0		0	0
HexNAc(6+)(F)(x)	0	0					0	0			0	0	0		0			0	0	0			1		0		0	0
Unoccupied	0	0					99	6			1	0	80		0			0	20	2			0		93		98	20
Core	0	0					0	77			0	0	0		3			0	1	84			0		5		1	1

AMC011 protomer	N88	N130	N136	N141	N142c	N156	N160	N188	N197	N234	N241	N262	N276	N289	N295	N301	N332	N339	N355	N362	N386	N392	N396	N406	N448	N460	N611	N616	N625	N637
M9Glc	0	0	0	n.d.	n.d.	0	n.d.	0	0	n.d.	n.d.	0	0	0	n.d.	n.d.	0	0	n.d.	0	0	0	n.d.	0	0	n.d.	0	0	0	0
M9	0	0	0			0		1	47			10	0	45			3	37		46	29	4		0	63		0	0	0	0
M8	0	0	0			0		7	34			6	63	19			0	15		8	8	8		0	18		0	0	0	1
M7	0	0	0			0		20	7			2	23	11			0	0		2	3	0		0	6		0	0	0	1
M6	0	0	0			0		20	0			3	1	6			0	5		1	1	0		0	10		0	0	0	4
M5	21	2	0			0		31	0			1	13	3			1	2		1	2	1		0	2		0	0	6	3
M4	0	0	0			0		0	0			0	1	1			0	0		0	0	0		0	0		0	0	0	1
M3	0	0	0			0		0	0			2	0	0			0	0		0	0	0		0	0		0	0	0	0
FM	0	0	0			0		0	0			0	0	0			0	0		0	0	0		35	0		0	0	0	0
Hybrid	0	0	0			0		12	0			0	0	0			0	0		0	0	0		0	0		0	0	0	1
Fhybrid	0	0	0			0		3	0			0	0	0			0	0		0	0	0		0	0		0	0	0	0
HexNAc(3)(x)	0	1	90			0		1	0			0	0	0			0	0		0	0	0		0	0		0	0	0	0
HexNAc(3)(F)(x)	0	11	10			0		1	0			0	0	0			0	0		0	0	0		45	0		0	0	0	0
HexNAc(4)(x)	16	0	0			0		0	0			0	0	0			0	0		0	0	0		0	0		0	0	0	0
HexNAc(4)(F)(x)	41	50	0			0		0	0			0	0	0			0	0		0	0	0		20	0		0	0	0	1
HexNAc(5)(x)	0	0	0			0		0	0			0	0	0			0	0		0	0	0		0	0		0	0	0	0
HexNAc(5)(F)(x)	22	36	0			0		0	0			0	0	0			0	0		0	0	0		0	0		0	0	0	0
HexNAc(6+)(x)	0	0	0			0		0	0			0	0	0			0	0		0	0	0		0	0		0	0	0	0
HexNAc(6+)(F)(x)	0	0	0			0		0	0			0	0	0			0	0		0	0	0		0	0		0	0	0	0
Unoccupied	0	0	0			0		1	11			0	0	13			0	41		40	0	2		0	0		100	100	94	87
Core	0	0	0			100		2	0			75	0	1			96	0		1	57	84		0	1		0	0	0	0

Supplementary Table 9 | Site-specific glycan analysis of the PGT145-purified chEnv TTT protein preparation. Values indicate the percentage of each type of glycan (rows) at each glycosylation site (columns) and are represented in **Supplementary Fig. 16f.**

cH125 TTT	N20/21[1]	N20/21[2,3]	N33[1,2,3]	N94[1]	N158[3]	N169[3]	N169/170[2]	N278[1]	N289[1]	N289[2]	N289[3]	N483[1,2,3]
M9Glc	0	0	0	0	0	0	0	0	0	0	0	0
M9	0	0	0	0	0	0	0	0	2	0	0	0
M8	0	1	0	5	0	0	0	6	12	1		1
M7	0	3	2	15	1	0	0	5	6	1	0	1
M6	0	1	1	5	1	0	0	1	6	1	1	1
M5	7	10	22	23	8	7	6	13	51	9	10	19
M4	7	0	0	0	1	0	1	0	3	1	1	1
M3	2	0	1	0	1	0	0	0	0	0	0	1
FM	0	0	2	0	1	1	1	0	0	0	0	1
Hybrid	0	0	0	2	0	0	0	1	6	1	0	1
Fhybrid	0	0	0	1	0	0	0	0	0	0	0	0
HexNAc(3)(x)	0	1	1	1	0	0	0	2	2	1	0	1
HexNAc(3)(F)(x)	2	0	4	2	4	3	3	2	0	4	2	4
HexNAc(4)(x)	0	1	1	2	0	0	1	2	2	2	0	3
HexNAc(4)(F)(x)	2	20	32	23	25	40	40	40	2	38	29	28
HexNAc(5)(x)	0	1	0	1	0	0	0	0	0	1	1	1
HexNAc(5)(F)(x)	3	15	16	10	29	46	33	19	0	23	29	13
HexNAc(6+)(x)	0	0	0	0	0	0	0	0	0	0	0	1
HexNAc(6+)(F)(x)	0	33	12	0	5	0	4	0	0	6	12	11
Unoccupied	38	0	4	9	2	1	0	2	1	0	1	3
Core	39	14	1	0	22	2	10	5	4	12	13	9

[1]Present in the H1 protomer [2]Present in the H2 protomer [3]Present in the H5 protomer

cH125/111 TTT	N20/21[1,2,3]	N33[1,2,3]	N94[1]	N158[3]	N169[3]	N169/170[2]	N289[1,2,3]	N483[1,2,3]
M9Glc	0	0	0	0	0	0	0	0
M9	0	2	0	0	0	0	0	1
M8	0	3	0	0	0	2	1	3
M7	2	2	4	0	1	2	0	4
M6	1	1	5	0	1	2	1	4
M5	14	5	27	2	3	8	6	13
M4	7	2	0	1	1	1	0	0
M3	0	1	0	0	0	0	0	0
FM	0	0	0	0	0	0	0	0
Hybrid	0	2	17	0	0	1	3	4
Fhybrid	3	2	0	0	0	0	1	2
HexNAc(3)(x)	0	1	0	0	0	0	1	2
HexNAc(3)(F)(x)	1	4	3	1	2	0	4	3
HexNAc(4)(x)	3	0	5	0	0	0	1	1
HexNAc(4)(F)(x)	36	34	25	40	44	51	47	32
HexNAc(5)(x)	0	0	1	0	0	1	0	0
HexNAc(5)(F)(x)	18	20	10	50	39	28	22	13
HexNAc(6+)(x)	0	0	0	0	0	0	0	0
HexNAc(6+)(F)(x)	15	16	0	5	5	3	10	4
Unoccupied	0	0	2	0	1	0	0	14
Core	0	3	0	0	2	1	3	0

[1]Present in the H1/1 protomer [2]Present in the H2/1 protomer [3]Present in the H5/1 protomer.

Supplementary Table 10 | Site-specific glycan analyses of the StrepTactinXT-purified cH125 and cH125/111 TTT protein preparations. Values indicate the percentage of each type of glycan (rows) at each glycosylation site (columns) and are represented in **Supplementary Fig. 20d.**

Protein	+CR9114	+5J8	+2B05	2D class averages	3D model	Comments
H1	Full	Partial	-	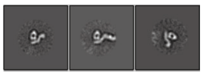	-	Sample contains mostly monomers. All particles bind CR9114, but only a portion bind 5J8.
H1 GCN4	3/3	1/3	-	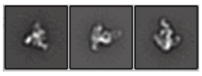	-	-
	-	-	3/3	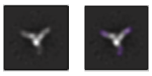	-	**Evidence of cH125/TTT chimerism:** particles bind 3x2B05 Fabs (H1 head-specific)
H2 GCN4	-	-	-	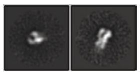	-	-
	0/3	-	-	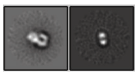	-	No CR9114 binding, consistent with ELISA data.
	-	0/3	-	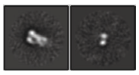	-	No 5J8 binding, consistent with ELISA data.
	-	-	0/3	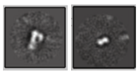	-	**Evidence of cH125/TTT chimerism:** no 2B05 binding (H1 head-specific).
H5 GCN4	3/3	-	-	-	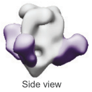	-
	-	0/3	-	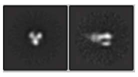	-	No 5J8 binding, consistent with ELISA data.
	-	-	0/3	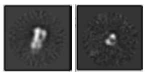	-	**Evidence of cH125/TTT chimerism:** no 2B05 binding (H1 head-specific).
cH125 TTT	3/3	0/3	-	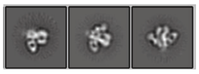	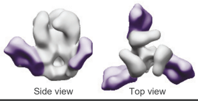	No 5J8 binding observed, in contrast with ELISA data.
H1/1	Full	Partial	-	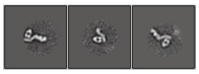	-	Sample contains mostly monomers. All particles bind CR9114, but only a portion bind 5J8.
H1/1 GCN4	3/3	Partial	-	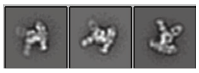	-	Sample contains only trimers, with full occupancy of CR9114 epitopes, but partical occupancy of 5J8 epitopes (1-2x5J8 per particle).
H2/1 GCN4	3/3	1/3	-	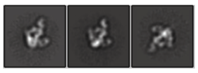	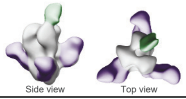	Most particles bound by 1x5J8 Fab, in contrast with ELISA data.
H5/1 GCN4	3/3	0/3	-	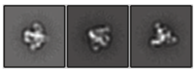	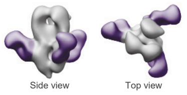	-
	-	0/3	-	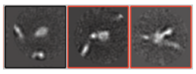	-	5J8 induced trimer degradation.
cH125/111 TTT	3/3	0/3	-	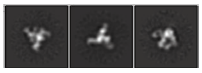	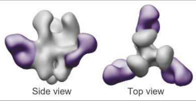	No 5J8 binding observed, in contrast with ELISA data.
	-	-	1/3	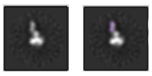	-	**Evidence of cH125/TTT chimerism:** particles bind 1x2B05 Fab (H1 head-specific).

Supplementary Table 11 | nsEM data of cH125 TTT, cH125/111 TTT and the monomeric and GCN4-trimerized controls. Proteins were imaged independently or complexed with CR9114, 5J8 or 2B05 fabs. Columns 2-4 indicate the occupancy of epitopes for the corresponding complexed antibody. 3D models were generated using C3 symmetry.

Supplementary References

1. Derking, R. *et al.* Enhancing glycan occupancy of soluble HIV-1 envelope trimers to mimic the native viral spike. *Cell Rep.* **35**, 108933 (2021).
2. deCamp, A. *et al.* Global panel of HIV-1 Env reference strains for standardized assessments of vaccine-elicited neutralizing antibodies. *J. Virol.* **88**, 2489–2507 (2014).
3. Seabright, G. E. *et al.* Networks of HIV-1 Envelope Glycans Maintain Antibody Epitopes in the Face of Glycan Additions and Deletions. *Structure* **28**, 897–909.e6 (2020).
4. An, Y., McCullers, J. A., Alymova, I., Parsons, L. M. & Cipollo, J. F. Glycosylation Analysis of Engineered H3N2 Influenza A Virus Hemagglutinins with Sequentially Added Historically Relevant Glycosylation Sites. *J. Proteome Res.* **14**, 3957–3969 (2015).
5. Lee, C.-C. D. *et al.* A cross-neutralizing antibody between HIV-1 and influenza virus. *PLoS Pathog.* **17**, e1009407 (2021).
6. Torrents de la Peña, A. *et al.* Similarities and differences between native HIV-1 envelope glycoprotein trimers and stabilized soluble trimer mimetics. *PLoS Pathog.* **15**, e1007920 (2019).
7. Sanders, R. W. *et al.* A next-generation cleaved, soluble HIV-1 Env trimer, BG505 SOSIP.664 gp140, expresses multiple epitopes for broadly neutralizing but not non-neutralizing antibodies. *PLoS Pathog.* **9**, e1003618 (2013).
8. Dey, A. K. *et al.* N-terminal substitutions in HIV-1 gp41 reduce the expression of non-trimeric envelope glycoproteins on the virus. *Virology* **372**, 187–200 (2008).
9. de Taeye, S. W. *et al.* Immunogenicity of Stabilized HIV-1 Envelope Trimers with Reduced Exposure of Non-neutralizing Epitopes. *Cell* **163**, 1702–1715 (2015).
10. Peña, A. T. de la *et al.* Improving the Immunogenicity of Native-like HIV-1 Envelope Trimers by Hyperstabilization. *Cell Reports* **20**, 1805–1817 (2017).
11. Guenaga, J. *et al.* Structure-Guided Redesign Increases the Propensity of HIV Env To Generate Highly Stable Soluble Trimers. *J. Virol.* **90**, 2806–2817 (2015).
12. Steichen, J. M. *et al.* HIV Vaccine Design to Target Germline Precursors of Glycan-Dependent Broadly Neutralizing Antibodies. *Immunity* **45**, 483–496 (2016).
13. Guenaga, J. *et al.* Glycine Substitution at Helix-to-Coil Transitions Facilitates the Structural Determination of a Stabilized Subtype C HIV Envelope Glycoprotein. *Immunity* **46**, 792–803 (2017).
