# Supplementary material for: Triple tandem trimer immunogens for HIV-1 and influenza nucleic acid-based vaccines
Source: NPJ Vaccines. 2024 Apr 6;9:74. doi: 10.1038/s41541-024-00862-8 (PMC10998906; doi:10.1038/s41541-024-00862-8)
Supplement: Supplementary file 3 — 8TGO structure validation report [file 41541_2024_862_MOESM3_ESM.pdf]

Full wwPDB X-ray Structure Validation Report (i)

Jul 13, 2023 – 11:12 AM EDT

PDB ID	:	8TGO
Title	:	Crystal structure of the BG505 triple tandem trimer gp140 HIV-1 Env in complex with PGT124 and 35O22
Deposited on	:	2023-07-12
Resolution	:	5.75 Å(reported)

This wwPDB validation report is for manuscript review

This is a Full wwPDB X-ray Structure Validation Report.

This report is produced by the wwPDB biocuration pipeline after annotation of the structure.

We welcome your comments at *validation@mail.wwpdb.org*
A user guide is available at
https://www.wwpdb.org/validation/2017/XrayValidationReportHelp
with specific help available everywhere you see the (i) symbol.

The types of validation reports are described at
http://www.wwpdb.org/validation/2017/FAQs#types.

For Manuscript Review

The following versions of software and data (see references (i)) were used in the production of this report:

MolProbity	:	4.02b-467
Mogul	:	1.8.5 (274361), CSD as541be (2020)
Xtriage (Phenix)	:	1.13
EDS	:	2.34
Percentile statistics	:	20191225.v01 (using entries in the PDB archive December 25th 2019)
Refmac	:	5.8.0158
CCP4	:	7.0.044 (Gargrove)
Ideal geometry (proteins)	:	Engh & Huber (2001)
Ideal geometry (DNA, RNA)	:	Parkinson et al. (1996)

1 Overall quality at a glance (i)

The following experimental techniques were used to determine the structure:
X-RAY DIFFRACTION

The reported resolution of this entry is 5.75 Å.

Percentile scores (ranging between 0-100) for global validation metrics of the entry are shown in the following graphic. The table shows the number of entries on which the scores are based.

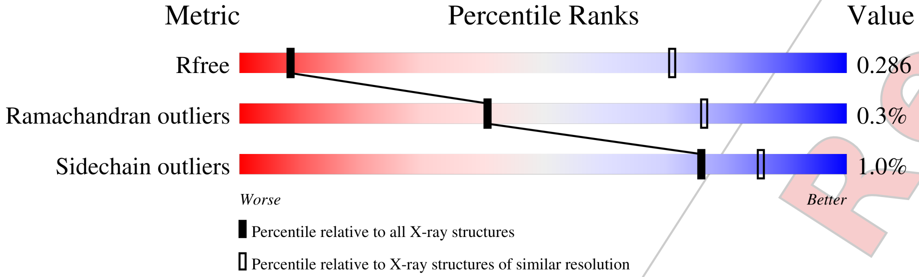

Metric	Whole archive (#Entries)	Similar resolution (#Entries, resolution range(Å))
R_{free}	130704	1007 (7.66-3.86)
Ramachandran outliers	138981	1002 (7.60-3.86)
Sidechain outliers	138945	1005 (7.70-3.82)

The table below summarises the geometric issues observed across the polymeric chains and their fit to the electron density. The red, orange, yellow and green segments of the lower bar indicate the fraction of residues that contain outliers for >=3, 2, 1 and 0 types of geometric quality criteria respectively. A grey segment represents the fraction of residues that are not modelled. The numeric value for each fraction is indicated below the corresponding segment, with a dot representing fractions <=5%

Mol	Chain	Length	Quality of chain
1	B	164	75% 25%
1	H	164	73% • 26%
1	a	164	74% • 25%
2	D	131	95% • •
2	L	131	95% • •
2	b	131	95% • •
3	C	214	96% • •

Continued on next page...

For Manuscript Review

Continued from previous page...

Mol	Chain	Length	Quality of chain
3	P	214	97% • •
3	c	214	97% • •
4	E	120	87% • • 10%
4	Q	120	85% 5% 10%
4	d	120	88% • 10%
5	G	490	89% • 10%
5	R	490	89% • 10%
5	e	490	89% 10%
6	O	235	55% 45%
6	S	235	97% • •
6	f	235	97% •
7	A	5	40% 60%
8	F	2	100%
8	K	2	100%
8	M	2	100%
8	T	2	100%
8	U	2	100%
8	W	2	100%
8	X	2	100%
8	g	2	100%
8	h	2	100%
8	j	2	100%
8	l	2	100%
8	o	2	100%
8	p	2	100%

Continued on next page...

For Manuscript Review

Continued from previous page...

Mol	Chain	Length	Quality of chain
8	r	2	100%
9	I	3	100%
9	J	3	100%
9	Y	3	100%
9	Z	3	100%
9	m	3	100%
9	n	3	100%
10	N	10	10% 90%
10	i	10	10% 90%
10	q	10	10% 90%
11	V	4	50% 50%
11	k	4	75% 25%

For Manuscript Review

2 Entry composition (i)

There are 12 unique types of molecules in this entry. The entry contains 29645 atoms, of which 0 are hydrogens and 0 are deuteriums.

In the tables below, the ZeroOcc column contains the number of atoms modelled with zero occupancy, the AltConf column contains the number of residues with at least one atom in alternate conformation and the Trace column contains the number of residues modelled with at most 2 atoms.

- Molecule 1 is a protein called Envelope glycoprotein gp41.

Mol	Chain	Residues	Atoms					ZeroOcc	AltConf	Trace
1	B	123	Total 967	C 609	N 167	O 185	S 6	0	0	0
1	H	122	Total 963	C 607	N 166	O 184	S 6	0	0	0
1	a	123	Total 967	C 609	N 167	O 185	S 6	0	0	0

There are 51 discrepancies between the modelled and reference sequences:

Chain	Residue	Modelled	Actual	Comment	Reference
B	519	SER	PHE	conflict	UNP Q2N0S6
B	559	PRO	ILE	conflict	UNP Q2N0S6
B	568	ASP	LEU	conflict	UNP Q2N0S6
B	570	HIS	VAL	conflict	UNP Q2N0S6
B	585	HIS	ARG	conflict	UNP Q2N0S6
B	605	CYS	THR	conflict	UNP Q2N0S6
B	665	GLY	-	expression tag	UNP Q2N0S6
B	666	SER	-	expression tag	UNP Q2N0S6
B	667	GLY	-	expression tag	UNP Q2N0S6
B	668	GLY	-	expression tag	UNP Q2N0S6
B	669	SER	-	expression tag	UNP Q2N0S6
B	670	GLY	-	expression tag	UNP Q2N0S6
B	671	GLY	-	expression tag	UNP Q2N0S6
B	672	SER	-	expression tag	UNP Q2N0S6
B	673	GLY	-	expression tag	UNP Q2N0S6
B	674	SER	-	expression tag	UNP Q2N0S6
B	675	GLY	-	expression tag	UNP Q2N0S6
H	519	SER	PHE	conflict	UNP Q2N0S6
H	559	PRO	ILE	conflict	UNP Q2N0S6
H	568	ASP	LEU	conflict	UNP Q2N0S6
H	570	HIS	VAL	conflict	UNP Q2N0S6
H	585	HIS	ARG	conflict	UNP Q2N0S6
H	605	CYS	THR	conflict	UNP Q2N0S6

Continued on next page...

For Manuscript Review

Continued from previous page...

Chain	Residue	Modelled	Actual	Comment	Reference
H	665	GLY	-	expression tag	UNP Q2N0S6
H	666	SER	-	expression tag	UNP Q2N0S6
H	667	GLY	-	expression tag	UNP Q2N0S6
H	668	GLY	-	expression tag	UNP Q2N0S6
H	669	SER	-	expression tag	UNP Q2N0S6
H	670	GLY	-	expression tag	UNP Q2N0S6
H	671	GLY	-	expression tag	UNP Q2N0S6
H	672	SER	-	expression tag	UNP Q2N0S6
H	673	GLY	-	expression tag	UNP Q2N0S6
H	674	SER	-	expression tag	UNP Q2N0S6
H	675	GLY	-	expression tag	UNP Q2N0S6
a	519	SER	PHE	conflict	UNP Q2N0S6
a	559	PRO	ILE	conflict	UNP Q2N0S6
a	568	ASP	LEU	conflict	UNP Q2N0S6
a	570	HIS	VAL	conflict	UNP Q2N0S6
a	585	HIS	ARG	conflict	UNP Q2N0S6
a	605	CYS	THR	conflict	UNP Q2N0S6
a	665	GLY	-	expression tag	UNP Q2N0S6
a	666	SER	-	expression tag	UNP Q2N0S6
a	667	GLY	-	expression tag	UNP Q2N0S6
a	668	GLY	-	expression tag	UNP Q2N0S6
a	669	SER	-	expression tag	UNP Q2N0S6
a	670	GLY	-	expression tag	UNP Q2N0S6
a	671	GLY	-	expression tag	UNP Q2N0S6
a	672	SER	-	expression tag	UNP Q2N0S6
a	673	GLY	-	expression tag	UNP Q2N0S6
a	674	SER	-	expression tag	UNP Q2N0S6
a	675	GLY	-	expression tag	UNP Q2N0S6

- Molecule 2 is a protein called 35O22 scFv heavy chain.

Mol	Chain	Residues	Atoms					ZeroOcc	AltConf	Trace
2	D	128	Total 994	C 628	N 169	O 192	S 5	0	0	0
2	L	128	Total 994	C 628	N 169	O 192	S 5	0	0	0
2	b	128	Total 994	C 628	N 169	O 192	S 5	0	0	0

- Molecule 3 is a protein called PGT124 light chain.

For Manuscript Review

Mol	Chain	Residues	Atoms					ZeroOcc	AltConf	Trace
3	C	208	Total 1580	C 997	N 268	O 310	S 5	0	0	0
3	P	210	Total 1595	C 1005	N 270	O 315	S 5	0	0	0
3	c	210	Total 1595	C 1005	N 270	O 315	S 5	0	0	0

- Molecule 4 is a protein called 35O22 scFv light chain.

Mol	Chain	Residues	Atoms					ZeroOcc	AltConf	Trace
4	E	108	Total 826	C 520	N 136	O 164	S 6	0	0	0
4	Q	108	Total 826	C 520	N 136	O 164	S 6	0	0	0
4	d	108	Total 826	C 520	N 136	O 164	S 6	0	0	0

- Molecule 5 is a protein called Envelope glycoprotein gp120.

Mol	Chain	Residues	Atoms					ZeroOcc	AltConf	Trace
5	G	439	Total 3473	C 2190	N 610	O 646	S 27	0	0	0
5	R	439	Total 3473	C 2190	N 610	O 646	S 27	0	0	0
5	e	439	Total 3473	C 2190	N 610	O 646	S 27	0	0	0

There are 75 discrepancies between the modelled and reference sequences:

Chain	Residue	Modelled	Actual	Comment	Reference
G	64	LYS	GLU	conflict	UNP Q2N0S6
G	106	GLU	THR	conflict	UNP Q2N0S6
G	271	ILE	MET	conflict	UNP Q2N0S6
G	288	LEU	PHE	conflict	UNP Q2N0S6
G	304	VAL	ARG	conflict	UNP Q2N0S6
G	316	TRP	ALA	conflict	UNP Q2N0S6
G	319	TYR	ALA	conflict	UNP Q2N0S6
G	332	ASN	THR	conflict	UNP Q2N0S6
G	500	LYS	ARG	conflict	UNP Q2N0S6
G	501	CYS	ALA	conflict	UNP Q2N0S6
G	508	GLY	-	expression tag	UNP Q2N0S6
G	509	GLY	-	expression tag	UNP Q2N0S6
G	510	SER	-	expression tag	UNP Q2N0S6

Continued on next page...

For Manuscript Review

Continued from previous page...

Chain	Residue	Modelled	Actual	Comment	Reference
G	511	GLY	-	expression tag	UNP Q2N0S6
G	512	GLY	-	expression tag	UNP Q2N0S6
G	513	GLY	-	expression tag	UNP Q2N0S6
G	514	GLY	-	expression tag	UNP Q2N0S6
G	515	SER	-	expression tag	UNP Q2N0S6
G	516	GLY	-	expression tag	UNP Q2N0S6
G	517	GLY	-	expression tag	UNP Q2N0S6
G	518	GLY	-	expression tag	UNP Q2N0S6
G	519	GLY	-	expression tag	UNP Q2N0S6
G	520	SER	-	expression tag	UNP Q2N0S6
G	521	GLY	-	expression tag	UNP Q2N0S6
G	522	GLY	-	expression tag	UNP Q2N0S6
R	64	LYS	GLU	conflict	UNP Q2N0S6
R	106	GLU	THR	conflict	UNP Q2N0S6
R	271	ILE	MET	conflict	UNP Q2N0S6
R	288	LEU	PHE	conflict	UNP Q2N0S6
R	304	VAL	ARG	conflict	UNP Q2N0S6
R	316	TRP	ALA	conflict	UNP Q2N0S6
R	319	TYR	ALA	conflict	UNP Q2N0S6
R	332	ASN	THR	conflict	UNP Q2N0S6
R	500	LYS	ARG	conflict	UNP Q2N0S6
R	501	CYS	ALA	conflict	UNP Q2N0S6
R	508	GLY	-	expression tag	UNP Q2N0S6
R	509	GLY	-	expression tag	UNP Q2N0S6
R	510	SER	-	expression tag	UNP Q2N0S6
R	511	GLY	-	expression tag	UNP Q2N0S6
R	512	GLY	-	expression tag	UNP Q2N0S6
R	513	GLY	-	expression tag	UNP Q2N0S6
R	514	GLY	-	expression tag	UNP Q2N0S6
R	515	SER	-	expression tag	UNP Q2N0S6
R	516	GLY	-	expression tag	UNP Q2N0S6
R	517	GLY	-	expression tag	UNP Q2N0S6
R	518	GLY	-	expression tag	UNP Q2N0S6
R	519	GLY	-	expression tag	UNP Q2N0S6
R	520	SER	-	expression tag	UNP Q2N0S6
R	521	GLY	-	expression tag	UNP Q2N0S6
R	522	GLY	-	expression tag	UNP Q2N0S6
e	64	LYS	GLU	conflict	UNP Q2N0S6
e	106	GLU	THR	conflict	UNP Q2N0S6
e	271	ILE	MET	conflict	UNP Q2N0S6
e	288	LEU	PHE	conflict	UNP Q2N0S6
e	304	VAL	ARG	conflict	UNP Q2N0S6

Continued on next page...

For Manuscript Review

Continued from previous page...

Chain	Residue	Modelled	Actual	Comment	Reference
e	316	TRP	ALA	conflict	UNP Q2N0S6
e	319	TYR	ALA	conflict	UNP Q2N0S6
e	332	ASN	THR	conflict	UNP Q2N0S6
e	500	LYS	ARG	conflict	UNP Q2N0S6
e	501	CYS	ALA	conflict	UNP Q2N0S6
e	508	GLY	-	expression tag	UNP Q2N0S6
e	509	GLY	-	expression tag	UNP Q2N0S6
e	510	SER	-	expression tag	UNP Q2N0S6
e	511	GLY	-	expression tag	UNP Q2N0S6
e	512	GLY	-	expression tag	UNP Q2N0S6
e	513	GLY	-	expression tag	UNP Q2N0S6
e	514	GLY	-	expression tag	UNP Q2N0S6
e	515	SER	-	expression tag	UNP Q2N0S6
e	516	GLY	-	expression tag	UNP Q2N0S6
e	517	GLY	-	expression tag	UNP Q2N0S6
e	518	GLY	-	expression tag	UNP Q2N0S6
e	519	GLY	-	expression tag	UNP Q2N0S6
e	520	SER	-	expression tag	UNP Q2N0S6
e	521	GLY	-	expression tag	UNP Q2N0S6
e	522	GLY	-	expression tag	UNP Q2N0S6

- Molecule 6 is a protein called PGT124 heavy chain.

Mol	Chain	Residues	Atoms					ZeroOcc	AltConf	Trace
			Total	C	N	O	S			
6	O	130	1030	655	175	197	3	0	0	0
			Total	C	N	O	S			
6	S	230	1750	1109	292	344	5	0	0	0
			Total	C	N	O	S			
6	f	230	1750	1109	292	344	5	0	0	0

- Molecule 7 is an oligosaccharide called alpha-D-mannopyranose-(1-3)-[alpha-D-mannopyranose-(1-6)]beta-D-mannopyranose-(1-4)-2-acetamido-2-deoxy-beta-D-glucopyranose-(1-4)-2-acetamido-2-deoxy-beta-D-glucopyranose.

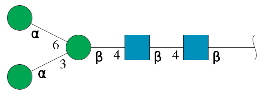

Mol	Chain	Residues	Atoms				ZeroOcc	AltConf	Trace
			Total	C	N	O			
7	A	5	61	34	2	25	0	0	0

For Manuscript Review

- Molecule 8 is an oligosaccharide called 2-acetamido-2-deoxy-beta-D-glucopyranose-(1-4)-2-acetamido-2-deoxy-beta-D-glucopyranose.

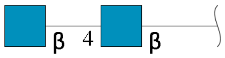

Mol	Chain	Residues	Atoms	ZeroOcc	AltConf	Trace
8	F	2	Total C N O<br>28 16 2 10	0	0	0
8	K	2	Total C N O<br>28 16 2 10	0	0	0
8	M	2	Total C N O<br>28 16 2 10	0	0	0
8	T	2	Total C N O<br>28 16 2 10	0	0	0
8	U	2	Total C N O<br>28 16 2 10	0	0	0
8	W	2	Total C N O<br>28 16 2 10	0	0	0
8	X	2	Total C N O<br>28 16 2 10	0	0	0
8	g	2	Total C N O<br>28 16 2 10	0	0	0
8	h	2	Total C N O<br>28 16 2 10	0	0	0
8	j	2	Total C N O<br>28 16 2 10	0	0	0
8	l	2	Total C N O<br>28 16 2 10	0	0	0
8	o	2	Total C N O<br>28 16 2 10	0	0	0
8	p	2	Total C N O<br>28 16 2 10	0	0	0
8	r	2	Total C N O<br>28 16 2 10	0	0	0

- Molecule 9 is an oligosaccharide called beta-D-mannopyranose-(1-4)-2-acetamido-2-deoxy-beta-D-glucopyranose-(1-4)-2-acetamido-2-deoxy-beta-D-glucopyranose.

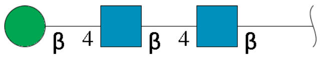

For Manuscript Review

Mol	Chain	Residues	Atoms				ZeroOcc	AltConf	Trace
			Total	C	N	O			
9	I	3	39	22	2	15	0	0	0
9	J	3	39	22	2	15	0	0	0
9	Y	3	39	22	2	15	0	0	0
9	Z	3	39	22	2	15	0	0	0
9	m	3	39	22	2	15	0	0	0
9	n	3	39	22	2	15	0	0	0

- Molecule 10 is an oligosaccharide called alpha-D-mannopyranose-(1-2)-alpha-D-mannopyranose-(1-2)-alpha-D-mannopyranose-(1-3)-[alpha-D-mannopyranose-(1-2)-alpha-D-mannopyranose-(1-6)-[alpha-D-mannopyranose-(1-3)]alpha-D-mannopyranose-(1-6)]beta-D-mannopyranose-(1-4)-2-acetamido-2-deoxy-beta-D-glucopyranose-(1-4)-2-acetamido-2-deoxy-beta-D-glucopyranose.

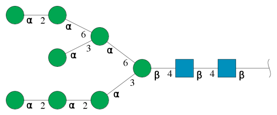

Mol	Chain	Residues	Atoms				ZeroOcc	AltConf	Trace
			Total	C	N	O			
10	N	10	116	64	2	50	0	0	0
10	i	10	116	64	2	50	0	0	0
10	q	10	116	64	2	50	0	0	0

- Molecule 11 is an oligosaccharide called alpha-D-mannopyranose-(1-3)-beta-D-mannopyranose-(1-4)-2-acetamido-2-deoxy-beta-D-glucopyranose-(1-4)-2-acetamido-2-deoxy-beta-D-glucopyranose.

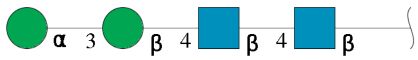

Mol	Chain	Residues	Atoms				ZeroOcc	AltConf	Trace
			Total	C	N	O			
11	V	4	50	28	2	20	0	0	0

Continued on next page...

For Manuscript Review

Continued from previous page...

Mol	Chain	Residues	Atoms				ZeroOcc	AltConf	Trace
			Total	C	N	O			
11	k	4	50	28	2	20	0	0	0

- Molecule 12 is 2-acetamido-2-deoxy-beta-D-glucopyranose (three-letter code: NAG) (formula: $C_8H_{15}NO_6$).

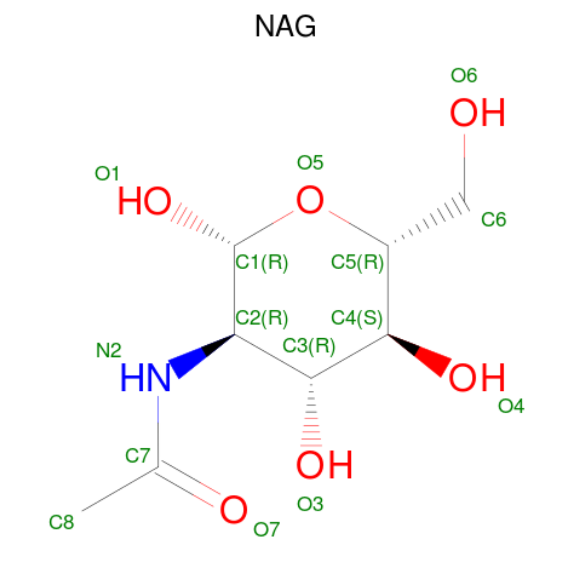

Mol	Chain	Residues	Atoms				ZeroOcc	AltConf
			Total	C	N	O		
12	B	1	14	8	1	5	0	0
12	B	1	14	8	1	5	0	0
12	B	1	14	8	1	5	0	0
12	G	1	14	8	1	5	0	0
12	G	1	14	8	1	5	0	0
12	G	1	14	8	1	5	0	0
12	G	1	14	8	1	5	0	0
12	G	1	14	8	1	5	0	0
12	G	1	14	8	1	5	0	0
12	G	1	14	8	1	5	0	0

Continued on next page...

For Manuscript Review

Continued from previous page...

Mol	Chain	Residues	Atoms	ZeroOcc	AltConf
12	G	1	Total C N O<br>14 8 1 5	0	0
12	H	1	Total C N O<br>14 8 1 5	0	0
12	H	1	Total C N O<br>14 8 1 5	0	0
12	H	1	Total C N O<br>14 8 1 5	0	0
12	R	1	Total C N O<br>14 8 1 5	0	0
12	R	1	Total C N O<br>14 8 1 5	0	0
12	R	1	Total C N O<br>14 8 1 5	0	0
12	R	1	Total C N O<br>14 8 1 5	0	0
12	R	1	Total C N O<br>14 8 1 5	0	0
12	R	1	Total C N O<br>14 8 1 5	0	0
12	a	1	Total C N O<br>14 8 1 5	0	0
12	a	1	Total C N O<br>14 8 1 5	0	0
12	a	1	Total C N O<br>14 8 1 5	0	0
12	e	1	Total C N O<br>14 8 1 5	0	0
12	e	1	Total C N O<br>14 8 1 5	0	0
12	e	1	Total C N O<br>14 8 1 5	0	0
12	e	1	Total C N O<br>14 8 1 5	0	0
12	e	1	Total C N O<br>14 8 1 5	0	0
12	e	1	Total C N O<br>14 8 1 5	0	0
12	e	1	Total C N O<br>14 8 1 5	0	0
12	e	1	Total C N O<br>14 8 1 5	0	0

For Manuscript Review

3 Residue-property plots (i)

These plots are drawn for all protein, RNA, DNA and oligosaccharide chains in the entry. The first graphic for a chain summarises the proportions of the various outlier classes displayed in the second graphic. The second graphic shows the sequence view annotated by issues in geometry. Residues are color-coded according to the number of geometric quality criteria for which they contain at least one outlier: green = 0, yellow = 1, orange = 2 and red = 3 or more. Stretches of 2 or more consecutive residues without any outlier are shown as a green connector. Residues present in the sample, but not in the model, are shown in grey.

- Molecule 1: Envelope glycoprotein gp41

Chain B:

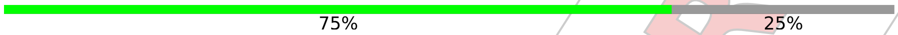

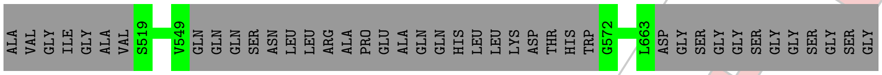

- Molecule 1: Envelope glycoprotein gp41

Chain H:

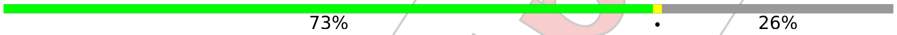

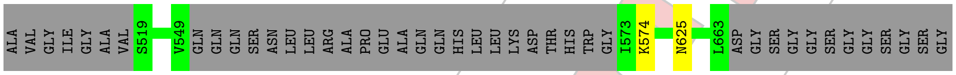

- Molecule 1: Envelope glycoprotein gp41

Chain a:

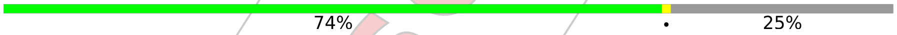

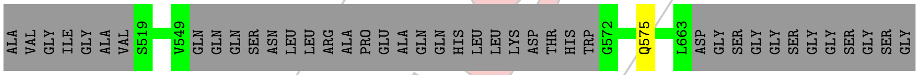

- Molecule 2: 35O22 scFv heavy chain

Chain D:

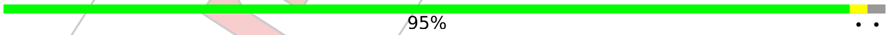

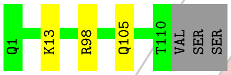

- Molecule 2: 35O22 scFv heavy chain

Chain L:

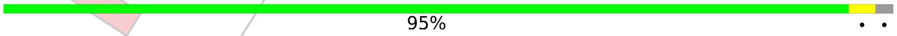

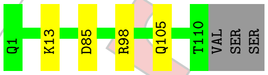

- Molecule 2: 35O22 scFv heavy chain

Chain b:

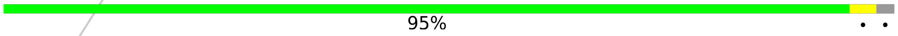

For Manuscript Review

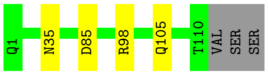

• Molecule 3: PGT124 light chain

Chain C:

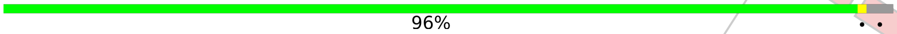

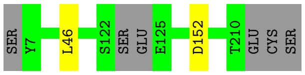

• Molecule 3: PGT124 light chain

Chain P:

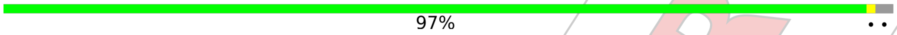

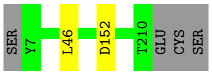

• Molecule 3: PGT124 light chain

Chain c:

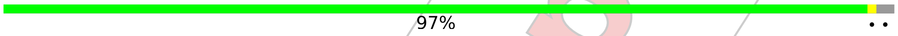

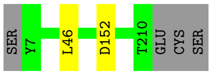

• Molecule 4: 35O22 scFv light chain

Chain E:

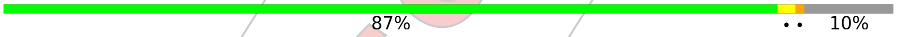

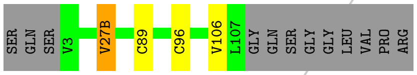

• Molecule 4: 35O22 scFv light chain

Chain Q:

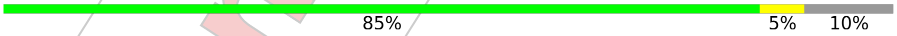

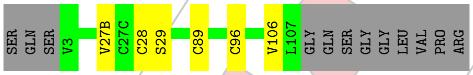

• Molecule 4: 35O22 scFv light chain

Chain d:

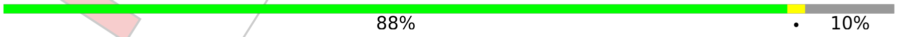

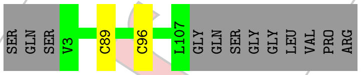

• Molecule 5: Envelope glycoprotein gp120

Chain G:

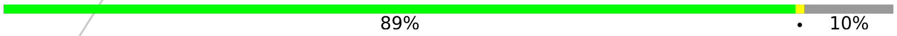

For Manuscript Review

ALA E32 C54 A60 TYR GLU THR LYS K65 P79 N185 GLU ASN GLN GLY ASN ARG SER ASN ASN SER N188 T198 Y217 K227 N398 THR SER VAL GLN GLY SER ASN SER THR GLY SER ASN D412 G458 GLY SER THR ASN SER THR T465 R504 VAL VAL

GLY GLY GLY SER GLY GLY GLY GLY SER GLY GLY GLY GLY SER GLY GLY

• Molecule 5: Envelope glycoprotein gp120

Chain R: 89% • 10%

ALA E32 C54 A60 TYR GLU THR LYS K65 I138 N185 GLU ASN GLN GLY ASN ARG SER ASN ASN SER N188 N398 THR SER VAL GLN GLY SER ASN SER THR GLY SER ASN D412 R419 G458 GLY SER THR ASN SER THR T465 R504 VAL VAL GLY GLY GLY SER

GLY GLY GLY GLY SER GLY GLY GLY GLY SER GLY GLY

• Molecule 5: Envelope glycoprotein gp120

Chain e: 89% 10%

ALA E32 C54 A60 TYR GLU THR LYS K65 N185 GLU ASN GLN GLY ASN ARG SER ASN ASN SER N188 Y217 N398 THR SER VAL GLN GLY SER ASN SER THR GLY SER ASN D412 G458 GLY SER THR ASN SER THR T465 R504 VAL VAL GLY GLY GLY SER GLY GLY

GLY GLY SER GLY GLY GLY GLY SER GLY GLY

• Molecule 6: PGT124 heavy chain

Chain O: 55% 45%

Q1 V109 SER SER ALA SER THR LYS GLY PRO SER VAL PHE PRO LEU ALA PRO SER SER LYS SER THR SER GLY GLY THR ALA ALA LEU GLY CYS LEU VAL LYS ASP TYR PHE PRO GLU PRO VAL THR VAL SER TRP ASN SER GLY ALA LEU THR SER GLY VAL HIS THR PHE PRO ALA

VAL LEU GLN SER SER GLY LEU TYR SER LEU SER SER VAL VAL THR VAL PRO SER SER SER LEU GLY THR GLN THR TYR ILE CYS ASN VAL ASN HIS LYS PRO SER ASN THR LYS VAL ASP LYS LYS VAL GLU PRO LYS SER CYS

• Molecule 6: PGT124 heavy chain

Chain S: 97% • •

Q1 S126 LYS S128 L187 GLY T189 Q190 P211 LYS SER CYS

• Molecule 6: PGT124 heavy chain

Chain f: 97% •

Q1 S126 LYS S128 G131 L187 GLY T189 P211 LYS SER CYS

For Manuscript Review

• Molecule 7: alpha-D-mannopyranose-(1-3)-[alpha-D-mannopyranose-(1-6)]beta-D-mannopyranose-(1-4)-2-acetamido-2-deoxy-beta-D-glucopyranose-(1-4)-2-acetamido-2-deoxy-beta-D-glucopyranose

Chain A:
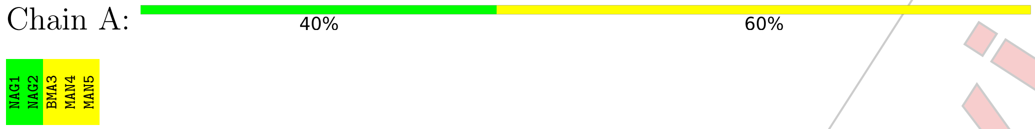

• Molecule 8: 2-acetamido-2-deoxy-beta-D-glucopyranose-(1-4)-2-acetamido-2-deoxy-beta-D-glucopyranose

Chain F:
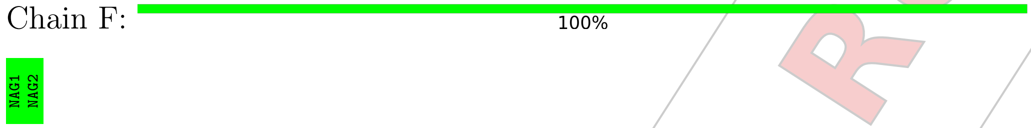

• Molecule 8: 2-acetamido-2-deoxy-beta-D-glucopyranose-(1-4)-2-acetamido-2-deoxy-beta-D-glucopyranose

Chain K:
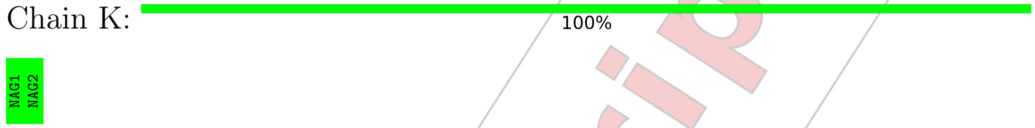

• Molecule 8: 2-acetamido-2-deoxy-beta-D-glucopyranose-(1-4)-2-acetamido-2-deoxy-beta-D-glucopyranose

Chain M:
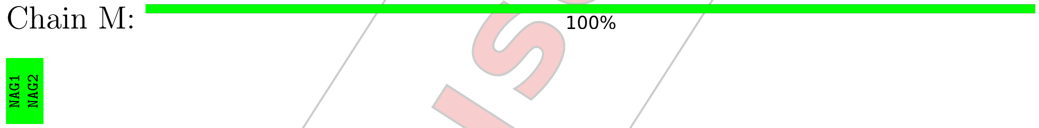

• Molecule 8: 2-acetamido-2-deoxy-beta-D-glucopyranose-(1-4)-2-acetamido-2-deoxy-beta-D-glucopyranose

Chain T:
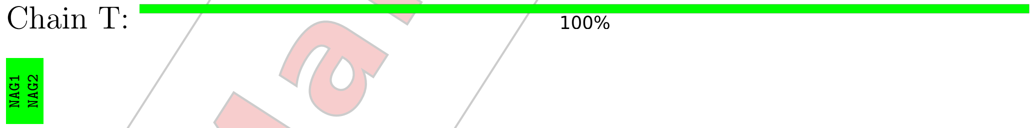

• Molecule 8: 2-acetamido-2-deoxy-beta-D-glucopyranose-(1-4)-2-acetamido-2-deoxy-beta-D-glucopyranose

Chain U:
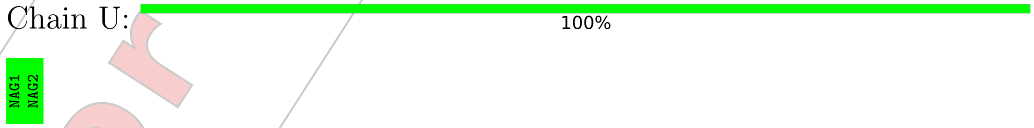

• Molecule 8: 2-acetamido-2-deoxy-beta-D-glucopyranose-(1-4)-2-acetamido-2-deoxy-beta-D-glucopyranose

For Manuscript Review

Chain W: 100%

NAG1 NAG2

• Molecule 8: 2-acetamido-2-deoxy-beta-D-glucopyranose-(1-4)-2-acetamido-2-deoxy-beta-D-glucopyranose

Chain X: 100%

NAG1 NAG2

• Molecule 8: 2-acetamido-2-deoxy-beta-D-glucopyranose-(1-4)-2-acetamido-2-deoxy-beta-D-glucopyranose

Chain g: 100%

NAG1 NAG2

• Molecule 8: 2-acetamido-2-deoxy-beta-D-glucopyranose-(1-4)-2-acetamido-2-deoxy-beta-D-glucopyranose

Chain h: 100%

NAG1 NAG2

• Molecule 8: 2-acetamido-2-deoxy-beta-D-glucopyranose-(1-4)-2-acetamido-2-deoxy-beta-D-glucopyranose

Chain j: 100%

NAG1 NAG2

• Molecule 8: 2-acetamido-2-deoxy-beta-D-glucopyranose-(1-4)-2-acetamido-2-deoxy-beta-D-glucopyranose

Chain l: 100%

NAG1 NAG2

• Molecule 8: 2-acetamido-2-deoxy-beta-D-glucopyranose-(1-4)-2-acetamido-2-deoxy-beta-D-glucopyranose

Chain o: 100%

NAG1 NAG2

For Manuscript Review

For Manuscript Review

• Molecule 8: 2-acetamido-2-deoxy-beta-D-glucopyranose-(1-4)-2-acetamido-2-deoxy-beta-D-gluc opyranose

Chain p: 100%

NAG1
NAG2

• Molecule 8: 2-acetamido-2-deoxy-beta-D-glucopyranose-(1-4)-2-acetamido-2-deoxy-beta-D-gluc opyranose

Chain r: 100%

NAG1
NAG2

• Molecule 9: beta-D-mannopyranose-(1-4)-2-acetamido-2-deoxy-beta-D-glucopyranose-(1-4)-2-ac etamido-2-deoxy-beta-D-glucopyranose

Chain I: 100%

NAG1
NAG2
BMA3

• Molecule 9: beta-D-mannopyranose-(1-4)-2-acetamido-2-deoxy-beta-D-glucopyranose-(1-4)-2-ac etamido-2-deoxy-beta-D-glucopyranose

Chain J: 100%

NAG1
NAG2
BMA3

• Molecule 9: beta-D-mannopyranose-(1-4)-2-acetamido-2-deoxy-beta-D-glucopyranose-(1-4)-2-ac etamido-2-deoxy-beta-D-glucopyranose

Chain Y: 100%

NAG1
NAG2
BMA3

• Molecule 9: beta-D-mannopyranose-(1-4)-2-acetamido-2-deoxy-beta-D-glucopyranose-(1-4)-2-ac etamido-2-deoxy-beta-D-glucopyranose

Chain Z: 100%

NAG1
NAG2
BMA3

• Molecule 9: beta-D-mannopyranose-(1-4)-2-acetamido-2-deoxy-beta-D-glucopyranose-(1-4)-2-ac etamido-2-deoxy-beta-D-glucopyranose

Chain m: 100%

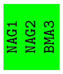

• Molecule 9: beta-D-mannopyranose-(1-4)-2-acetamido-2-deoxy-beta-D-glucopyranose-(1-4)-2-acetamido-2-deoxy-beta-D-glucopyranose

Chain n:

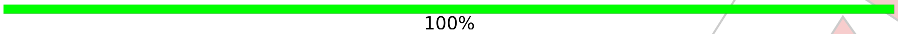

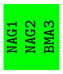

• Molecule 10: alpha-D-mannopyranose-(1-2)-alpha-D-mannopyranose-(1-2)-alpha-D-mannopyranose-(1-3)-[alpha-D-mannopyranose-(1-2)-alpha-D-mannopyranose-(1-6)-[alpha-D-mannopyranose-(1-3)]alpha-D-mannopyranose-(1-6)]beta-D-mannopyranose-(1-4)-2-acetamido-2-deoxy-beta-D-glucopyranose-(1-4)-2-acetamido-2-deoxy-beta-D-glucopyranose

Chain N:

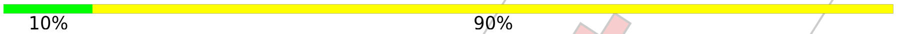

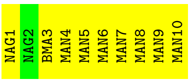

• Molecule 10: alpha-D-mannopyranose-(1-2)-alpha-D-mannopyranose-(1-2)-alpha-D-mannopyranose-(1-3)-[alpha-D-mannopyranose-(1-2)-alpha-D-mannopyranose-(1-6)-[alpha-D-mannopyranose-(1-3)]alpha-D-mannopyranose-(1-6)]beta-D-mannopyranose-(1-4)-2-acetamido-2-deoxy-beta-D-glucopyranose-(1-4)-2-acetamido-2-deoxy-beta-D-glucopyranose

Chain i:

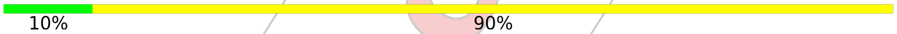

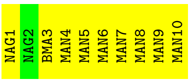

• Molecule 10: alpha-D-mannopyranose-(1-2)-alpha-D-mannopyranose-(1-2)-alpha-D-mannopyranose-(1-3)-[alpha-D-mannopyranose-(1-2)-alpha-D-mannopyranose-(1-6)-[alpha-D-mannopyranose-(1-3)]alpha-D-mannopyranose-(1-6)]beta-D-mannopyranose-(1-4)-2-acetamido-2-deoxy-beta-D-glucopyranose-(1-4)-2-acetamido-2-deoxy-beta-D-glucopyranose

Chain q:

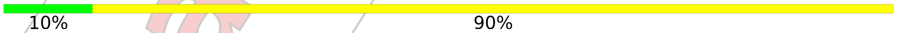

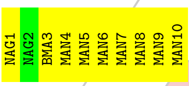

• Molecule 11: alpha-D-mannopyranose-(1-3)-beta-D-mannopyranose-(1-4)-2-acetamido-2-deoxy-beta-D-glucopyranose-(1-4)-2-acetamido-2-deoxy-beta-D-glucopyranose

Chain V:

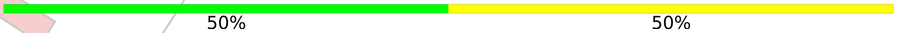

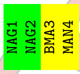

For Manuscript Review

• Molecule 11: alpha-D-mannopyranose-(1-3)-beta-D-mannopyranose-(1-4)-2-acetamido-2-deoxy-beta-D-glucopyranose-(1-4)-2-acetamido-2-deoxy-beta-D-glucopyranose

Chain k: 75% 25%

NAG1 NAG2 BMA3 MAN4

For Manuscript Review

4 Data and refinement statistics (i)

Property	Value	Source
Space group	C 1 2 1	Depositor
Cell constants<br>a, b, c, α, β, γ	357.59Å 212.04Å 207.71Å<br>90.00° 125.07° 90.00°	Depositor
Resolution (Å)	41.08 – 5.75<br>41.08 – 5.75	Depositor<br>EDS
% Data completeness<br>(in resolution range)	92.3 (41.08-5.75)<br>92.3 (41.08-5.75)	Depositor<br>EDS
R_{merge}	0.22	Depositor
R_{sym}	(Not available)	Depositor
$< I/\sigma(I) >$ [1]	2.57 (at 5.72Å)	Xtriage
Refinement program	PHENIX (1.19.2_4158: ???)	Depositor
R, R_{free}	0.242 , 0.292<br>0.239 , 0.286	Depositor<br>DCC
R_{free} test set	1551 reflections (4.64%)	wwPDB-VP
Wilson B-factor (Å^2)	213.2	Xtriage
Anisotropy	0.909	Xtriage
Bulk solvent k_{sol}(e/Å^3), B_{sol}(Å^2)	0.24 , 414.1	EDS
L-test for twinning[2]	$< \|L\| > = 0.38$, $< L^2 > = 0.21$	Xtriage
Estimated twinning fraction	0.039 for -1/2*h+3/2*k-l,1/2*h+1/2*k+l,1/2*h-1/2*k<br>0.047 for 1/2*h-1/2*k+2*l,-1/2*h+1/2*k,-1/2*h-1/2*k-l<br>0.034 for -h-k-l,l,k<br>0.049 for -h+k-l,-l,-k<br>0.035 for -1/2*h-3/2*k-l,-1/2*h+1/2*k-l,1/2*h+1/2*k<br>0.030 for 1/2*h+1/2*k+2*l,1/2*h+1/2*k,-1/2*h+1/2*k-l<br>0.048 for -1/2*h+1/2*k+l,1/2*h-1/2*k+l,1/2*h+1/2*k<br>0.035 for -1/2*h-1/2*k+l,-1/2*h-1/2*k-l,1/2*h-1/2*k<br>0.327 for 1/2*h-3/2*k,-1/2*h-1/2*k,-1/2*h+1/2*k-l<br>0.327 for 1/2*h+3/2*k,1/2*h-1/2*k,-1/2*h-1/2*k-l<br>0.038 for -h-2*l,-k,l	Xtriage
F_o,F_c correlation	0.88	EDS
Total number of atoms	29645	wwPDB-VP
Average B, all atoms (Å^2)	359.0	wwPDB-VP

[1]Intensities estimated from amplitudes.

[2]Theoretical values of $< |L| >$, $< L^2 >$ for acentric reflections are 0.5, 0.333 respectively for untwinned datasets, and 0.375, 0.2 for perfectly twinned datasets.

For Manuscript Review

Xtriage's analysis on translational NCS is as follows: *The largest off-origin peak in the Patterson function is 3.76% of the height of the origin peak. No significant pseudotranslation is detected.*

For Manuscript Review

5 Model quality (i)

5.1 Standard geometry (i)

Bond lengths and bond angles in the following residue types are not validated in this section: BMA, NAG, MAN

The Z score for a bond length (or angle) is the number of standard deviations the observed value is removed from the expected value. A bond length (or angle) with $|Z| > 5$ is considered an outlier worth inspection. RMSZ is the root-mean-square of all Z scores of the bond lengths (or angles).

Mol	Chain	Bond lengths		Bond angles	
		RMSZ	#|Z| >5	RMSZ	#|Z| >5
1	B	0.24	0/983	0.44	0/1332
1	H	0.23	0/979	0.45	0/1327
1	a	0.24	0/983	0.45	0/1332
2	D	0.25	0/1021	0.51	0/1390
2	L	0.27	0/1021	0.51	0/1390
2	b	0.26	0/1021	0.51	0/1390
3	C	0.25	0/1622	0.50	1/2215 (0.0%)
3	P	0.25	0/1638	0.51	1/2238 (0.0%)
3	c	0.25	0/1638	0.50	1/2238 (0.0%)
4	E	0.30	0/850	0.52	0/1162
4	Q	0.29	0/850	0.53	0/1162
4	d	0.25	0/850	0.50	0/1162
5	G	0.26	0/3546	0.52	0/4813
5	R	0.25	0/3546	0.51	0/4813
5	e	0.25	0/3546	0.50	0/4813
6	O	0.25	0/1055	0.52	0/1436
6	S	0.28	0/1792	0.52	0/2445
6	f	0.26	0/1792	0.52	0/2445
All	All	0.26	0/28733	0.50	3/39103 (0.0%)

There are no bond length outliers.

All (3) bond angle outliers are listed below:

Mol	Chain	Res	Type	Atoms	Z	Observed(^{o})	Ideal(^{o})
3	P	46	LEU	CA-CB-CG	5.67	128.34	115.30
3	c	46	LEU	CA-CB-CG	5.63	128.26	115.30
3	C	46	LEU	CA-CB-CG	5.36	127.63	115.30

There are no chirality outliers.

There are no planarity outliers.

For Manuscript Review

5.2 Too-close contacts (i)

Due to software issues we are unable to calculate clashes - this section is therefore empty.

5.3 Torsion angles (i)

5.3.1 Protein backbone (i)

In the following table, the Percentiles column shows the percent Ramachandran outliers of the chain as a percentile score with respect to all X-ray entries followed by that with respect to entries of similar resolution.

The Analysed column shows the number of residues for which the backbone conformation was analysed, and the total number of residues.

Mol	Chain	Analysed	Favoured	Allowed	Outliers	Percentiles	
1	B	119/164 (73%)	114 (96%)	5 (4%)	0	100	100
1	H	118/164 (72%)	112 (95%)	6 (5%)	0	100	100
1	a	119/164 (73%)	113 (95%)	6 (5%)	0	100	100
2	D	126/131 (96%)	120 (95%)	6 (5%)	0	100	100
2	L	126/131 (96%)	120 (95%)	6 (5%)	0	100	100
2	b	126/131 (96%)	121 (96%)	5 (4%)	0	100	100
3	C	204/214 (95%)	193 (95%)	10 (5%)	1 (0%)	29	69
3	P	208/214 (97%)	197 (95%)	10 (5%)	1 (0%)	29	69
3	c	208/214 (97%)	197 (95%)	10 (5%)	1 (0%)	29	69
4	E	106/120 (88%)	93 (88%)	11 (10%)	2 (2%)	8	38
4	Q	106/120 (88%)	90 (85%)	12 (11%)	4 (4%)	3	24
4	d	106/120 (88%)	93 (88%)	13 (12%)	0	100	100
5	G	429/490 (88%)	405 (94%)	23 (5%)	1 (0%)	47	81
5	R	429/490 (88%)	405 (94%)	23 (5%)	1 (0%)	47	81
5	e	429/490 (88%)	405 (94%)	24 (6%)	0	100	100
6	O	128/235 (54%)	124 (97%)	4 (3%)	0	100	100
6	S	224/235 (95%)	216 (96%)	8 (4%)	0	100	100
6	f	224/235 (95%)	215 (96%)	8 (4%)	1 (0%)	34	72
All	All	3535/4062 (87%)	3333 (94%)	190 (5%)	12 (0%)	41	76

All (12) Ramachandran outliers are listed below:

For Manuscript Review

Mol	Chain	Res	Type
4	Q	27(B)	VAL
4	Q	106	VAL
4	E	106	VAL
3	C	152	ASP
3	P	152	ASP
4	Q	28	CYS
4	Q	29	SER
3	c	152	ASP
5	R	138	ILE
6	f	131	GLY
4	E	27(B)	VAL
5	G	79	PRO

5.3.2 Protein sidechains ⓘ

In the following table, the Percentiles column shows the percent sidechain outliers of the chain as a percentile score with respect to all X-ray entries followed by that with respect to entries of similar resolution.

The Analysed column shows the number of residues for which the sidechain conformation was analysed, and the total number of residues.

Mol	Chain	Analysed	Rotameric	Outliers	Percentiles	
1	B	105/133 (79%)	105 (100%)	0	100	100
1	H	105/133 (79%)	103 (98%)	2 (2%)	57	75
1	a	105/133 (79%)	104 (99%)	1 (1%)	76	86
2	D	107/110 (97%)	104 (97%)	3 (3%)	43	64
2	L	107/110 (97%)	103 (96%)	4 (4%)	34	58
2	b	107/110 (97%)	103 (96%)	4 (4%)	34	58
3	C	174/180 (97%)	174 (100%)	0	100	100
3	P	176/180 (98%)	176 (100%)	0	100	100
3	c	176/180 (98%)	176 (100%)	0	100	100
4	E	95/104 (91%)	92 (97%)	3 (3%)	39	61
4	Q	95/104 (91%)	93 (98%)	2 (2%)	53	72
4	d	95/104 (91%)	93 (98%)	2 (2%)	53	72
5	G	394/427 (92%)	390 (99%)	4 (1%)	76	86
5	R	394/427 (92%)	392 (100%)	2 (0%)	88	93
5	e	394/427 (92%)	392 (100%)	2 (0%)	88	93

Continued on next page...

For Manuscript Review

Continued from previous page...

Mol	Chain	Analysed	Rotameric	Outliers	Percentiles	
6	O	112/203 (55%)	112 (100%)	0	100	100
6	S	199/203 (98%)	197 (99%)	2 (1%)	76	86
6	f	199/203 (98%)	199 (100%)	0	100	100
All	All	3139/3471 (90%)	3108 (99%)	31 (1%)	76	86

All (31) residues with a non-rotameric sidechain are listed below:

Mol	Chain	Res	Type
2	D	13	LYS
2	D	98	ARG
2	D	105	GLN
4	E	27(B)	VAL
4	E	89	CYS
4	E	96	CYS
5	G	54	CYS
5	G	198	THR
5	G	217	TYR
5	G	227	LYS
1	H	574	LYS
1	H	625	ASN
2	L	13	LYS
2	L	85	ASP
2	L	98	ARG
2	L	105	GLN
4	Q	89	CYS
4	Q	96	CYS
5	R	54	CYS
5	R	419	ARG
6	S	187	LEU
6	S	190	GLN
1	a	575	GLN
2	b	35	ASN
2	b	85	ASP
2	b	98	ARG
2	b	105	GLN
4	d	89	CYS
4	d	96	CYS
5	e	54	CYS
5	e	217	TYR

Sometimes sidechains can be flipped to improve hydrogen bonding and reduce clashes. All (3) such

For Manuscript Review

sidechains are listed below:

Mol	Chain	Res	Type
3	P	170	ASN
5	R	105	HIS
5	e	105	HIS

5.3.3 RNA (i)

There are no RNA molecules in this entry.

5.4 Non-standard residues in protein, DNA, RNA chains (i)

There are no non-standard protein/DNA/RNA residues in this entry.

5.5 Carbohydrates (i)

89 monosaccharides are modelled in this entry.

In the following table, the Counts columns list the number of bonds (or angles) for which Mogul statistics could be retrieved, the number of bonds (or angles) that are observed in the model and the number of bonds (or angles) that are defined in the Chemical Component Dictionary. The Link column lists molecule types, if any, to which the group is linked. The Z score for a bond length (or angle) is the number of standard deviations the observed value is removed from the expected value. A bond length (or angle) with $|Z| > 2$ is considered an outlier worth inspection. RMSZ is the root-mean-square of all Z scores of the bond lengths (or angles).

Mol	Type	Chain	Res	Link	Bond lengths			Bond angles		
					Counts	RMSZ	$\#\|Z\|>2$	Counts	RMSZ	$\#\|Z\|>2$
7	NAG	A	1	5,7	14,14,15	0.29	0	17,19,21	0.33	0
7	NAG	A	2	7	14,14,15	0.47	0	17,19,21	0.51	0
7	BMA	A	3	7	11,11,12	1.00	1 (9%)	15,15,17	0.95	1 (6%)
7	MAN	A	4	7	11,11,12	0.59	0	15,15,17	1.00	2 (13%)
7	MAN	A	5	7	11,11,12	0.92	1 (9%)	15,15,17	1.47	3 (20%)
8	NAG	F	1	2,8	14,14,15	0.17	0	17,19,21	0.40	0
8	NAG	F	2	8	14,14,15	0.29	0	17,19,21	0.55	0
9	NAG	I	1	5,9	14,14,15	0.26	0	17,19,21	0.45	0
9	NAG	I	2	9	14,14,15	0.24	0	17,19,21	0.41	0
9	BMA	I	3	9	11,11,12	0.67	0	15,15,17	0.74	0
9	NAG	J	1	5,9	14,14,15	0.27	0	17,19,21	0.48	0
9	NAG	J	2	9	14,14,15	0.20	0	17,19,21	0.42	0
9	BMA	J	3	9	11,11,12	0.66	0	15,15,17	0.78	0
8	NAG	K	1	5,8	14,14,15	0.27	0	17,19,21	0.44	0

For Manuscript Review

Mol	Type	Chain	Res	Link	Bond lengths			Bond angles		
					Counts	RMSZ	#|Z| > 2	Counts	RMSZ	#|Z| > 2
8	NAG	K	2	8	14,14,15	0.20	0	17,19,21	0.45	0
8	NAG	M	1	5,8	14,14,15	0.22	0	17,19,21	0.40	0
8	NAG	M	2	8	14,14,15	0.23	0	17,19,21	0.50	0
10	NAG	N	1	5,10	14,14,15	0.73	1 (7%)	17,19,21	1.40	2 (11%)
10	MAN	N	10	10	11,11,12	0.94	0	15,15,17	1.17	2 (13%)
10	NAG	N	2	10	14,14,15	0.24	0	17,19,21	0.41	0
10	BMA	N	3	10	11,11,12	1.04	0	15,15,17	0.99	1 (6%)
10	MAN	N	4	10	11,11,12	1.06	1 (9%)	15,15,17	1.35	2 (13%)
10	MAN	N	5	10	11,11,12	0.73	0	15,15,17	1.12	2 (13%)
10	MAN	N	6	10	11,11,12	0.78	0	15,15,17	0.98	2 (13%)
10	MAN	N	7	10	11,11,12	0.80	0	15,15,17	1.01	2 (13%)
10	MAN	N	8	10	11,11,12	0.73	0	15,15,17	1.18	2 (13%)
10	MAN	N	9	10	11,11,12	0.61	0	15,15,17	1.23	2 (13%)
8	NAG	T	1	5,8	14,14,15	0.25	0	17,19,21	0.53	0
8	NAG	T	2	8	14,14,15	0.34	0	17,19,21	0.41	0
8	NAG	U	1	5,8	14,14,15	0.21	0	17,19,21	0.48	0
8	NAG	U	2	8	14,14,15	0.26	0	17,19,21	0.45	0
11	NAG	V	1	5,11	14,14,15	0.30	0	17,19,21	0.33	0
11	NAG	V	2	11	14,14,15	0.52	0	17,19,21	0.60	0
11	BMA	V	3	11	11,11,12	0.93	1 (9%)	15,15,17	1.03	1 (6%)
11	MAN	V	4	11	11,11,12	0.70	0	15,15,17	2.78	4 (26%)
8	NAG	W	1	5,8	14,14,15	0.30	0	17,19,21	0.59	0
8	NAG	W	2	8	14,14,15	0.47	0	17,19,21	0.58	0
8	NAG	X	1	2,8	14,14,15	0.22	0	17,19,21	0.40	0
8	NAG	X	2	8	14,14,15	0.29	0	17,19,21	0.55	0
9	NAG	Y	1	5,9	14,14,15	0.22	0	17,19,21	0.46	0
9	NAG	Y	2	9	14,14,15	0.23	0	17,19,21	0.43	0
9	BMA	Y	3	9	11,11,12	0.63	0	15,15,17	0.75	0
9	NAG	Z	1	5,9	14,14,15	0.27	0	17,19,21	0.49	0
9	NAG	Z	2	9	14,14,15	0.31	0	17,19,21	0.43	0
9	BMA	Z	3	9	11,11,12	0.68	0	15,15,17	0.80	0
8	NAG	g	1	5,8	14,14,15	0.24	0	17,19,21	0.45	0
8	NAG	g	2	8	14,14,15	0.22	0	17,19,21	0.44	0
8	NAG	h	1	5,8	14,14,15	0.21	0	17,19,21	0.39	0
8	NAG	h	2	8	14,14,15	0.23	0	17,19,21	0.49	0
10	NAG	i	1	5,10	14,14,15	0.72	1 (7%)	17,19,21	1.40	2 (11%)
10	MAN	i	10	10	11,11,12	0.89	0	15,15,17	1.15	1 (6%)
10	NAG	i	2	10	14,14,15	0.25	0	17,19,21	0.41	0
10	BMA	i	3	10	11,11,12	1.15	1 (9%)	15,15,17	1.02	1 (6%)

For Manuscript Review

Mol	Type	Chain	Res	Link	Bond lengths			Bond angles		
					Counts	RMSZ	#|Z| > 2	Counts	RMSZ	#|Z| > 2
10	MAN	i	4	10	11,11,12	1.07	1 (9%)	15,15,17	1.37	2 (13%)
10	MAN	i	5	10	11,11,12	0.67	0	15,15,17	1.14	2 (13%)
10	MAN	i	6	10	11,11,12	0.78	0	15,15,17	0.97	2 (13%)
10	MAN	i	7	10	11,11,12	0.82	0	15,15,17	1.00	2 (13%)
10	MAN	i	8	10	11,11,12	0.71	0	15,15,17	1.13	2 (13%)
10	MAN	i	9	10	11,11,12	0.71	0	15,15,17	1.23	2 (13%)
8	NAG	j	1	5,8	14,14,15	0.22	0	17,19,21	0.48	0
8	NAG	j	2	8	14,14,15	0.27	0	17,19,21	0.40	0
11	NAG	k	1	5,11	14,14,15	0.43	0	17,19,21	0.36	0
11	NAG	k	2	11	14,14,15	0.31	0	17,19,21	0.58	0
11	BMA	k	3	11	11,11,12	0.68	0	15,15,17	0.76	0
11	MAN	k	4	11	11,11,12	0.76	0	15,15,17	1.57	4 (26%)
8	NAG	l	1	2,8	14,14,15	0.23	0	17,19,21	0.41	0
8	NAG	l	2	8	14,14,15	0.27	0	17,19,21	0.54	0
9	NAG	m	1	9	14,14,15	0.20	0	17,19,21	0.45	0
9	NAG	m	2	9	14,14,15	0.27	0	17,19,21	0.40	0
9	BMA	m	3	9	11,11,12	0.64	0	15,15,17	0.73	0
9	NAG	n	1	5,9	14,14,15	0.21	0	17,19,21	0.45	0
9	NAG	n	2	9	14,14,15	0.24	0	17,19,21	0.42	0
9	BMA	n	3	9	11,11,12	0.63	0	15,15,17	0.76	0
8	NAG	o	1	5,8	14,14,15	0.29	0	17,19,21	0.48	0
8	NAG	o	2	8	14,14,15	0.22	0	17,19,21	0.47	0
8	NAG	p	1	5,8	14,14,15	0.22	0	17,19,21	0.41	0
8	NAG	p	2	8	14,14,15	0.20	0	17,19,21	0.50	0
10	NAG	q	1	5,10	14,14,15	0.72	1 (7%)	17,19,21	1.43	2 (11%)
10	MAN	q	10	10	11,11,12	0.90	0	15,15,17	1.15	1 (6%)
10	NAG	q	2	10	14,14,15	0.25	0	17,19,21	0.38	0
10	BMA	q	3	10	11,11,12	0.97	0	15,15,17	0.98	1 (6%)
10	MAN	q	4	10	11,11,12	1.08	1 (9%)	15,15,17	1.36	4 (26%)
10	MAN	q	5	10	11,11,12	0.74	0	15,15,17	1.19	2 (13%)
10	MAN	q	6	10	11,11,12	0.79	0	15,15,17	0.98	2 (13%)
10	MAN	q	7	10	11,11,12	0.73	0	15,15,17	1.02	2 (13%)
10	MAN	q	8	10	11,11,12	0.74	0	15,15,17	1.06	1 (6%)
10	MAN	q	9	10	11,11,12	0.77	0	15,15,17	1.30	2 (13%)
8	NAG	r	1	5,8	14,14,15	0.22	0	17,19,21	0.47	0
8	NAG	r	2	8	14,14,15	0.34	0	17,19,21	0.42	0

In the following table, the Chirals column lists the number of chiral outliers, the number of chiral centers analysed, the number of these observed in the model and the number defined in the

For Manuscript Review

Chemical Component Dictionary. Similar counts are reported in the Torsion and Rings columns. '-' means no outliers of that kind were identified.

Mol	Type	Chain	Res	Link	Chirals	Torsions	Rings
7	NAG	A	1	5,7	-	0/6/23/26	0/1/1/1
7	NAG	A	2	7	-	2/6/23/26	0/1/1/1
7	BMA	A	3	7	-	2/2/19/22	0/1/1/1
7	MAN	A	4	7	-	1/2/19/22	0/1/1/1
7	MAN	A	5	7	-	2/2/19/22	1/1/1/1
8	NAG	F	1	2,8	-	2/6/23/26	0/1/1/1
8	NAG	F	2	8	-	1/6/23/26	0/1/1/1
9	NAG	I	1	5,9	-	2/6/23/26	0/1/1/1
9	NAG	I	2	9	-	0/6/23/26	0/1/1/1
9	BMA	I	3	9	-	0/2/19/22	0/1/1/1
9	NAG	J	1	5,9	-	2/6/23/26	0/1/1/1
9	NAG	J	2	9	-	2/6/23/26	0/1/1/1
9	BMA	J	3	9	-	1/2/19/22	0/1/1/1
8	NAG	K	1	5,8	-	2/6/23/26	0/1/1/1
8	NAG	K	2	8	-	2/6/23/26	0/1/1/1
8	NAG	M	1	5,8	-	0/6/23/26	0/1/1/1
8	NAG	M	2	8	-	1/6/23/26	0/1/1/1
10	NAG	N	1	5,10	-	5/6/23/26	0/1/1/1
10	MAN	N	10	10	-	0/2/19/22	0/1/1/1
10	NAG	N	2	10	-	2/6/23/26	0/1/1/1
10	BMA	N	3	10	-	0/2/19/22	0/1/1/1
10	MAN	N	4	10	-	0/2/19/22	0/1/1/1
10	MAN	N	5	10	-	2/2/19/22	0/1/1/1
10	MAN	N	6	10	-	0/2/19/22	0/1/1/1
10	MAN	N	7	10	-	0/2/19/22	0/1/1/1
10	MAN	N	8	10	-	0/2/19/22	0/1/1/1
10	MAN	N	9	10	-	0/2/19/22	0/1/1/1
8	NAG	T	1	5,8	-	0/6/23/26	0/1/1/1
8	NAG	T	2	8	-	2/6/23/26	0/1/1/1
8	NAG	U	1	5,8	-	1/6/23/26	0/1/1/1
8	NAG	U	2	8	-	1/6/23/26	0/1/1/1
11	NAG	V	1	5,11	-	1/6/23/26	0/1/1/1
11	NAG	V	2	11	-	2/6/23/26	0/1/1/1
11	BMA	V	3	11	-	0/2/19/22	0/1/1/1
11	MAN	V	4	11	-	0/2/19/22	0/1/1/1
8	NAG	W	1	5,8	-	0/6/23/26	0/1/1/1
8	NAG	W	2	8	-	0/6/23/26	0/1/1/1

Continued on next page...

For Manuscript Review

Continued from previous page...

Mol	Type	Chain	Res	Link	Chirals	Torsions	Rings
8	NAG	X	1	2,8	-	2/6/23/26	0/1/1/1
8	NAG	X	2	8	-	1/6/23/26	0/1/1/1
9	NAG	Y	1	5,9	-	2/6/23/26	0/1/1/1
9	NAG	Y	2	9	-	0/6/23/26	0/1/1/1
9	BMA	Y	3	9	-	0/2/19/22	0/1/1/1
9	NAG	Z	1	5,9	-	2/6/23/26	0/1/1/1
9	NAG	Z	2	9	-	2/6/23/26	0/1/1/1
9	BMA	Z	3	9	-	1/2/19/22	0/1/1/1
8	NAG	g	1	5,8	-	2/6/23/26	0/1/1/1
8	NAG	g	2	8	-	2/6/23/26	0/1/1/1
8	NAG	h	1	5,8	-	0/6/23/26	0/1/1/1
8	NAG	h	2	8	-	1/6/23/26	0/1/1/1
10	NAG	i	1	5,10	-	5/6/23/26	0/1/1/1
10	MAN	i	10	10	-	0/2/19/22	0/1/1/1
10	NAG	i	2	10	-	2/6/23/26	0/1/1/1
10	BMA	i	3	10	-	0/2/19/22	0/1/1/1
10	MAN	i	4	10	-	0/2/19/22	0/1/1/1
10	MAN	i	5	10	-	2/2/19/22	0/1/1/1
10	MAN	i	6	10	-	0/2/19/22	0/1/1/1
10	MAN	i	7	10	-	0/2/19/22	0/1/1/1
10	MAN	i	8	10	-	0/2/19/22	0/1/1/1
10	MAN	i	9	10	-	0/2/19/22	0/1/1/1
8	NAG	j	1	5,8	-	2/6/23/26	0/1/1/1
8	NAG	j	2	8	-	0/6/23/26	0/1/1/1
11	NAG	k	1	5,11	-	1/6/23/26	0/1/1/1
11	NAG	k	2	11	-	1/6/23/26	0/1/1/1
11	BMA	k	3	11	-	0/2/19/22	0/1/1/1
11	MAN	k	4	11	-	1/2/19/22	0/1/1/1
8	NAG	l	1	2,8	-	2/6/23/26	0/1/1/1
8	NAG	l	2	8	-	3/6/23/26	0/1/1/1
9	NAG	m	1	9	-	2/6/23/26	0/1/1/1
9	NAG	m	2	9	-	1/6/23/26	0/1/1/1
9	BMA	m	3	9	-	0/2/19/22	0/1/1/1
9	NAG	n	1	5,9	-	2/6/23/26	0/1/1/1
9	NAG	n	2	9	-	0/6/23/26	0/1/1/1
9	BMA	n	3	9	-	1/2/19/22	0/1/1/1
8	NAG	o	1	5,8	-	2/6/23/26	0/1/1/1
8	NAG	o	2	8	-	2/6/23/26	0/1/1/1

Continued on next page...

For Manuscript Review

Continued from previous page...

Mol	Type	Chain	Res	Link	Chirals	Torsions	Rings
8	NAG	p	1	5,8	-	0/6/23/26	0/1/1/1
8	NAG	p	2	8	-	1/6/23/26	0/1/1/1
10	NAG	q	1	5,10	-	4/6/23/26	0/1/1/1
10	MAN	q	10	10	-	0/2/19/22	0/1/1/1
10	NAG	q	2	10	-	2/6/23/26	0/1/1/1
10	BMA	q	3	10	-	0/2/19/22	0/1/1/1
10	MAN	q	4	10	-	0/2/19/22	0/1/1/1
10	MAN	q	5	10	-	2/2/19/22	0/1/1/1
10	MAN	q	6	10	-	0/2/19/22	0/1/1/1
10	MAN	q	7	10	-	0/2/19/22	0/1/1/1
10	MAN	q	8	10	-	0/2/19/22	0/1/1/1
10	MAN	q	9	10	-	0/2/19/22	0/1/1/1
8	NAG	r	1	5,8	-	0/6/23/26	0/1/1/1
8	NAG	r	2	8	-	0/6/23/26	0/1/1/1

All (10) bond length outliers are listed below:

Mol	Chain	Res	Type	Atoms	Z	Observed(Å)	Ideal(Å)
10	N	4	MAN	C1-C2	3.10	1.59	1.52
10	i	4	MAN	C1-C2	3.10	1.59	1.52
10	q	4	MAN	C1-C2	2.96	1.58	1.52
10	N	1	NAG	O5-C1	-2.59	1.39	1.43
10	q	1	NAG	O5-C1	-2.56	1.39	1.43
10	i	1	NAG	O5-C1	-2.56	1.39	1.43
10	i	3	BMA	O5-C1	-2.39	1.39	1.43
11	V	3	BMA	C4-C3	2.17	1.57	1.52
7	A	3	BMA	C1-C2	2.14	1.57	1.52
7	A	5	MAN	C1-C2	2.07	1.56	1.52

All (65) bond angle outliers are listed below:

Mol	Chain	Res	Type	Atoms	Z	Observed(°)	Ideal(°)
11	V	4	MAN	C1-O5-C5	8.41	123.59	112.19
11	V	4	MAN	O5-C1-C2	4.85	118.26	110.77
10	q	1	NAG	C2-N2-C7	4.46	129.25	122.90
10	N	1	NAG	C2-N2-C7	4.40	129.16	122.90
10	i	1	NAG	C2-N2-C7	4.38	129.14	122.90
10	q	9	MAN	C1-O5-C5	4.12	117.77	112.19
10	N	9	MAN	C1-O5-C5	3.77	117.30	112.19
11	k	4	MAN	C1-O5-C5	3.71	117.22	112.19
10	i	9	MAN	C1-O5-C5	3.65	117.14	112.19

Continued on next page...

For Manuscript Review

Continued from previous page...

Mol	Chain	Res	Type	Atoms	Z	Observed(°)	Ideal(°)
7	A	5	MAN	C1-O5-C5	3.50	116.93	112.19
11	V	4	MAN	C1-C2-C3	3.31	113.74	109.67
10	q	5	MAN	O2-C2-C3	-3.22	103.69	110.14
10	q	8	MAN	C1-O5-C5	3.09	116.38	112.19
10	N	8	MAN	C1-O5-C5	3.08	116.37	112.19
10	i	8	MAN	C1-O5-C5	2.98	116.23	112.19
10	i	5	MAN	O2-C2-C3	-2.93	104.26	110.14
10	N	4	MAN	C1-O5-C5	2.92	116.15	112.19
10	N	5	MAN	O2-C2-C3	-2.81	104.50	110.14
10	i	4	MAN	C1-O5-C5	2.81	116.00	112.19
10	q	4	MAN	C1-O5-C5	2.68	115.83	112.19
10	q	5	MAN	C1-O5-C5	2.66	115.80	112.19
7	A	4	MAN	C1-O5-C5	2.58	115.69	112.19
10	N	5	MAN	C1-O5-C5	2.55	115.64	112.19
11	V	3	BMA	O3-C3-C4	2.53	116.20	110.35
10	i	5	MAN	C1-O5-C5	2.51	115.60	112.19
10	i	3	BMA	C1-C2-C3	2.49	112.73	109.67
11	V	4	MAN	O2-C2-C3	-2.47	105.20	110.14
10	q	3	BMA	C1-C2-C3	2.41	112.62	109.67
10	i	10	MAN	O2-C2-C3	-2.38	105.38	110.14
7	A	3	BMA	O3-C3-C2	2.37	114.54	109.99
10	q	10	MAN	O2-C2-C3	-2.35	105.43	110.14
10	N	10	MAN	O2-C2-C3	-2.34	105.44	110.14
11	k	4	MAN	O5-C5-C6	-2.34	103.54	107.20
11	k	4	MAN	O5-C1-C2	2.34	114.38	110.77
10	N	6	MAN	C1-O5-C5	2.30	115.31	112.19
10	N	1	NAG	C1-C2-N2	2.29	114.40	110.49
10	i	1	NAG	C1-C2-N2	2.28	114.39	110.49
7	A	5	MAN	O5-C5-C6	2.27	110.77	107.20
10	q	7	MAN	C1-O5-C5	2.27	115.26	112.19
10	q	6	MAN	C1-O5-C5	2.26	115.25	112.19
10	q	1	NAG	C1-C2-N2	2.24	114.32	110.49
10	N	3	BMA	C1-C2-C3	2.23	112.41	109.67
11	k	4	MAN	O2-C2-C3	-2.22	105.68	110.14
10	N	7	MAN	O2-C2-C3	-2.21	105.71	110.14
7	A	4	MAN	O2-C2-C3	-2.20	105.73	110.14
10	i	4	MAN	C1-C2-C3	2.20	112.37	109.67
10	i	6	MAN	C1-O5-C5	2.19	115.16	112.19
10	N	6	MAN	O2-C2-C3	-2.19	105.76	110.14
10	i	9	MAN	O2-C2-C3	-2.18	105.76	110.14
10	i	6	MAN	O2-C2-C3	-2.18	105.77	110.14
10	q	7	MAN	O2-C2-C3	-2.18	105.78	110.14

Continued on next page...

For Manuscript Review

Continued from previous page...

Mol	Chain	Res	Type	Atoms	Z	Observed(°)	Ideal(°)
10	q	6	MAN	O2-C2-C3	-2.18	105.78	110.14
10	N	9	MAN	O2-C2-C3	-2.17	105.80	110.14
10	q	9	MAN	O2-C2-C3	-2.17	105.80	110.14
10	q	4	MAN	O2-C2-C1	2.16	113.57	109.15
10	N	8	MAN	O3-C3-C2	2.14	114.10	109.99
10	N	7	MAN	C1-O5-C5	2.14	115.09	112.19
10	i	7	MAN	O2-C2-C3	-2.10	105.93	110.14
10	i	7	MAN	C1-O5-C5	2.09	115.03	112.19
10	N	4	MAN	C1-C2-C3	2.07	112.21	109.67
7	A	5	MAN	O2-C2-C3	-2.06	106.01	110.14
10	q	4	MAN	O5-C1-C2	2.05	113.94	110.77
10	N	10	MAN	C1-O5-C5	2.04	114.96	112.19
10	q	4	MAN	C1-C2-C3	2.04	112.17	109.67
10	i	8	MAN	O2-C2-C3	-2.02	106.10	110.14

There are no chirality outliers.

All (91) torsion outliers are listed below:

Mol	Chain	Res	Type	Atoms
9	J	1	NAG	O5-C5-C6-O6
7	A	3	BMA	O5-C5-C6-O6
8	K	1	NAG	O5-C5-C6-O6
8	g	1	NAG	O5-C5-C6-O6
7	A	5	MAN	C4-C5-C6-O6
8	K	2	NAG	O5-C5-C6-O6
9	Y	1	NAG	O5-C5-C6-O6
11	V	2	NAG	O5-C5-C6-O6
9	Z	1	NAG	O5-C5-C6-O6
11	V	2	NAG	C4-C5-C6-O6
9	J	2	NAG	O5-C5-C6-O6
9	n	1	NAG	O5-C5-C6-O6
10	N	2	NAG	O5-C5-C6-O6
10	q	2	NAG	O5-C5-C6-O6
9	Y	1	NAG	C4-C5-C6-O6
8	g	2	NAG	O5-C5-C6-O6
9	Z	1	NAG	C4-C5-C6-O6
8	o	1	NAG	O5-C5-C6-O6
8	g	1	NAG	C4-C5-C6-O6
9	J	1	NAG	C4-C5-C6-O6
10	N	2	NAG	C4-C5-C6-O6
8	o	2	NAG	O5-C5-C6-O6
7	A	3	BMA	C4-C5-C6-O6

Continued on next page...

For Manuscript Review

Continued from previous page...

Mol	Chain	Res	Type	Atoms
8	K	1	NAG	C4-C5-C6-O6
8	K	2	NAG	C4-C5-C6-O6
7	A	5	MAN	O5-C5-C6-O6
8	X	1	NAG	C4-C5-C6-O6
9	n	1	NAG	C4-C5-C6-O6
10	q	2	NAG	C4-C5-C6-O6
10	i	2	NAG	O5-C5-C6-O6
10	N	5	MAN	C4-C5-C6-O6
10	q	5	MAN	C4-C5-C6-O6
8	o	1	NAG	C4-C5-C6-O6
10	i	5	MAN	C4-C5-C6-O6
8	o	2	NAG	C4-C5-C6-O6
9	m	1	NAG	C8-C7-N2-C2
9	m	1	NAG	O7-C7-N2-C2
10	N	1	NAG	C8-C7-N2-C2
10	N	1	NAG	O7-C7-N2-C2
10	i	1	NAG	C8-C7-N2-C2
10	i	1	NAG	O7-C7-N2-C2
10	q	1	NAG	C8-C7-N2-C2
10	q	1	NAG	O7-C7-N2-C2
8	X	1	NAG	O5-C5-C6-O6
10	N	1	NAG	O5-C5-C6-O6
9	J	2	NAG	C4-C5-C6-O6
10	i	2	NAG	C4-C5-C6-O6
8	F	1	NAG	C4-C5-C6-O6
10	N	1	NAG	C4-C5-C6-O6
8	T	2	NAG	O5-C5-C6-O6
8	T	2	NAG	C4-C5-C6-O6
7	A	2	NAG	O5-C5-C6-O6
8	l	1	NAG	C4-C5-C6-O6
8	g	2	NAG	C4-C5-C6-O6
10	N	5	MAN	O5-C5-C6-O6
10	i	5	MAN	O5-C5-C6-O6
10	q	5	MAN	O5-C5-C6-O6
8	F	1	NAG	O5-C5-C6-O6
8	l	2	NAG	C4-C5-C6-O6
9	I	1	NAG	C4-C5-C6-O6
8	l	2	NAG	O5-C5-C6-O6
9	Z	3	BMA	O5-C5-C6-O6
9	n	3	BMA	O5-C5-C6-O6
7	A	4	MAN	O5-C5-C6-O6
8	l	1	NAG	O5-C5-C6-O6

Continued on next page...

For Manuscript Review

Continued from previous page...

Mol	Chain	Res	Type	Atoms
8	M	2	NAG	O5-C5-C6-O6
11	k	4	MAN	O5-C5-C6-O6
8	h	2	NAG	O5-C5-C6-O6
10	i	1	NAG	C4-C5-C6-O6
8	U	1	NAG	O5-C5-C6-O6
9	J	3	BMA	O5-C5-C6-O6
8	p	2	NAG	O5-C5-C6-O6
9	Z	2	NAG	C4-C5-C6-O6
9	I	1	NAG	O5-C5-C6-O6
9	Z	2	NAG	O5-C5-C6-O6
10	i	1	NAG	O5-C5-C6-O6
8	j	1	NAG	C4-C5-C6-O6
7	A	2	NAG	C4-C5-C6-O6
11	V	1	NAG	O5-C5-C6-O6
8	F	2	NAG	C3-C2-N2-C7
8	X	2	NAG	C3-C2-N2-C7
8	l	2	NAG	C3-C2-N2-C7
11	k	2	NAG	C3-C2-N2-C7
8	j	1	NAG	O5-C5-C6-O6
11	k	1	NAG	O5-C5-C6-O6
10	N	1	NAG	C3-C2-N2-C7
10	i	1	NAG	C3-C2-N2-C7
10	q	1	NAG	C3-C2-N2-C7
9	m	2	NAG	C4-C5-C6-O6
8	U	2	NAG	C4-C5-C6-O6
10	q	1	NAG	C4-C5-C6-O6

All (1) ring outliers are listed below:

Mol	Chain	Res	Type	Atoms
7	A	5	MAN	C1-C2-C3-C4-C5-O5

No monomer is involved in short contacts.

The following is a two-dimensional graphical depiction of Mogul quality analysis of bond lengths, bond angles, torsion angles, and ring geometry for oligosaccharide.

For Manuscript Review

Oligosaccharide Chain A

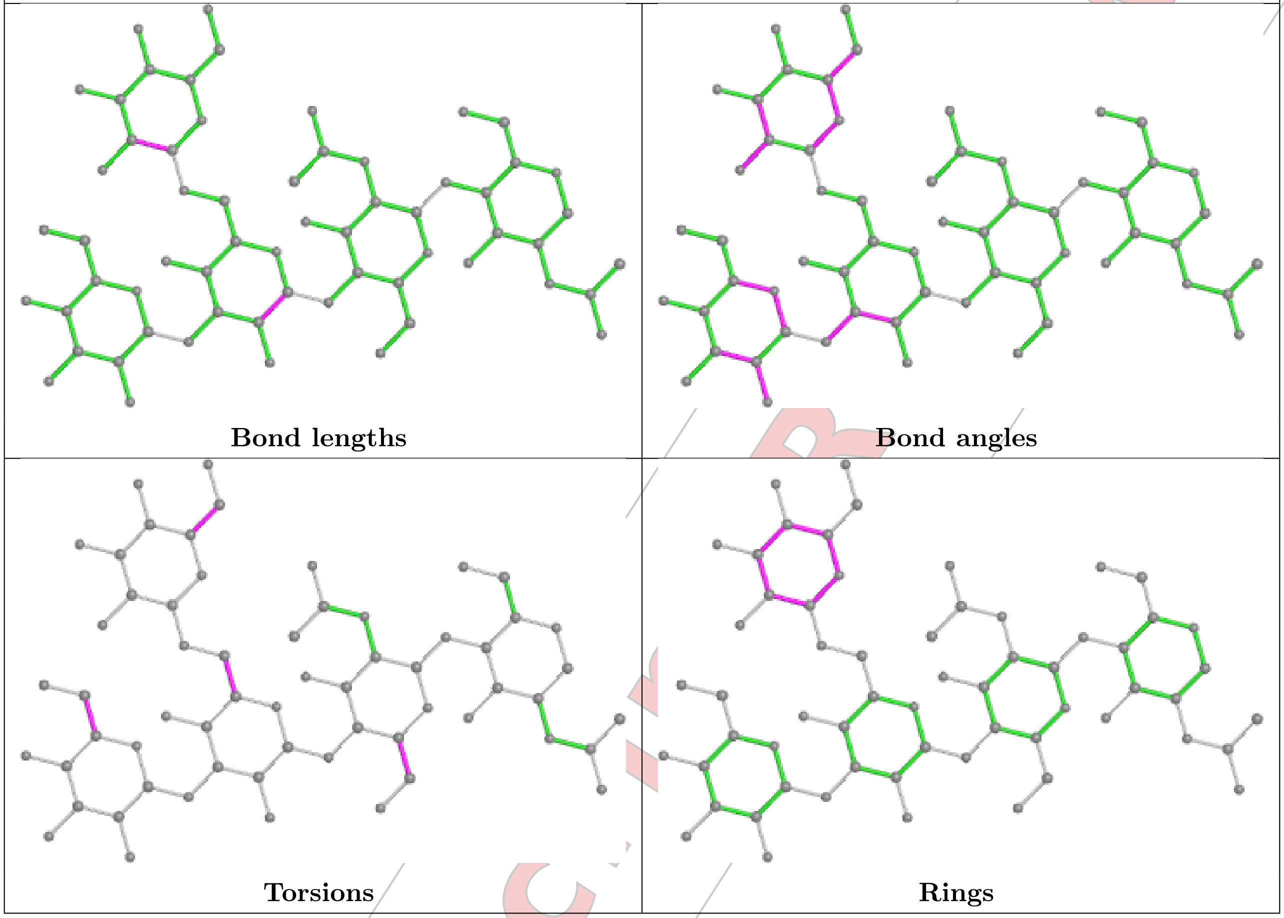

For Manuscript Review

Oligosaccharide Chain F

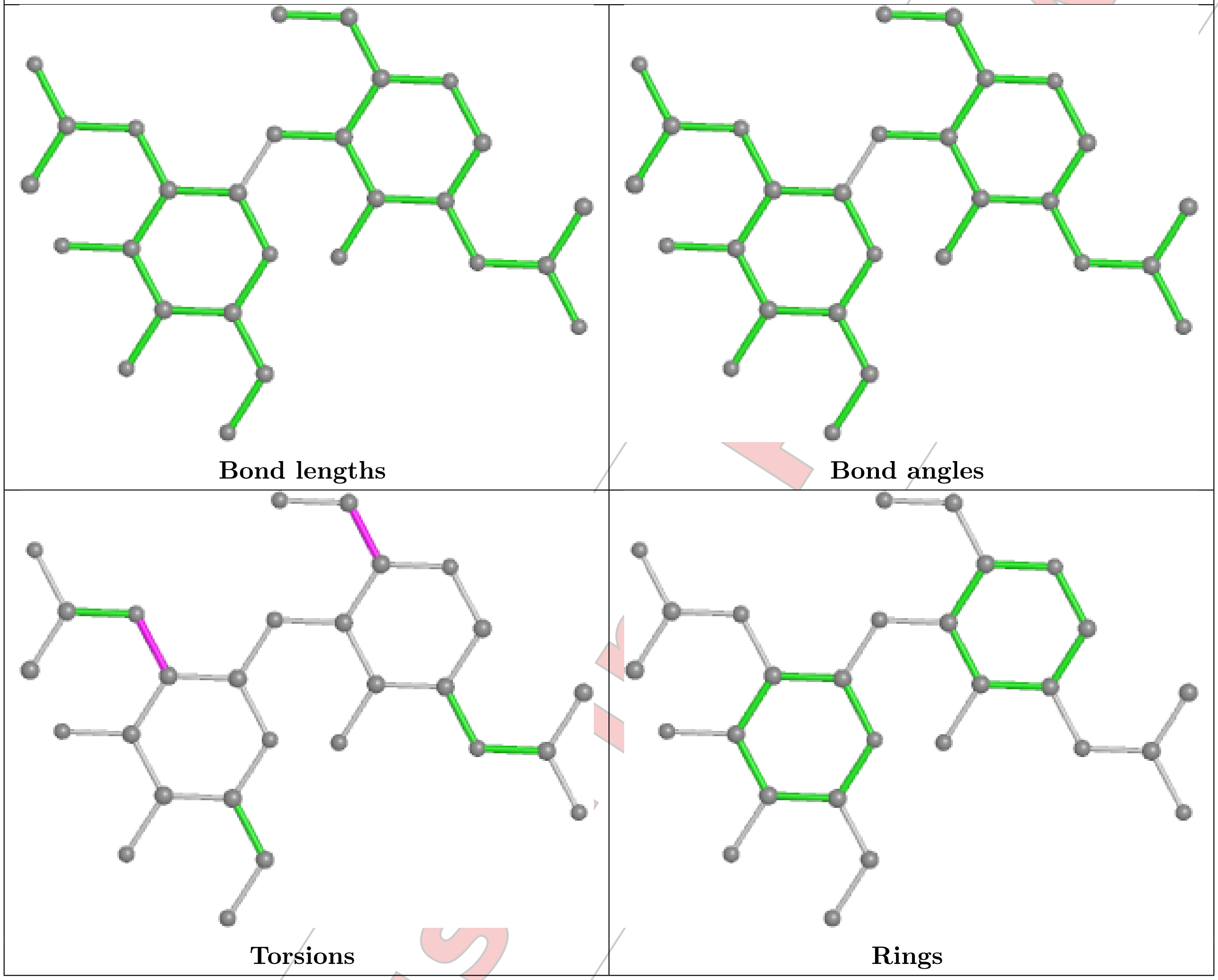

For Manuscript Review

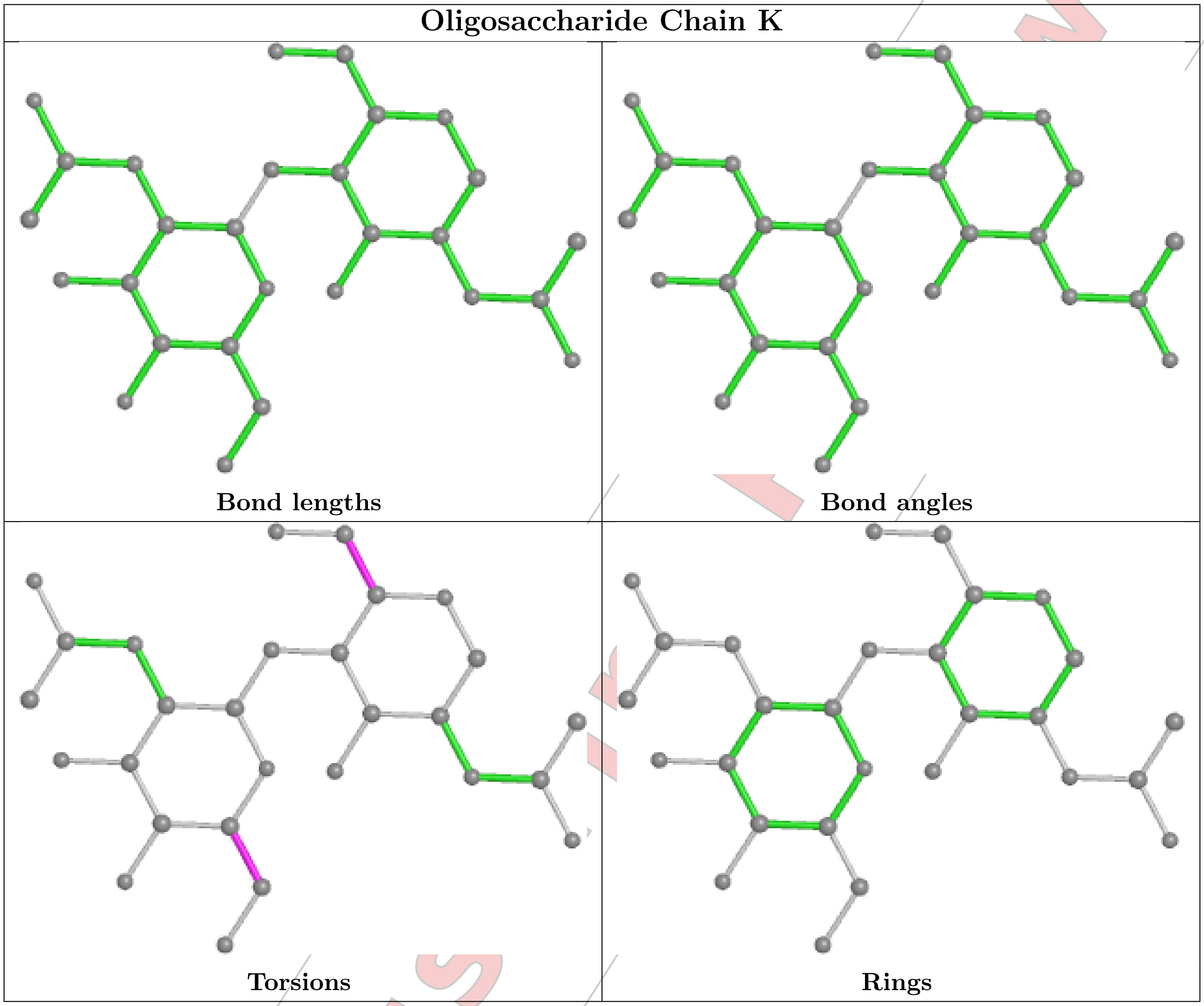

For Manuscript Review

Oligosaccharide Chain M

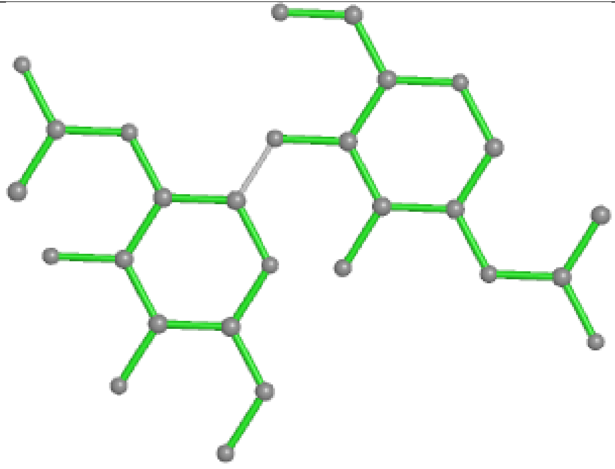

Bond lengths

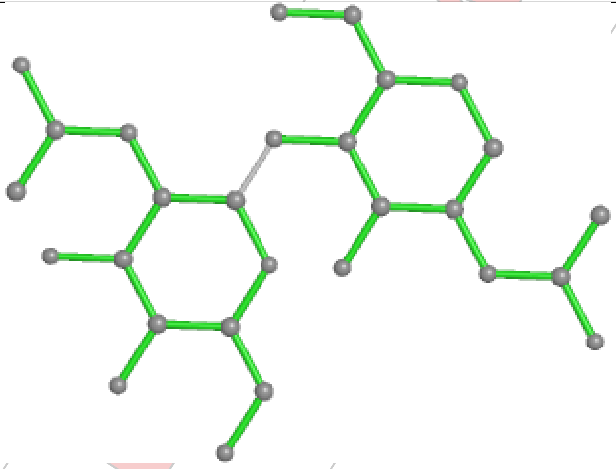

Bond angles

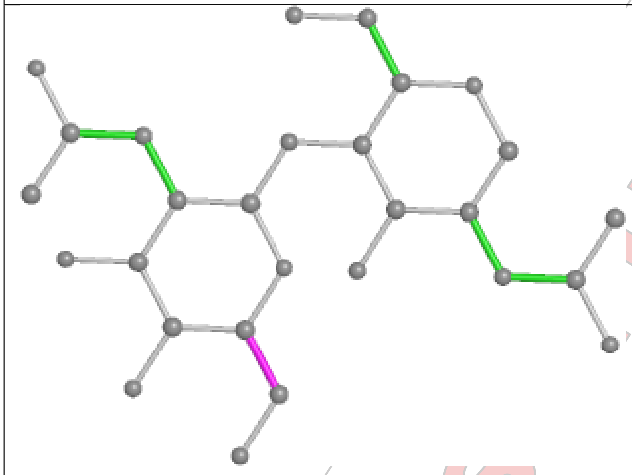

Torsions

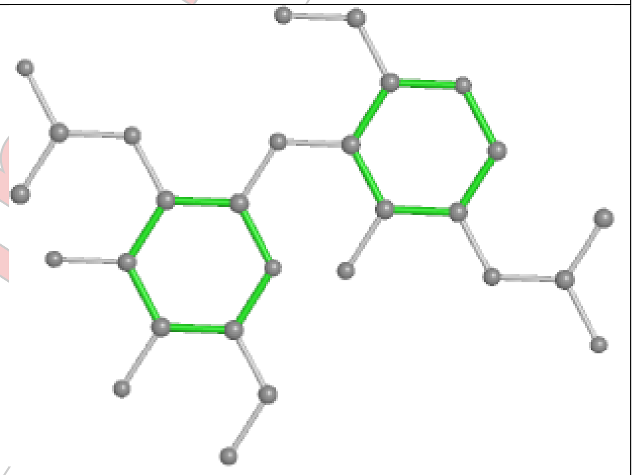

Rings

For Manuscript Review

Oligosaccharide Chain T

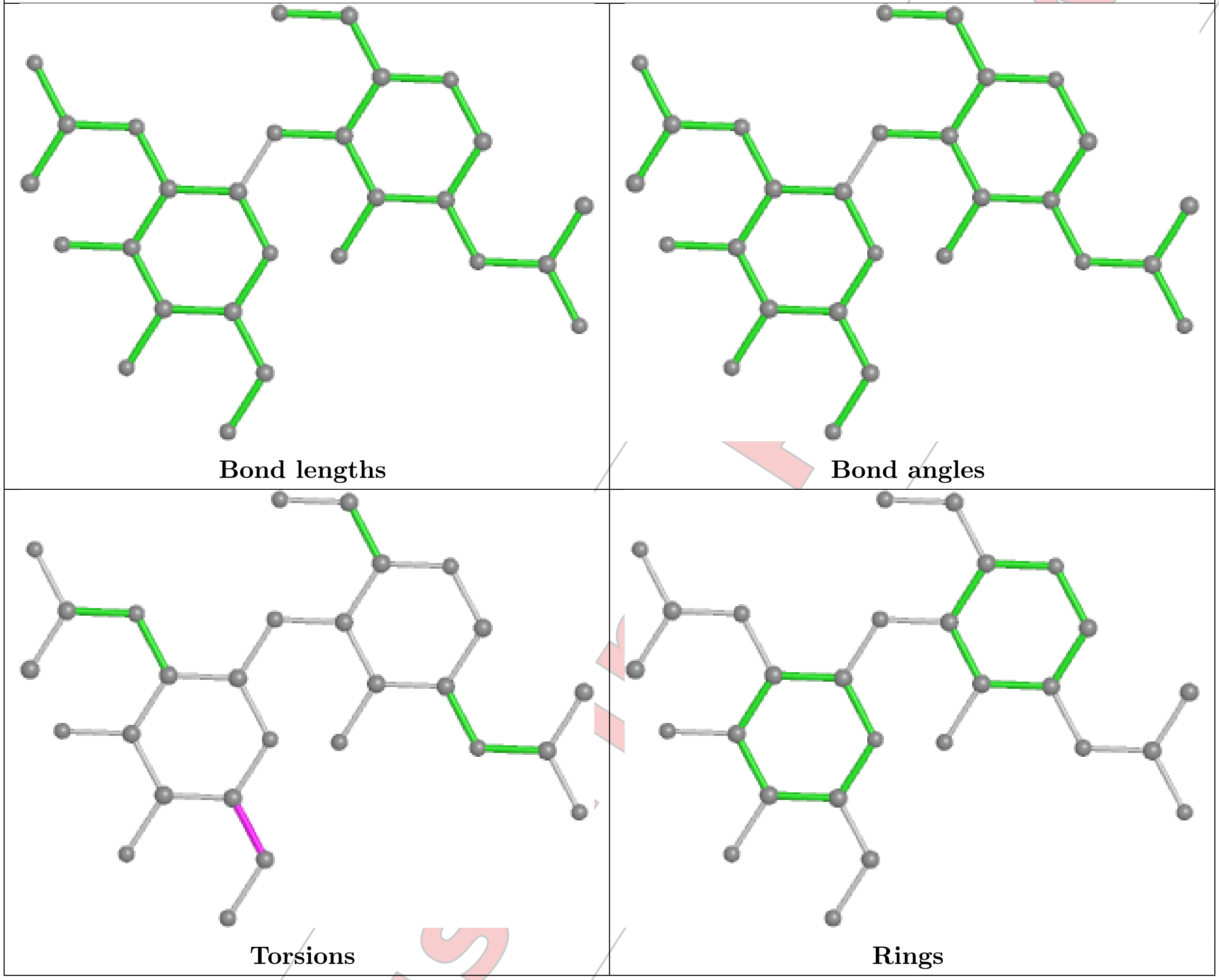

For Manuscript Review

Oligosaccharide Chain U	
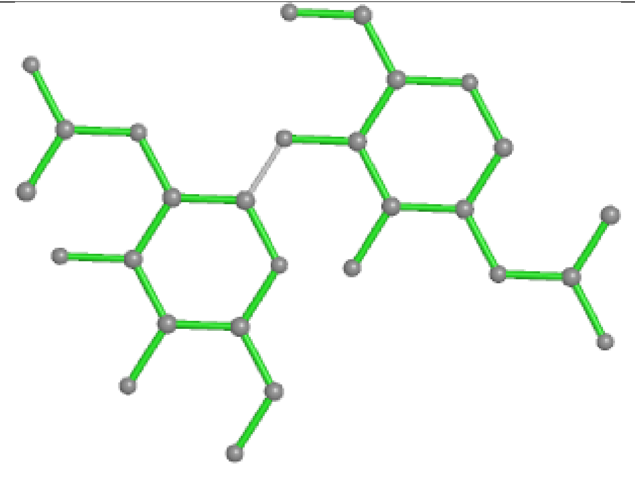 Bond lengths	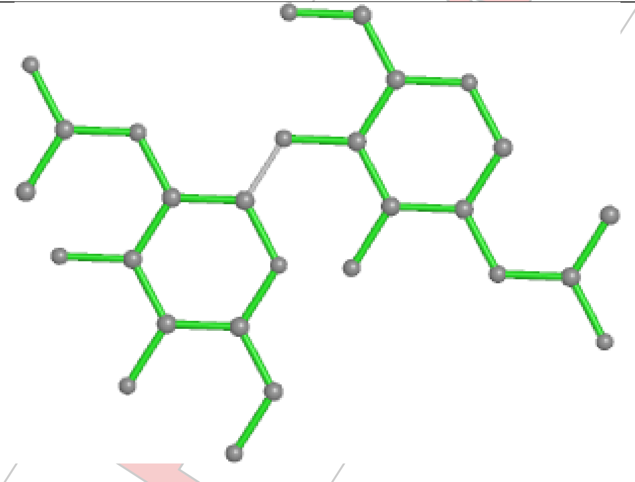 Bond angles
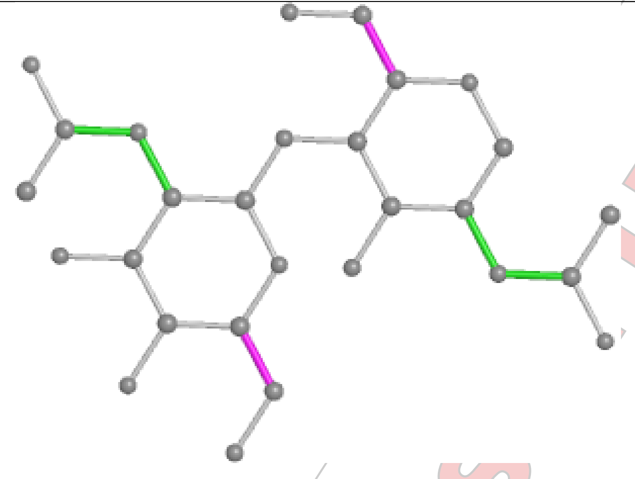 Torsions	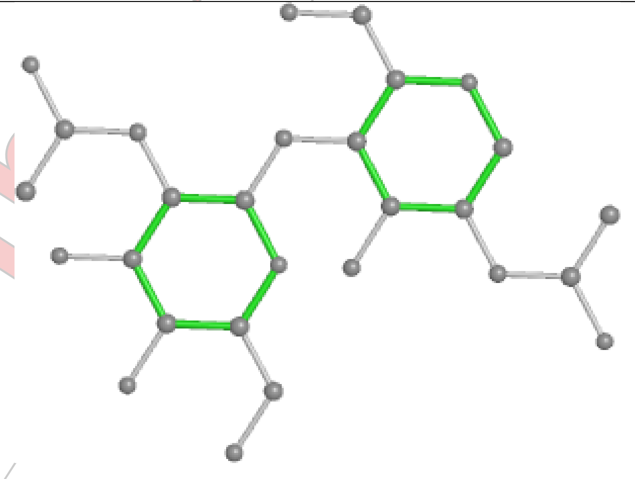 Rings

For Manuscript Review

Oligosaccharide Chain W

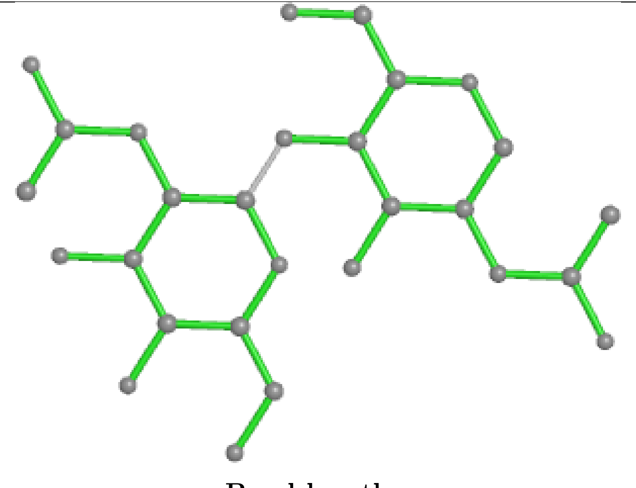

Bond lengths

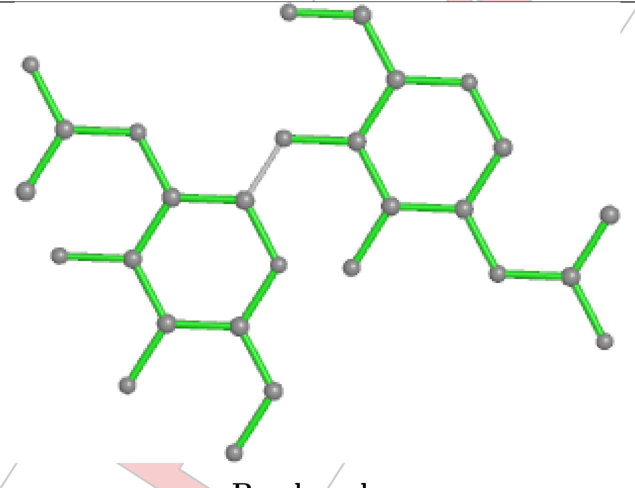

Bond angles

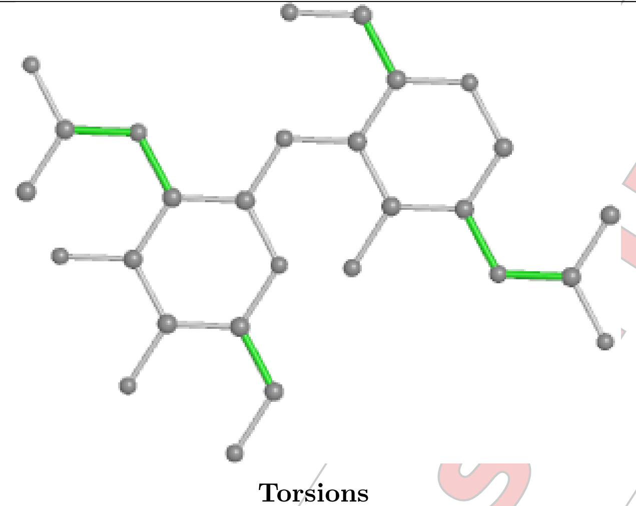

Torsions

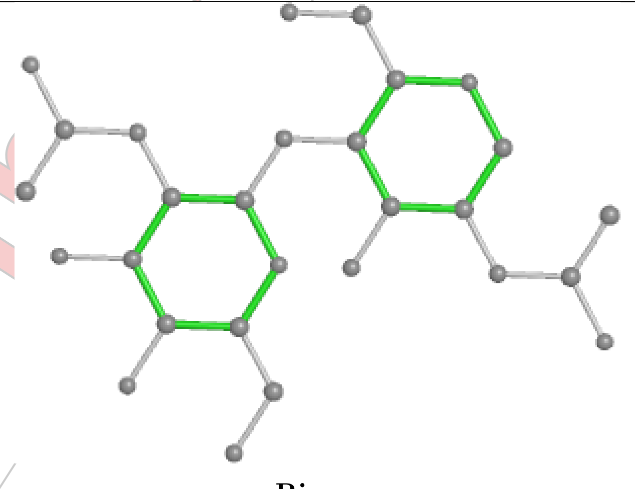

Rings

For Manuscript Review

Oligosaccharide Chain X

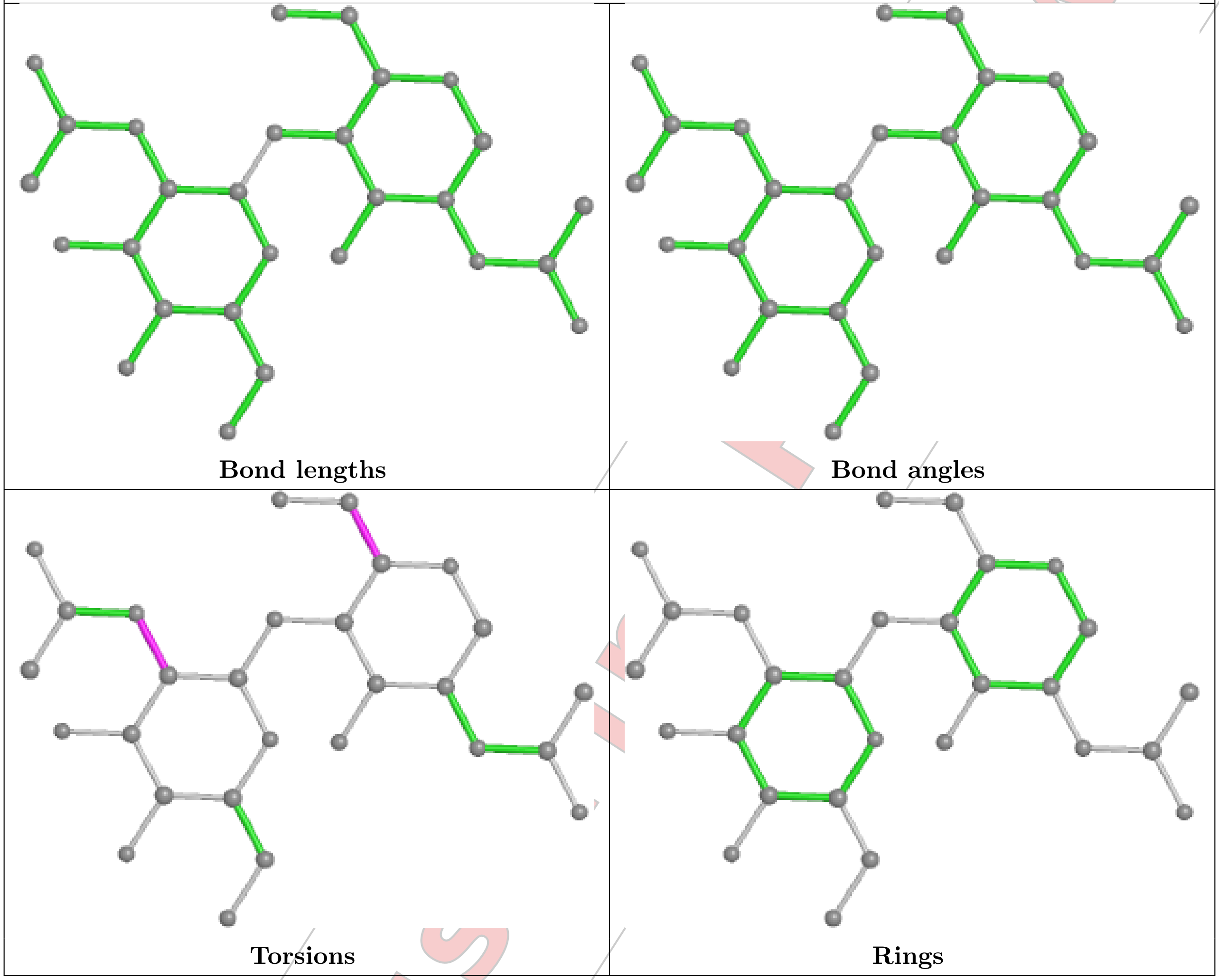

For Manuscript Review

Oligosaccharide Chain g

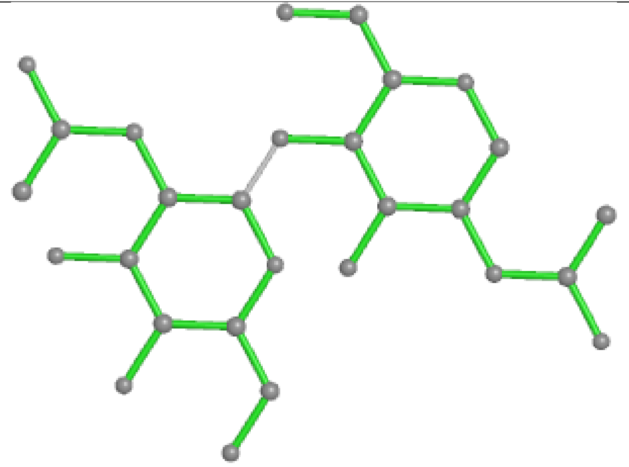

Bond lengths

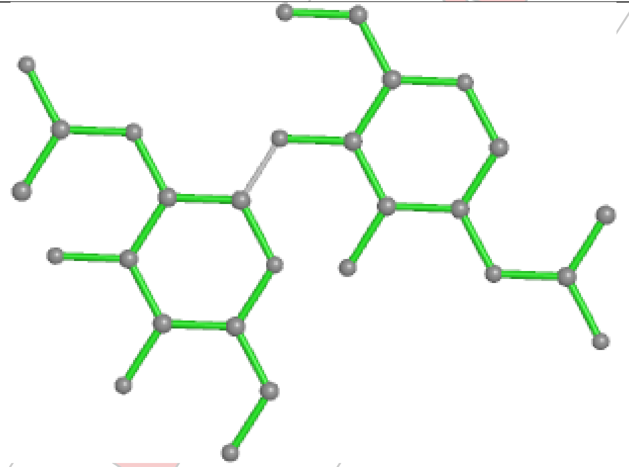

Bond angles

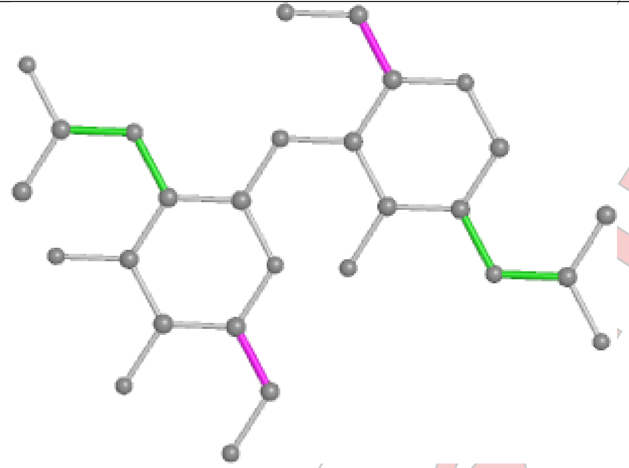

Torsions

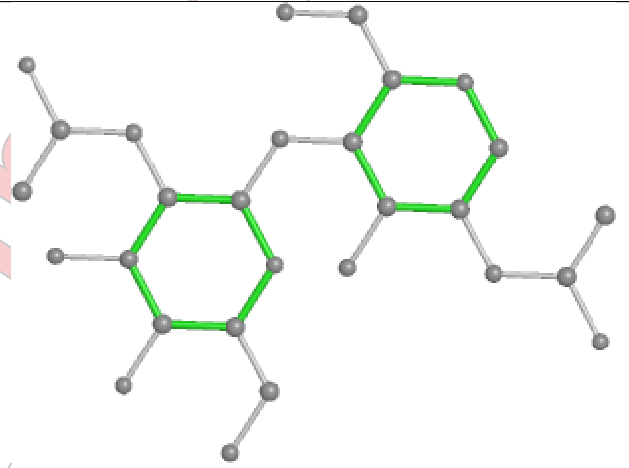

Rings

Oligosaccharide Chain h	
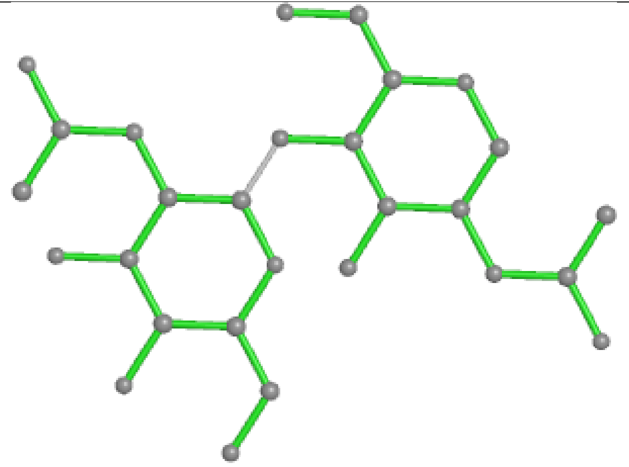 Bond lengths	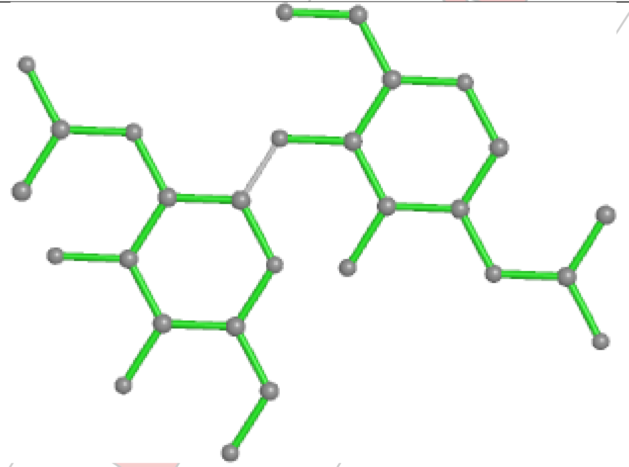 Bond angles
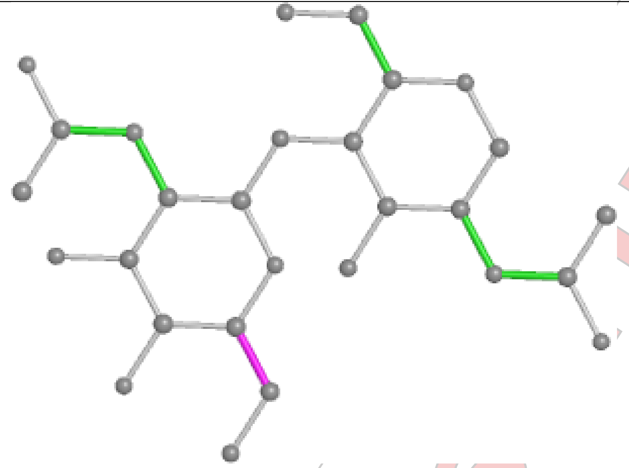 Torsions	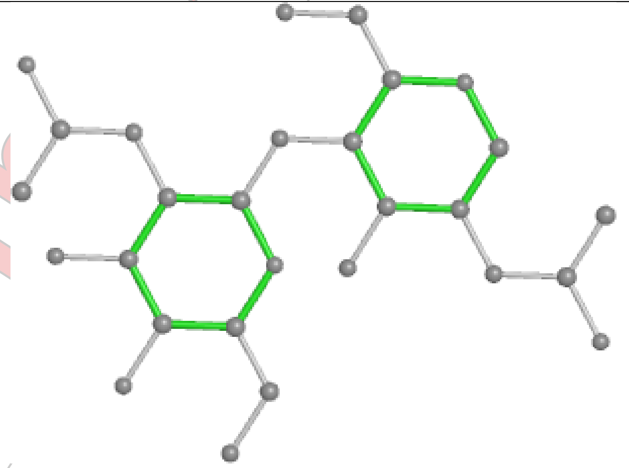 Rings

For Manuscript Review

Oligosaccharide Chain j

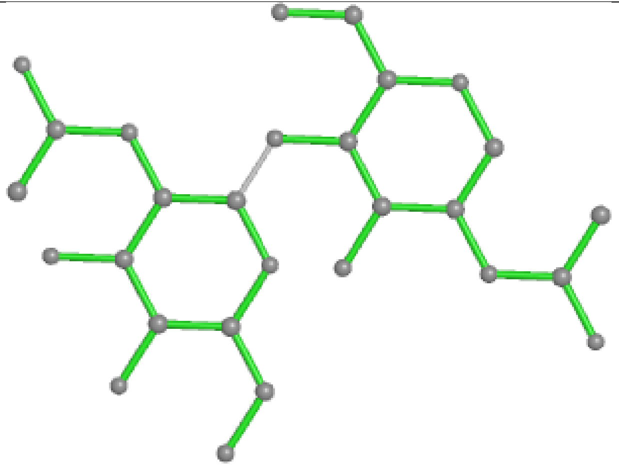 Bond lengths	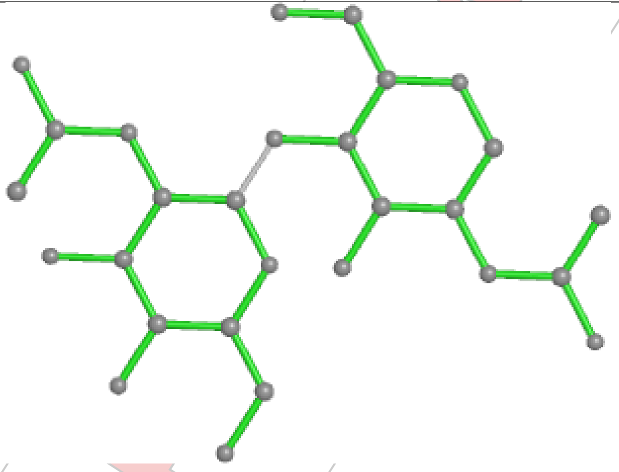 Bond angles
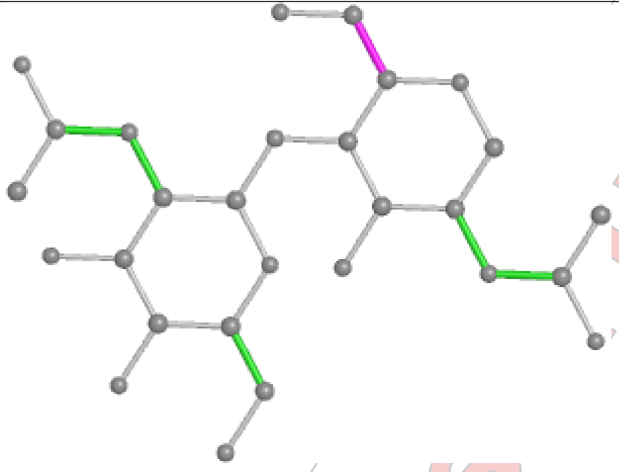 Torsions	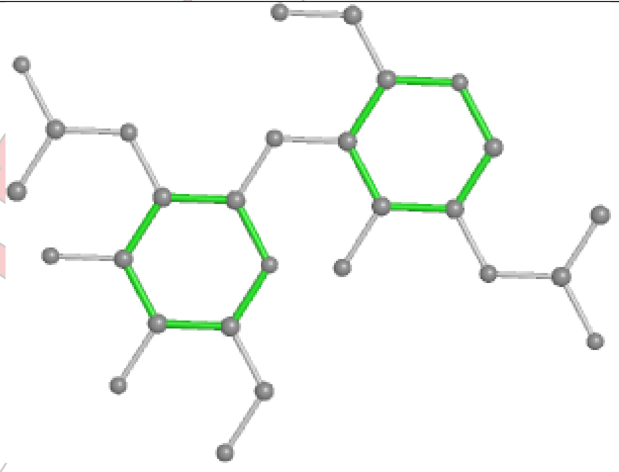 Rings

For Manuscript Review

Oligosaccharide Chain l

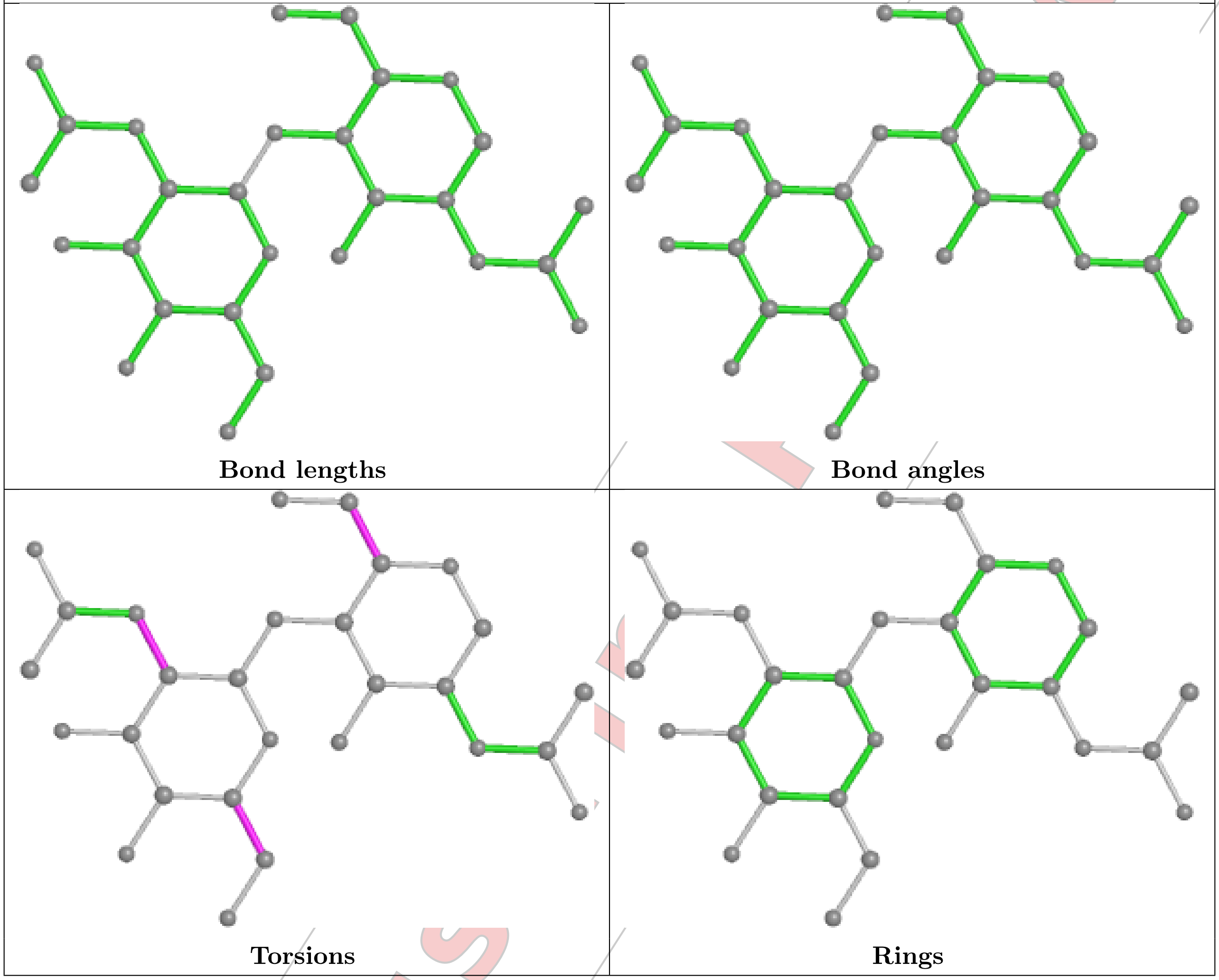

For Manuscript Review

Oligosaccharide Chain o

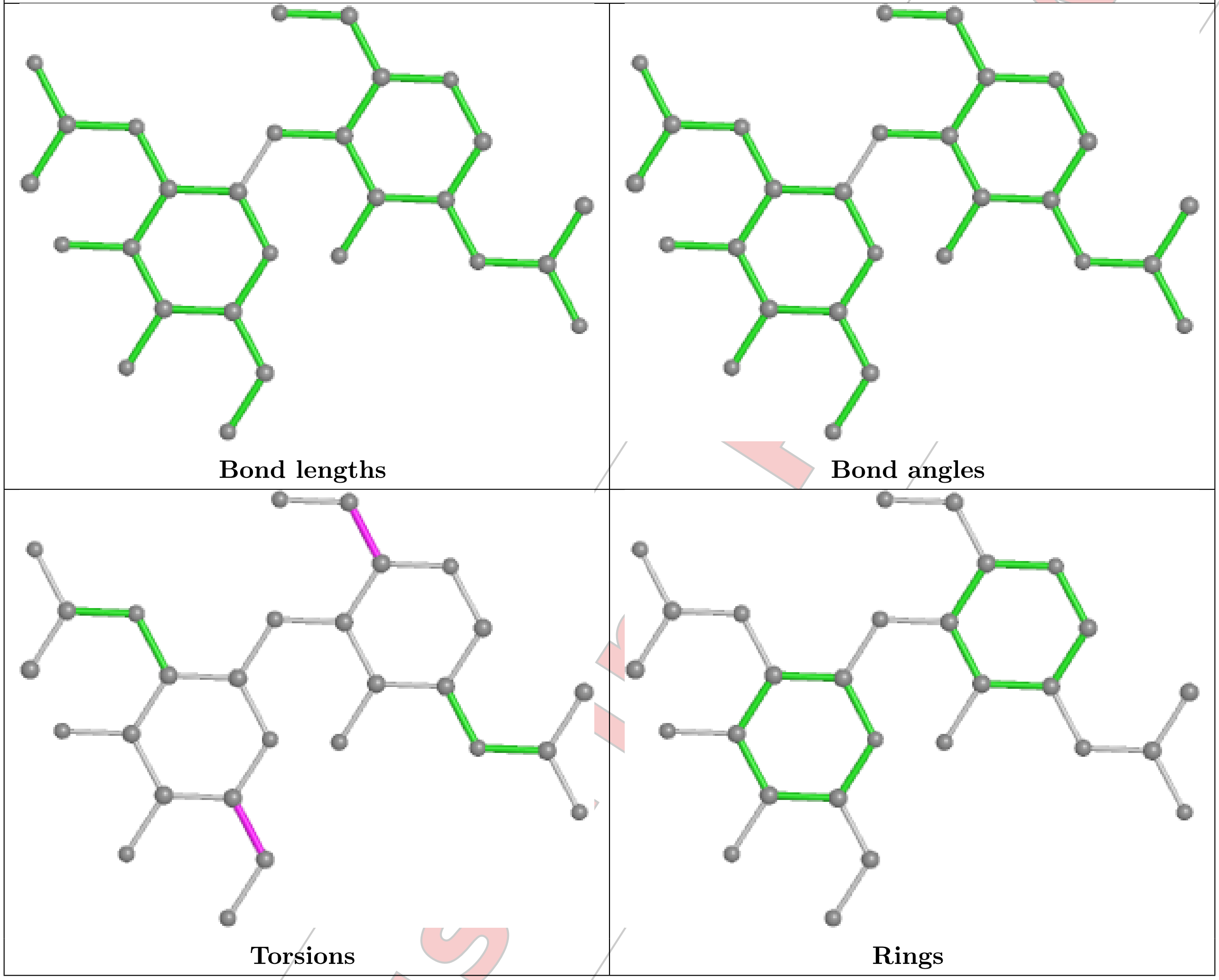

For Manuscript Review

Oligosaccharide Chain p

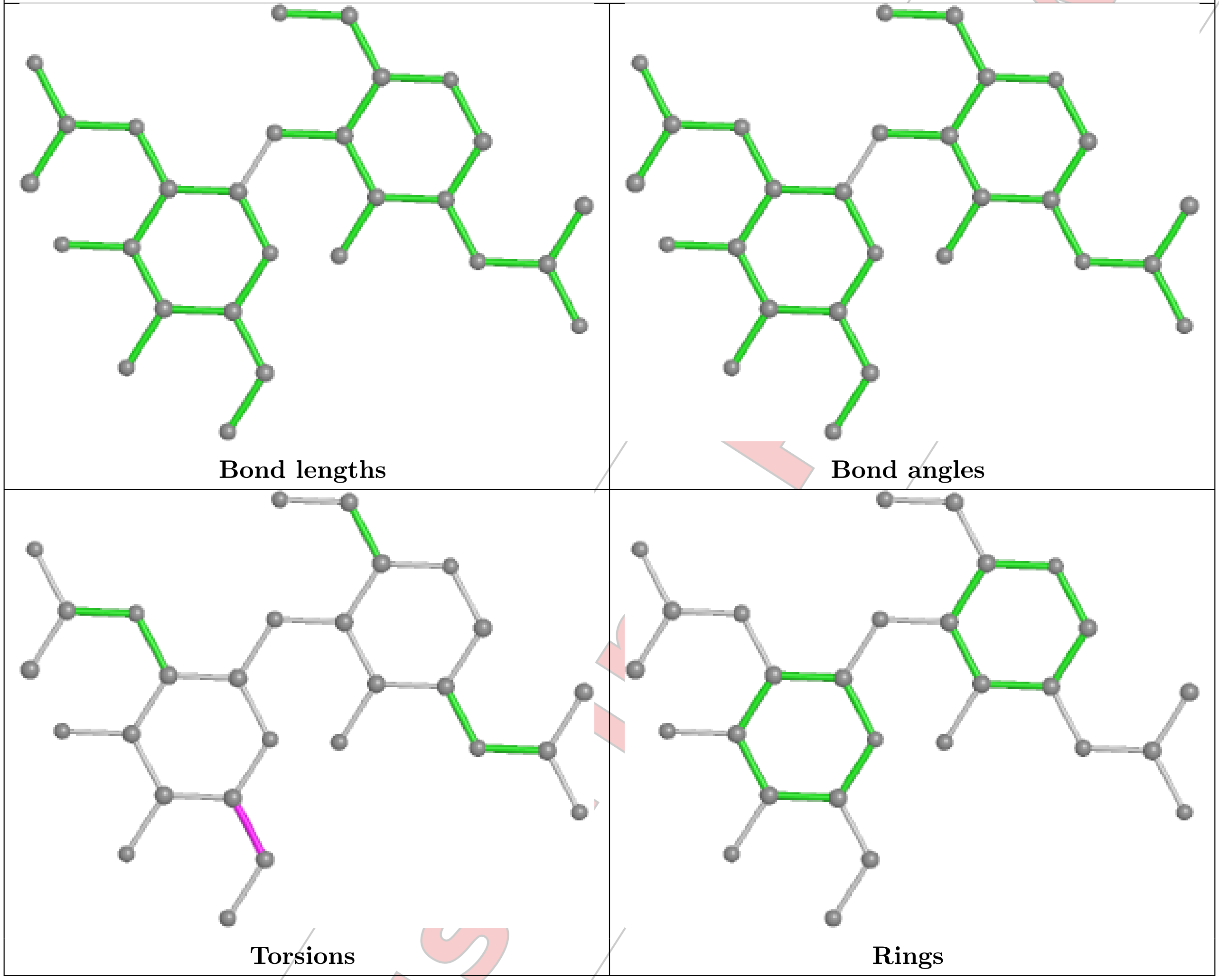

For Manuscript Review

Oligosaccharide Chain r

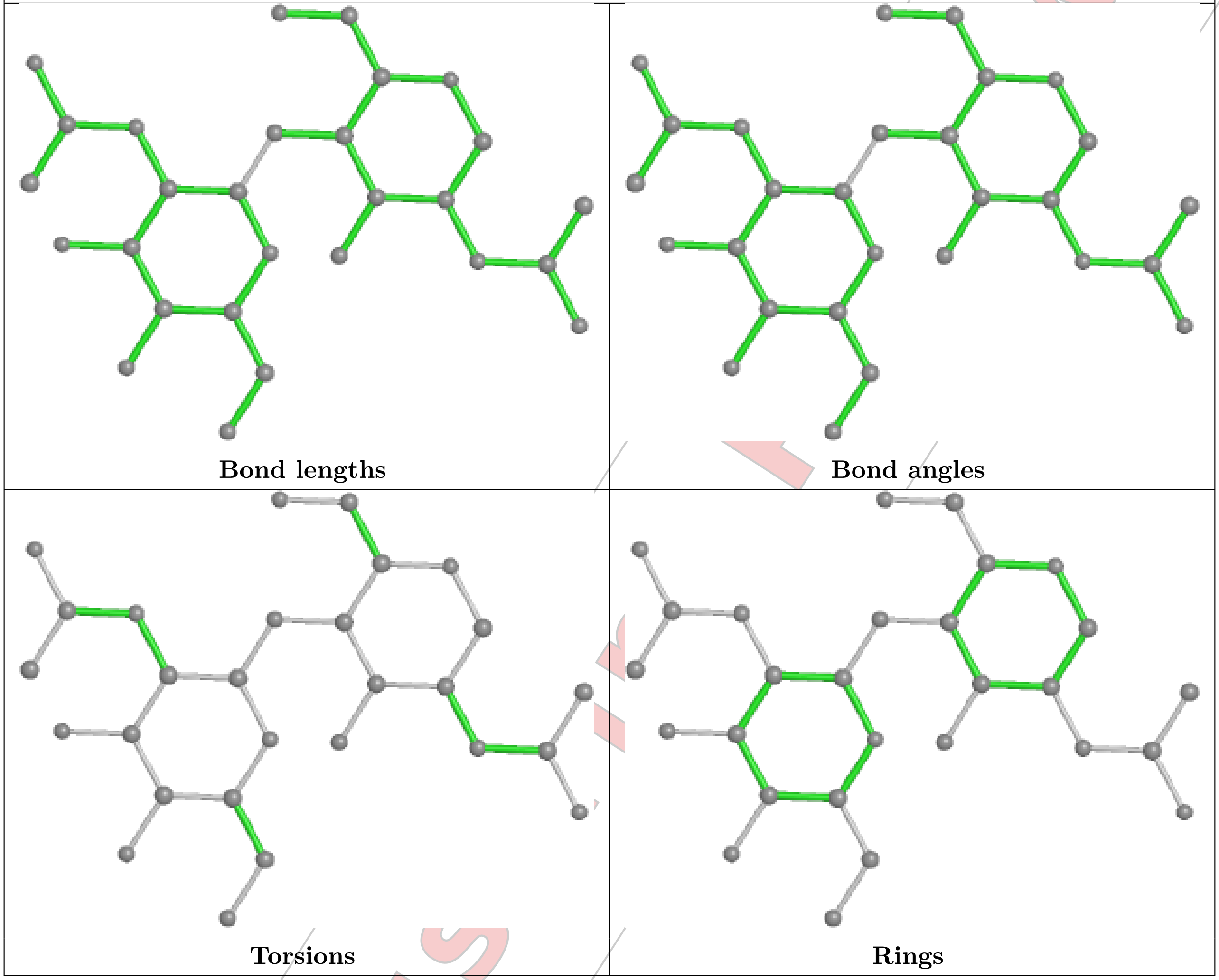

For Manuscript Review

Oligosaccharide Chain I

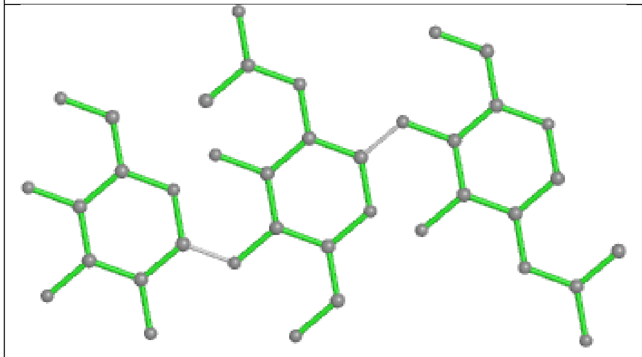

Bond lengths

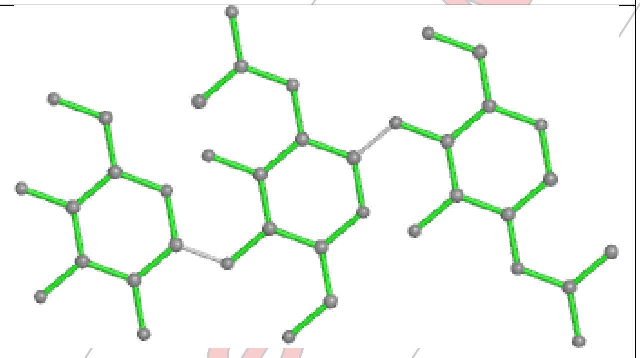

Bond angles

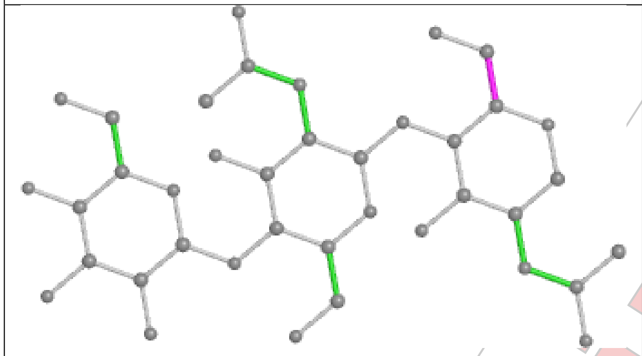

Torsions

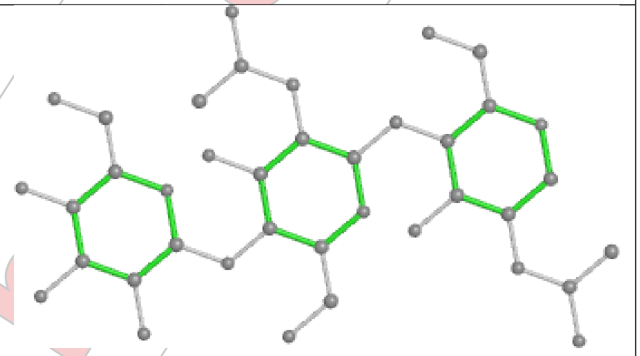

Rings

Oligosaccharide Chain J

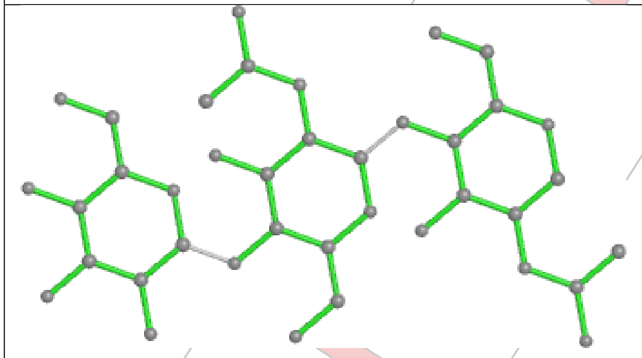

Bond lengths

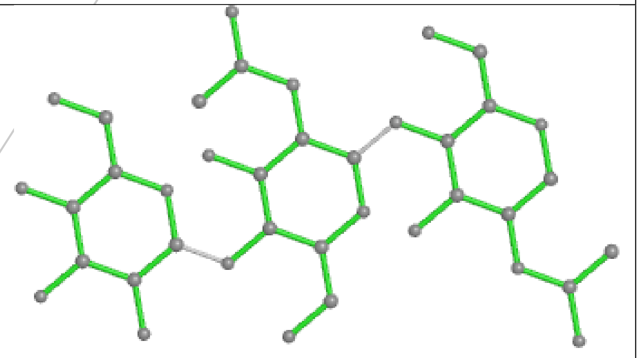

Bond angles

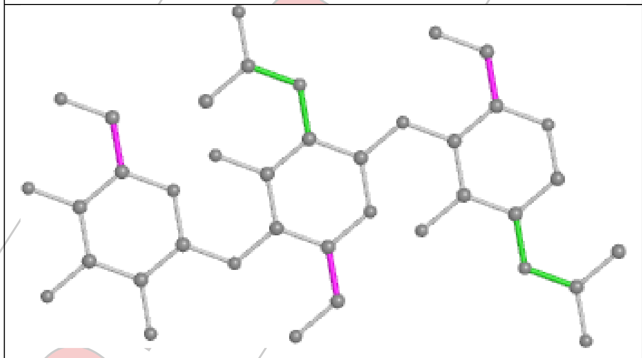

Torsions

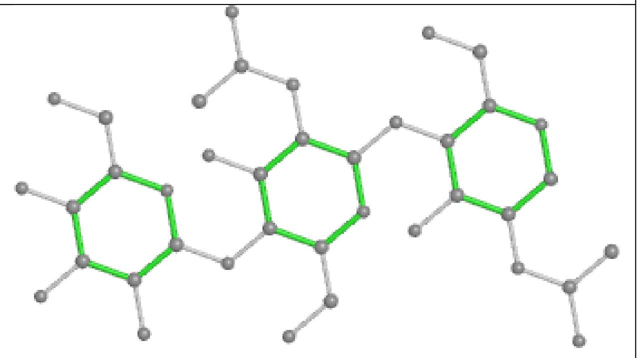

Rings

Oligosaccharide Chain Y

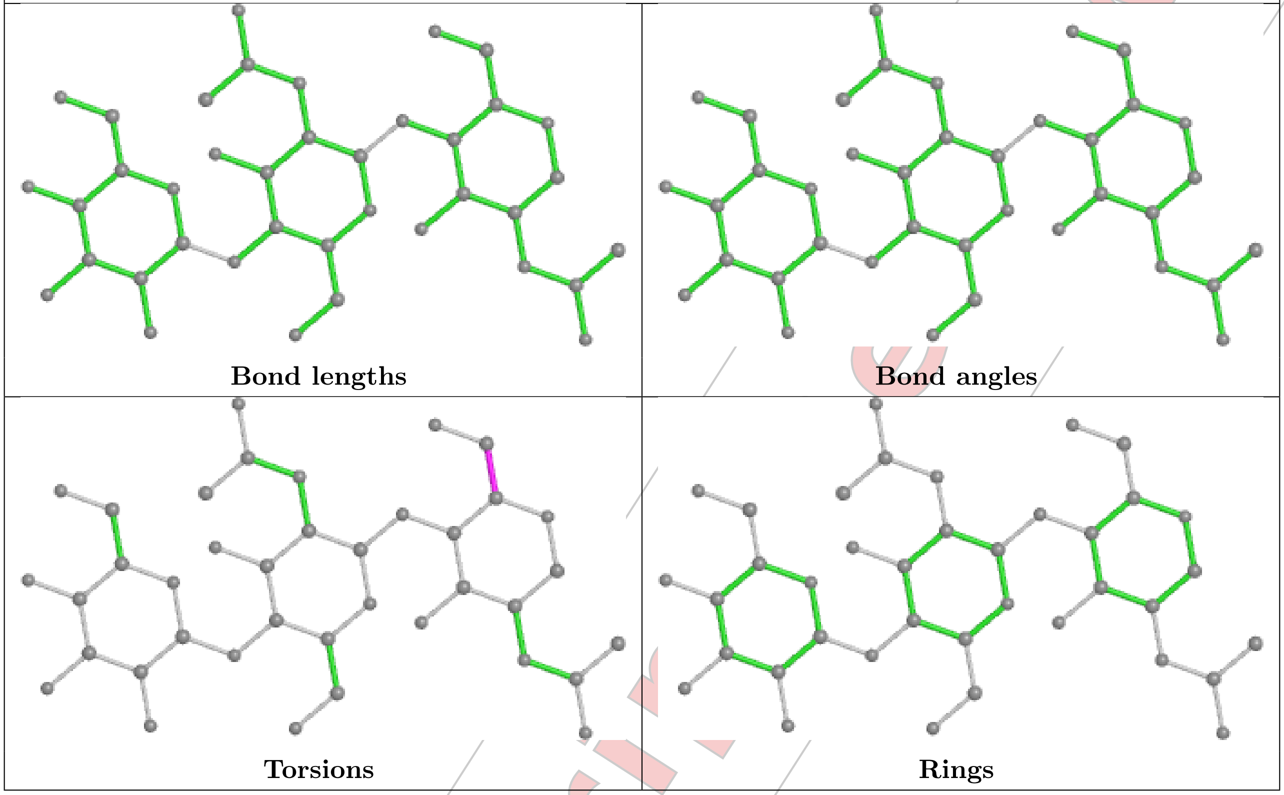

Oligosaccharide Chain Z

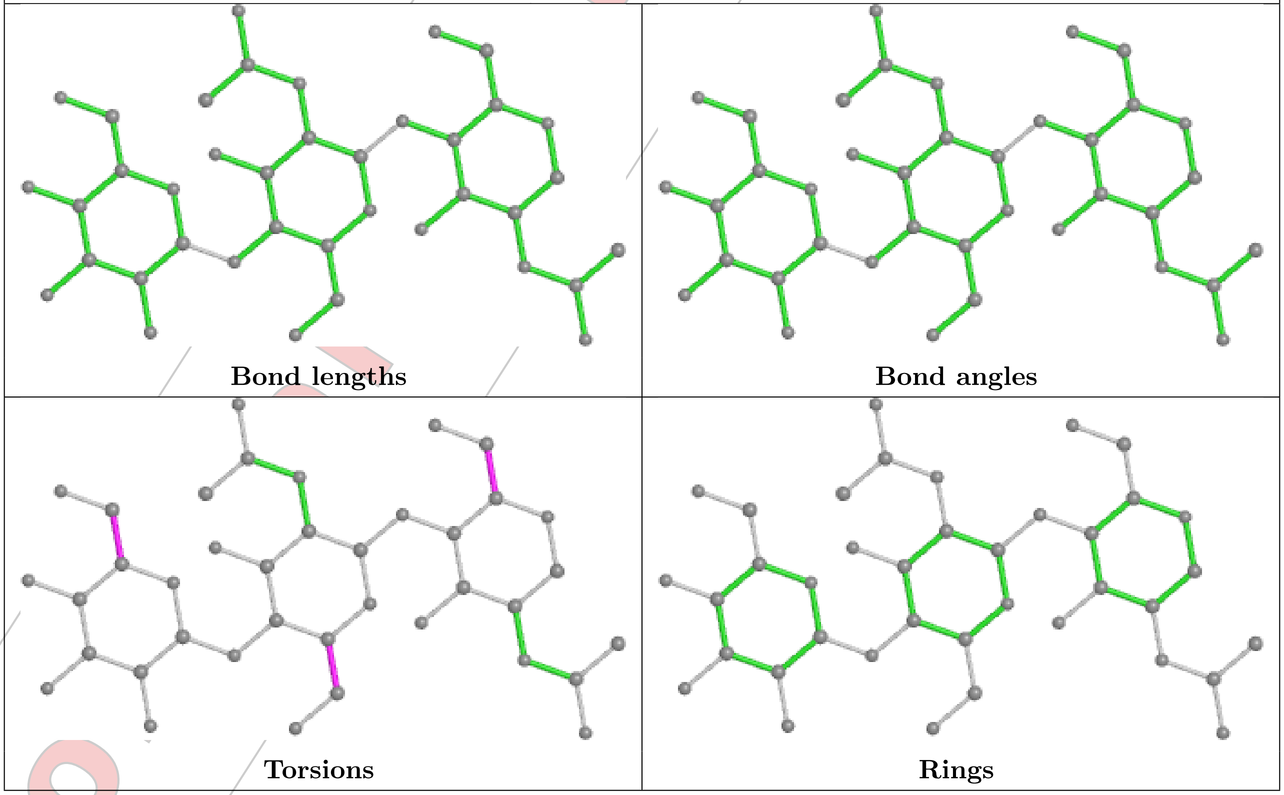

Oligosaccharide Chain m

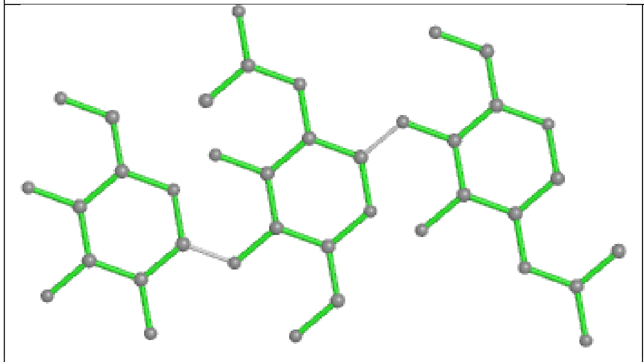
Bond lengths

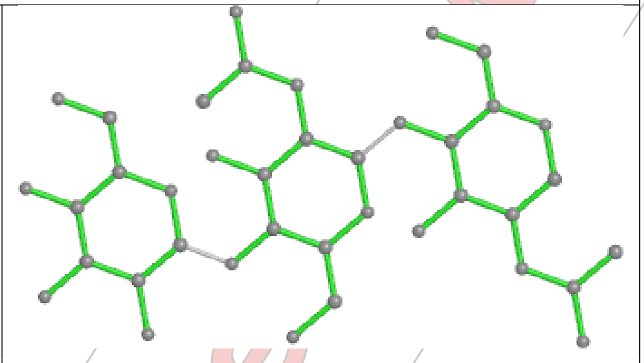
Bond angles

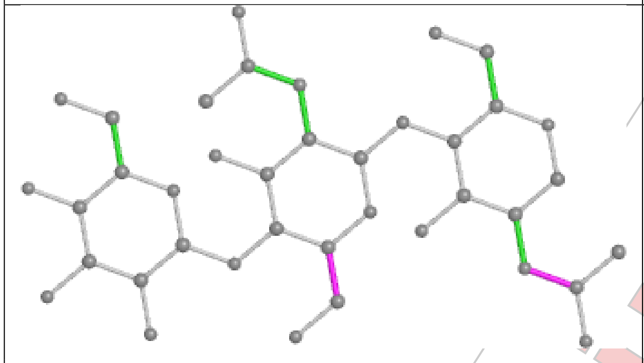
Torsions

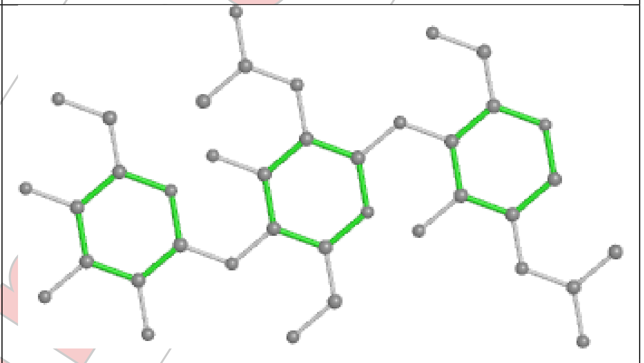
Rings

Oligosaccharide Chain n

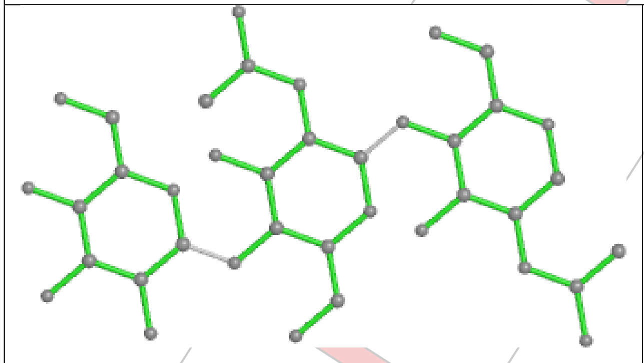
Bond lengths

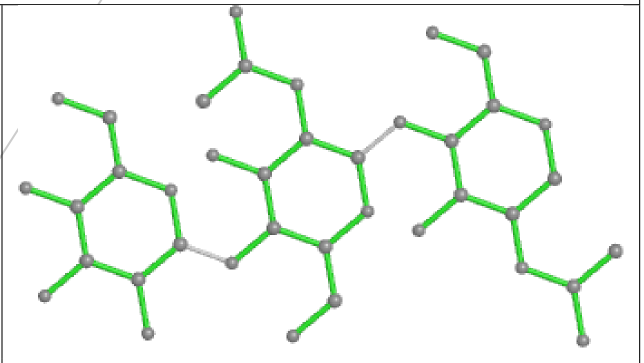
Bond angles

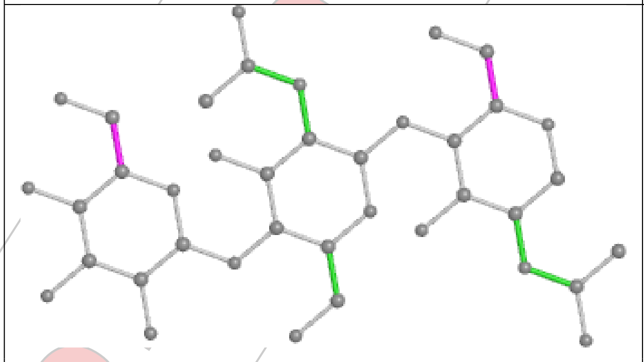
Torsions

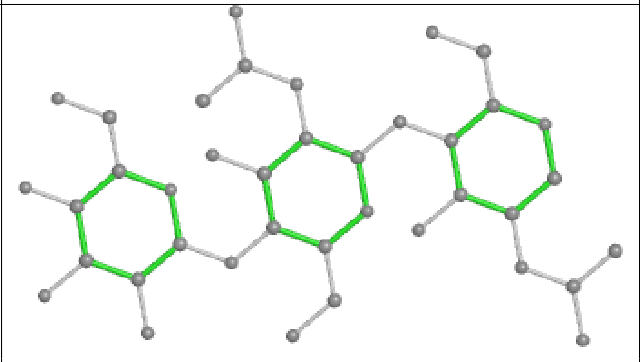
Rings

Oligosaccharide Chain N

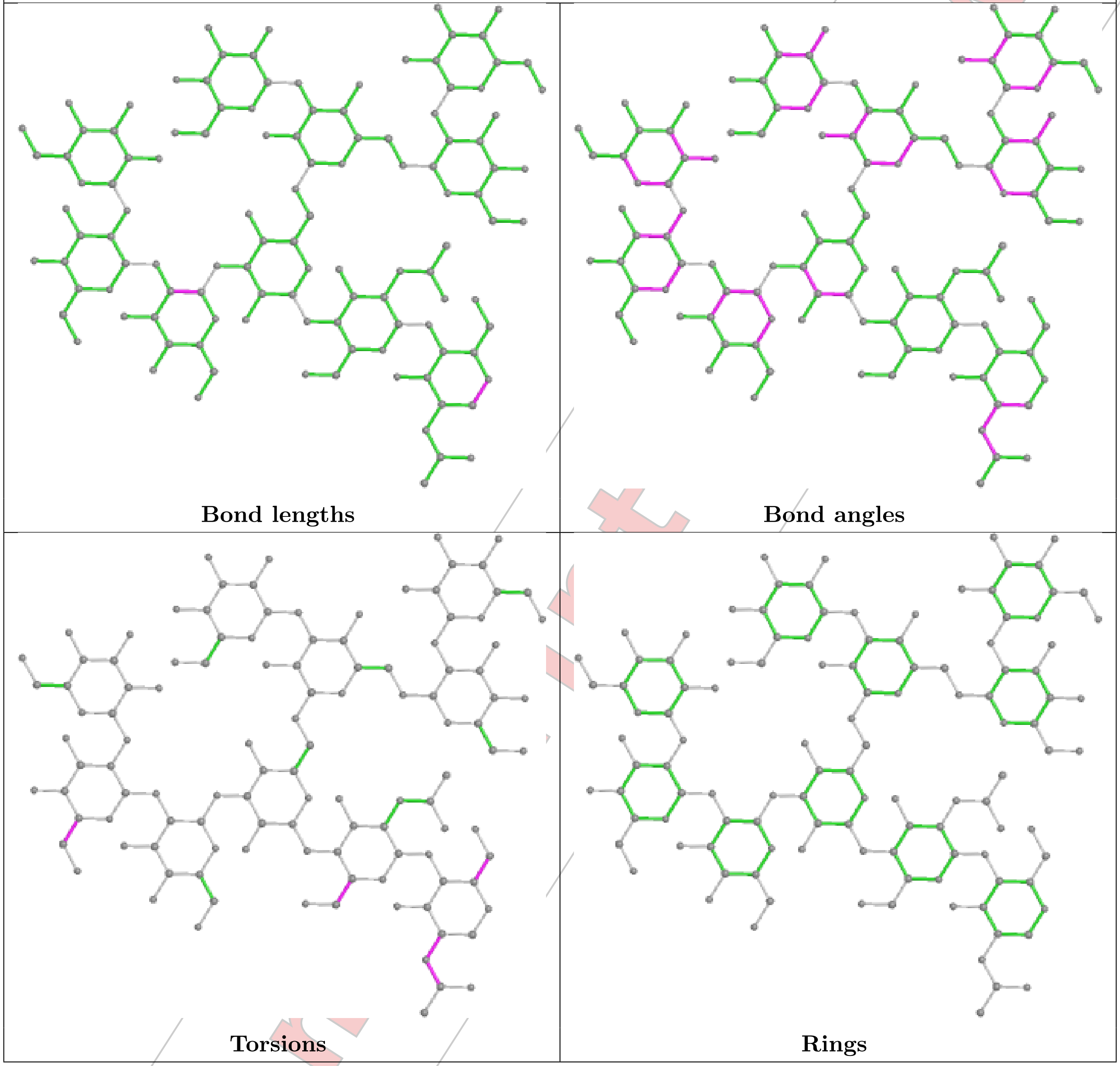

For Manuscript Review

Oligosaccharide Chain i

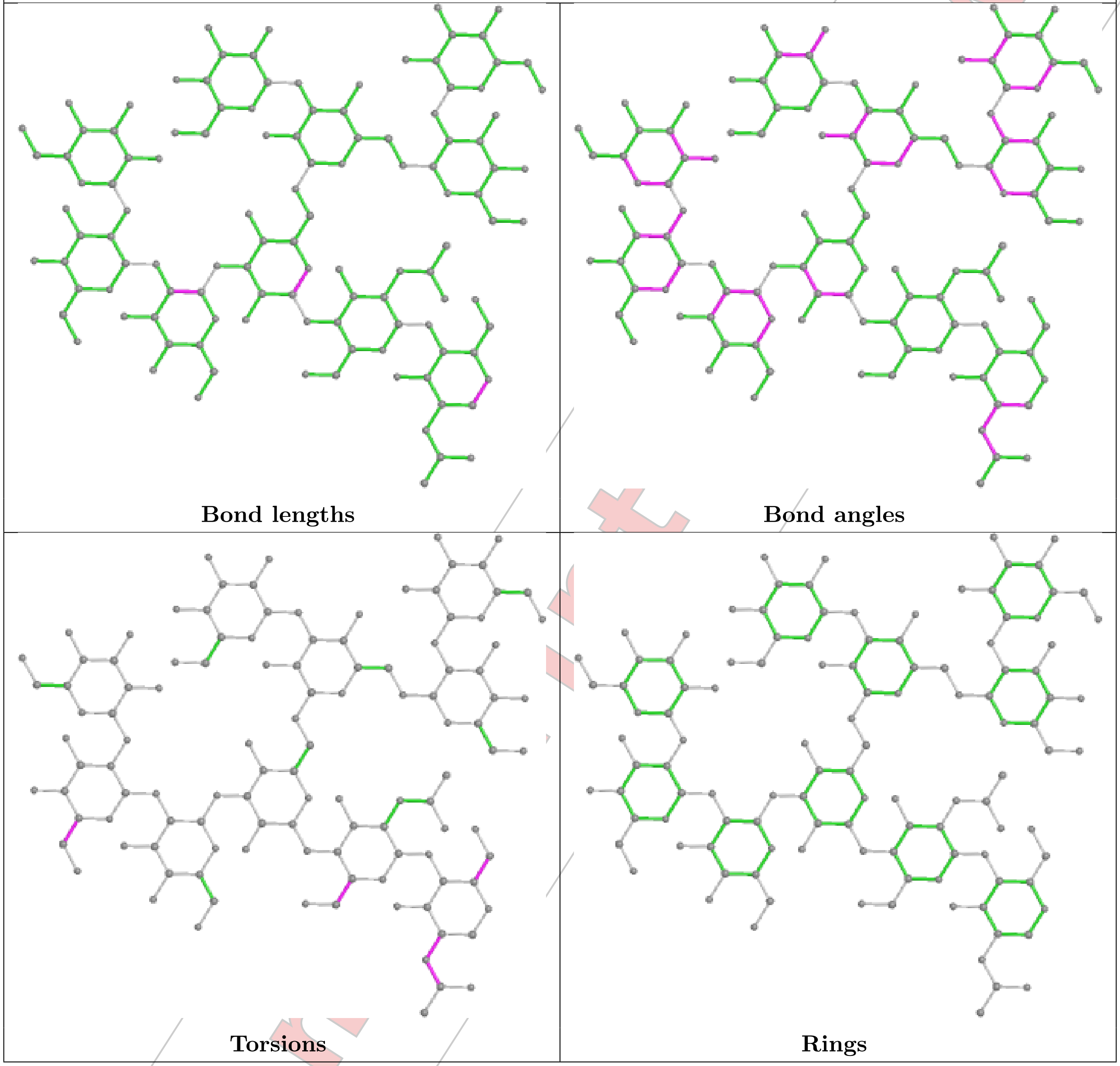

For Manuscript Review

Oligosaccharide Chain q

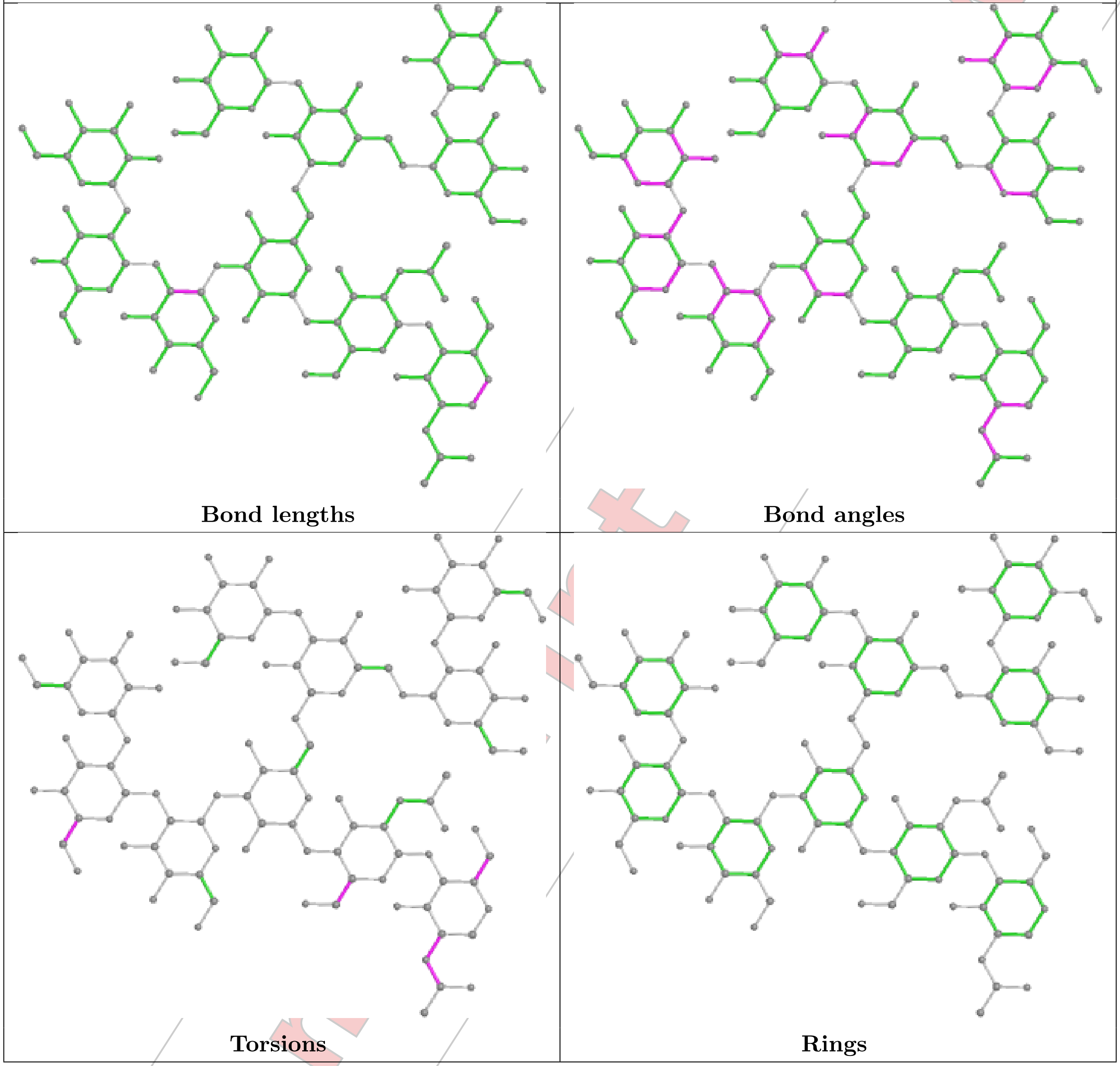

For Manuscript Review

Oligosaccharide Chain V

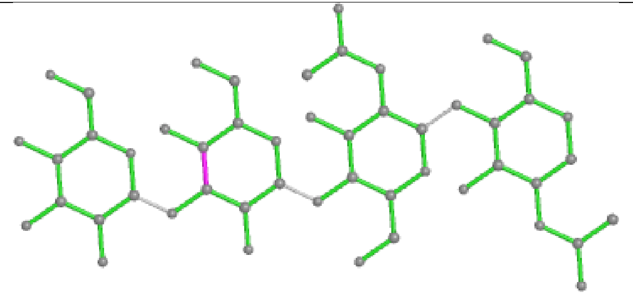

Bond lengths

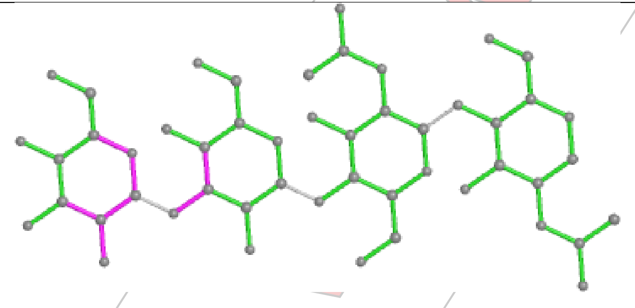

Bond angles

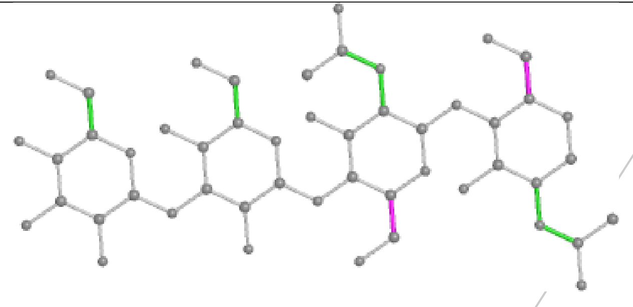

Torsions

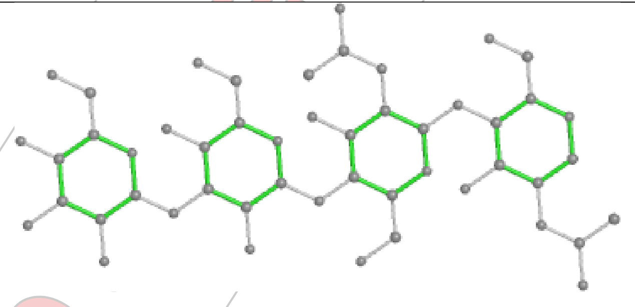

Rings

Oligosaccharide Chain k

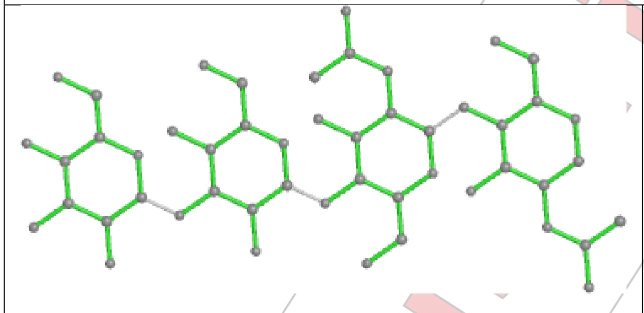

Bond lengths

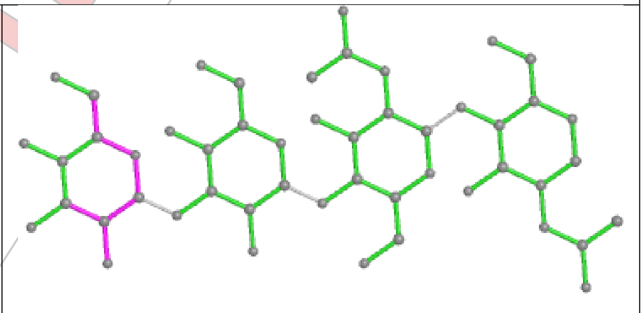

Bond angles

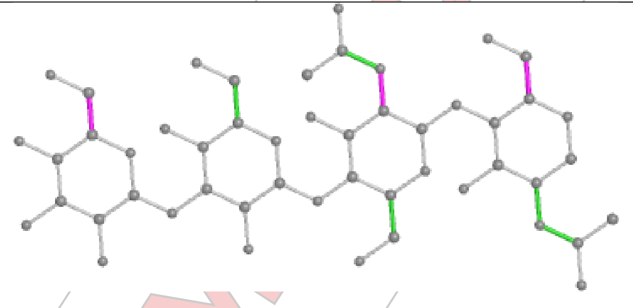

Torsions

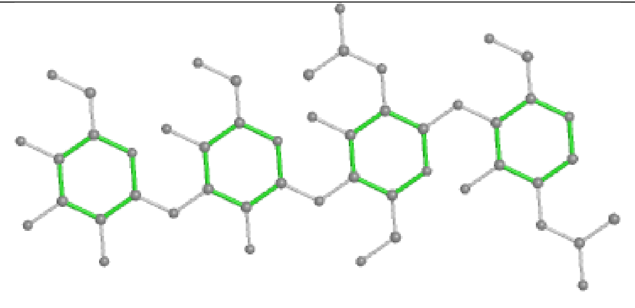

Rings

5.6 Ligand geometry (i)

31 ligands are modelled in this entry.

In the following table, the Counts columns list the number of bonds (or angles) for which Mogul statistics could be retrieved, the number of bonds (or angles) that are observed in the model and the number of bonds (or angles) that are defined in the Chemical Component Dictionary. The

For Manuscript Review

Link column lists molecule types, if any, to which the group is linked. The Z score for a bond length (or angle) is the number of standard deviations the observed value is removed from the expected value. A bond length (or angle) with $|Z| > 2$ is considered an outlier worth inspection. RMSZ is the root-mean-square of all Z scores of the bond lengths (or angles).

Mol	Type	Chain	Res	Link	Bond lengths			Bond angles		
					Counts	RMSZ	$\#\|Z\| > 2$	Counts	RMSZ	$\#\|Z\| > 2$
12	NAG	G	602	5	14,14,15	0.30	0	17,19,21	0.47	0
12	NAG	a	701	1	14,14,15	0.27	0	17,19,21	0.47	0
12	NAG	e	608	5	14,14,15	0.31	0	17,19,21	0.49	0
12	NAG	a	702	1	14,14,15	0.26	0	17,19,21	0.40	0
12	NAG	G	606	5	14,14,15	0.30	0	17,19,21	0.48	0
12	NAG	G	608	5	14,14,15	0.43	0	17,19,21	0.56	0
12	NAG	e	605	5	14,14,15	0.25	0	17,19,21	0.47	0
12	NAG	R	602	5	14,14,15	0.26	0	17,19,21	0.41	0
12	NAG	e	607	5	14,14,15	0.19	0	17,19,21	0.43	0
12	NAG	G	604	5	14,14,15	0.24	0	17,19,21	0.47	0
12	NAG	G	607	5	14,14,15	0.21	0	17,19,21	0.44	0
12	NAG	R	603	5	14,14,15	0.23	0	17,19,21	0.50	0
12	NAG	G	601	5	14,14,15	0.40	0	17,19,21	0.48	0
12	NAG	H	701	1	14,14,15	0.26	0	17,19,21	0.47	0
12	NAG	R	604	5	14,14,15	0.28	0	17,19,21	0.44	0
12	NAG	R	605	5	14,14,15	0.28	0	17,19,21	0.49	0
12	NAG	G	603	5	14,14,15	0.28	0	17,19,21	0.41	0
12	NAG	G	605	5	14,14,15	0.27	0	17,19,21	0.44	0
12	NAG	H	703	1	14,14,15	0.18	0	17,19,21	0.42	0
12	NAG	a	703	1	14,14,15	0.22	0	17,19,21	0.42	0
12	NAG	B	702	1	14,14,15	0.20	0	17,19,21	0.53	0
12	NAG	e	606	5	14,14,15	0.31	0	17,19,21	0.49	0
12	NAG	H	702	1	14,14,15	0.28	0	17,19,21	1.51	2 (11%)
12	NAG	R	606	5	14,14,15	0.20	0	17,19,21	0.43	0
12	NAG	e	602	5	14,14,15	0.27	0	17,19,21	0.47	0
12	NAG	e	604	5	14,14,15	0.21	0	17,19,21	0.49	0
12	NAG	e	603	5	14,14,15	0.27	0	17,19,21	0.41	0
12	NAG	e	601	5	14,14,15	0.39	0	17,19,21	0.45	0
12	NAG	B	701	1	14,14,15	0.27	0	17,19,21	0.46	0
12	NAG	R	601	5	14,14,15	0.39	0	17,19,21	0.49	0
12	NAG	B	703	1	14,14,15	0.20	0	17,19,21	0.43	0

In the following table, the Chirals column lists the number of chiral outliers, the number of chiral centers analysed, the number of these observed in the model and the number defined in the Chemical Component Dictionary. Similar counts are reported in the Torsion and Rings columns. '-' means no outliers of that kind were identified.

For Manuscript Review

Mol	Type	Chain	Res	Link	Chirals	Torsions	Rings
12	NAG	G	602	5	-	2/6/23/26	0/1/1/1
12	NAG	a	701	1	-	2/6/23/26	0/1/1/1
12	NAG	e	608	5	-	2/6/23/26	0/1/1/1
12	NAG	a	702	1	-	1/6/23/26	0/1/1/1
12	NAG	G	606	5	-	2/6/23/26	0/1/1/1
12	NAG	G	608	5	-	3/6/23/26	0/1/1/1
12	NAG	e	605	5	-	2/6/23/26	0/1/1/1
12	NAG	R	602	5	-	2/6/23/26	0/1/1/1
12	NAG	e	607	5	-	0/6/23/26	0/1/1/1
12	NAG	G	604	5	-	2/6/23/26	0/1/1/1
12	NAG	G	607	5	-	2/6/23/26	0/1/1/1
12	NAG	R	603	5	-	2/6/23/26	0/1/1/1
12	NAG	G	601	5	-	2/6/23/26	0/1/1/1
12	NAG	H	701	1	-	2/6/23/26	0/1/1/1
12	NAG	R	604	5	-	2/6/23/26	0/1/1/1
12	NAG	R	605	5	-	2/6/23/26	0/1/1/1
12	NAG	G	603	5	-	2/6/23/26	0/1/1/1
12	NAG	G	605	5	-	2/6/23/26	0/1/1/1
12	NAG	H	703	1	-	2/6/23/26	0/1/1/1
12	NAG	a	703	1	-	2/6/23/26	0/1/1/1
12	NAG	B	702	1	-	2/6/23/26	0/1/1/1
12	NAG	e	606	5	-	2/6/23/26	0/1/1/1
12	NAG	H	702	1	-	4/6/23/26	0/1/1/1
12	NAG	R	606	5	-	0/6/23/26	0/1/1/1
12	NAG	e	602	5	-	2/6/23/26	0/1/1/1
12	NAG	e	604	5	-	2/6/23/26	0/1/1/1
12	NAG	e	603	5	-	2/6/23/26	0/1/1/1
12	NAG	e	601	5	-	1/6/23/26	0/1/1/1
12	NAG	B	701	1	-	2/6/23/26	0/1/1/1
12	NAG	R	601	5	-	2/6/23/26	0/1/1/1
12	NAG	B	703	1	-	2/6/23/26	0/1/1/1

There are no bond length outliers.

All (2) bond angle outliers are listed below:

Mol	Chain	Res	Type	Atoms	Z	Observed(°)	Ideal(°)
12	H	702	NAG	C2-N2-C7	5.02	130.05	122.90

Continued on next page...

For Manuscript Review

Continued from previous page...

Mol	Chain	Res	Type	Atoms	Z	Observed(°)	Ideal(°)
12	H	702	NAG	C1-C2-N2	2.85	115.36	110.49

There are no chirality outliers.

All (59) torsion outliers are listed below:

Mol	Chain	Res	Type	Atoms
12	R	603	NAG	O5-C5-C6-O6
12	G	604	NAG	O5-C5-C6-O6
12	a	701	NAG	O5-C5-C6-O6
12	e	603	NAG	C4-C5-C6-O6
12	G	605	NAG	O5-C5-C6-O6
12	e	602	NAG	C4-C5-C6-O6
12	G	602	NAG	C4-C5-C6-O6
12	B	701	NAG	O5-C5-C6-O6
12	R	605	NAG	O5-C5-C6-O6
12	G	603	NAG	C4-C5-C6-O6
12	R	602	NAG	O5-C5-C6-O6
12	e	604	NAG	O5-C5-C6-O6
12	e	605	NAG	O5-C5-C6-O6
12	e	606	NAG	O5-C5-C6-O6
12	G	604	NAG	C4-C5-C6-O6
12	R	603	NAG	C4-C5-C6-O6
12	e	605	NAG	C4-C5-C6-O6
12	R	601	NAG	O5-C5-C6-O6
12	R	605	NAG	C4-C5-C6-O6
12	R	604	NAG	O5-C5-C6-O6
12	e	602	NAG	O5-C5-C6-O6
12	e	608	NAG	O5-C5-C6-O6
12	e	604	NAG	C4-C5-C6-O6
12	G	602	NAG	O5-C5-C6-O6
12	e	603	NAG	O5-C5-C6-O6
12	G	605	NAG	C4-C5-C6-O6
12	G	603	NAG	O5-C5-C6-O6
12	e	606	NAG	C4-C5-C6-O6
12	G	606	NAG	O5-C5-C6-O6
12	a	701	NAG	C4-C5-C6-O6
12	H	701	NAG	O5-C5-C6-O6
12	R	602	NAG	C4-C5-C6-O6
12	a	703	NAG	O5-C5-C6-O6
12	G	608	NAG	C8-C7-N2-C2
12	G	608	NAG	O7-C7-N2-C2
12	H	702	NAG	C8-C7-N2-C2

Continued on next page...

For Manuscript Review

Continued from previous page...

Mol	Chain	Res	Type	Atoms
12	H	702	NAG	O7-C7-N2-C2
12	B	701	NAG	C4-C5-C6-O6
12	H	701	NAG	C4-C5-C6-O6
12	R	604	NAG	C4-C5-C6-O6
12	G	606	NAG	C4-C5-C6-O6
12	G	601	NAG	O5-C5-C6-O6
12	G	608	NAG	O5-C5-C6-O6
12	a	703	NAG	C4-C5-C6-O6
12	R	601	NAG	C4-C5-C6-O6
12	e	608	NAG	C4-C5-C6-O6
12	H	702	NAG	C1-C2-N2-C7
12	B	702	NAG	O5-C5-C6-O6
12	e	601	NAG	O5-C5-C6-O6
12	H	702	NAG	O5-C5-C6-O6
12	G	607	NAG	C4-C5-C6-O6
12	a	702	NAG	O5-C5-C6-O6
12	G	607	NAG	O5-C5-C6-O6
12	B	703	NAG	C4-C5-C6-O6
12	B	703	NAG	O5-C5-C6-O6
12	H	703	NAG	C4-C5-C6-O6
12	G	601	NAG	C4-C5-C6-O6
12	H	703	NAG	O5-C5-C6-O6
12	B	702	NAG	C3-C2-N2-C7

There are no ring outliers.

No monomer is involved in short contacts.

5.7 Other polymers ⓘ

There are no such residues in this entry.

5.8 Polymer linkage issues ⓘ

There are no chain breaks in this entry.

For Manuscript Review

6 Fit of model and data (i)

6.1 Protein, DNA and RNA chains (i)

Unable to reproduce the depositors R factor - this section is therefore empty.

6.2 Non-standard residues in protein, DNA, RNA chains (i)

Unable to reproduce the depositors R factor - this section is therefore empty.

6.3 Carbohydrates (i)

Unable to reproduce the depositors R factor - this section is therefore empty.

The following is a graphical depiction of the model fit to experimental electron density for oligosaccharide. Each fit is shown from different orientation to approximate a three-dimensional view.

Electron density around Chain A:

2mF$_o$-DF$_c$ (at 0.7 rmsd) in gray
mF$_o$-DF$_c$ (at 3 rmsd) in purple (negative)
and green (positive)

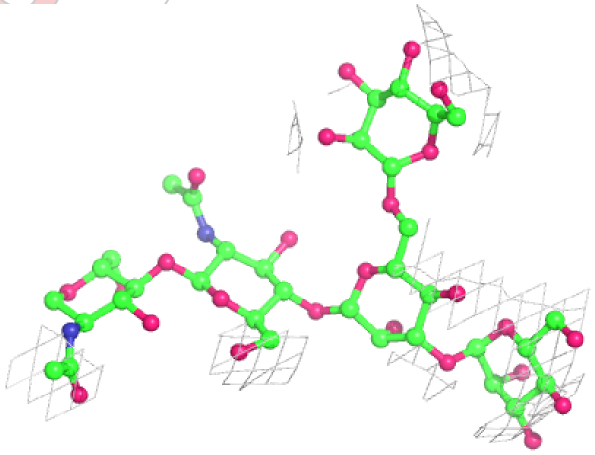

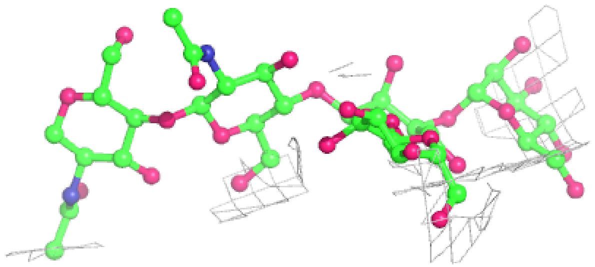

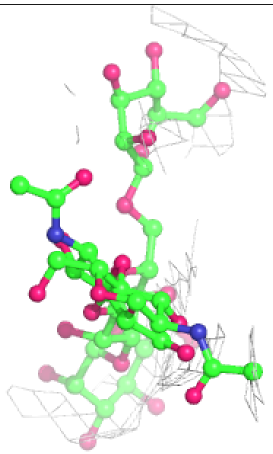

For Manuscript Review

Electron density around Chain F:

$2mF_o$-DF_c (at 0.7 rmsd) in gray
mF_o-DF_c (at 3 rmsd) in purple (negative)
and green (positive)

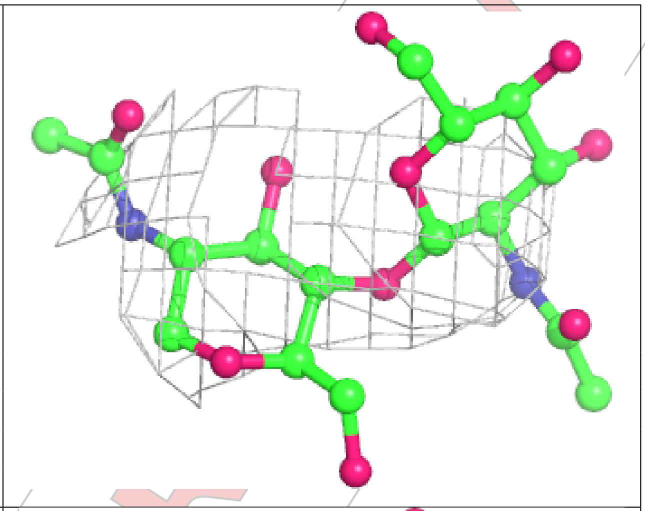

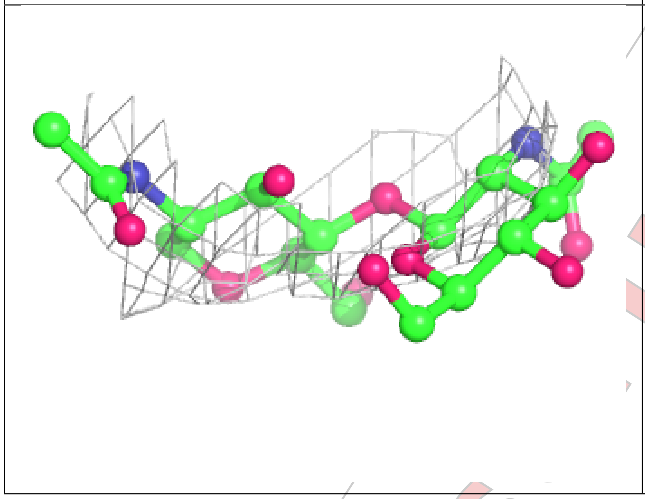

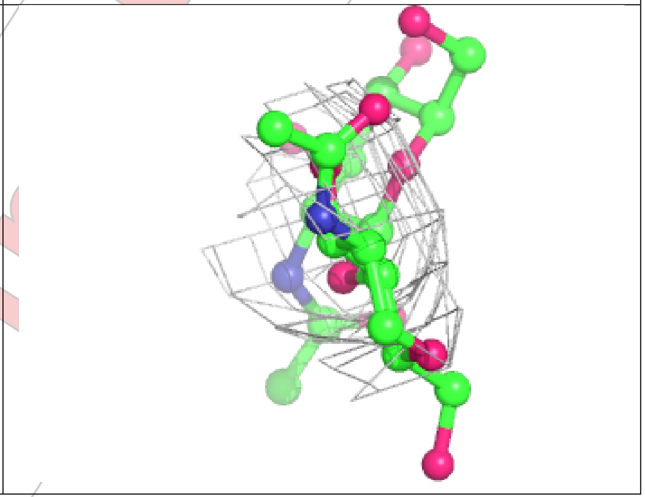

For Manuscript Review

Electron density around Chain K:

$2mF_o$-DF_c (at 0.7 rmsd) in gray
mF_o-DF_c (at 3 rmsd) in purple (negative)
and green (positive)

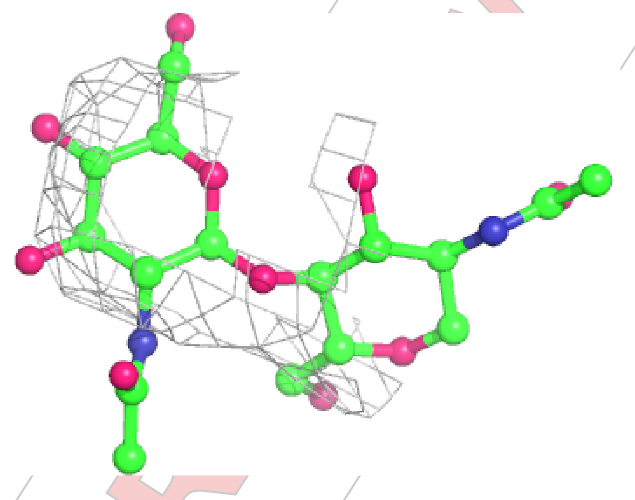

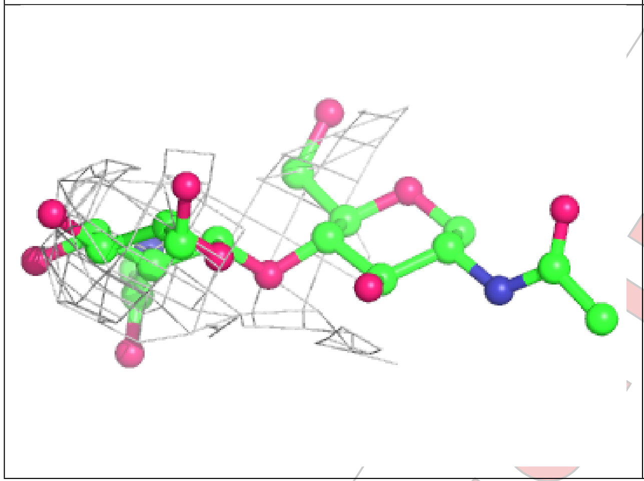

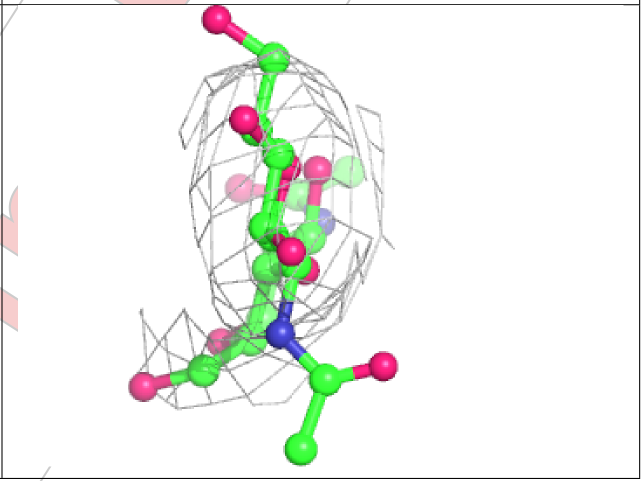

For Manuscript Review

Electron density around Chain M:

$2mF_o$-DF_c (at 0.7 rmsd) in gray
mF_o-DF_c (at 3 rmsd) in purple (negative)
and green (positive)

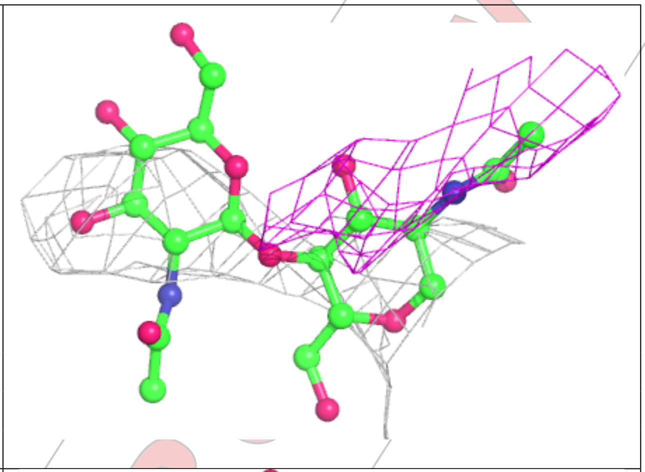

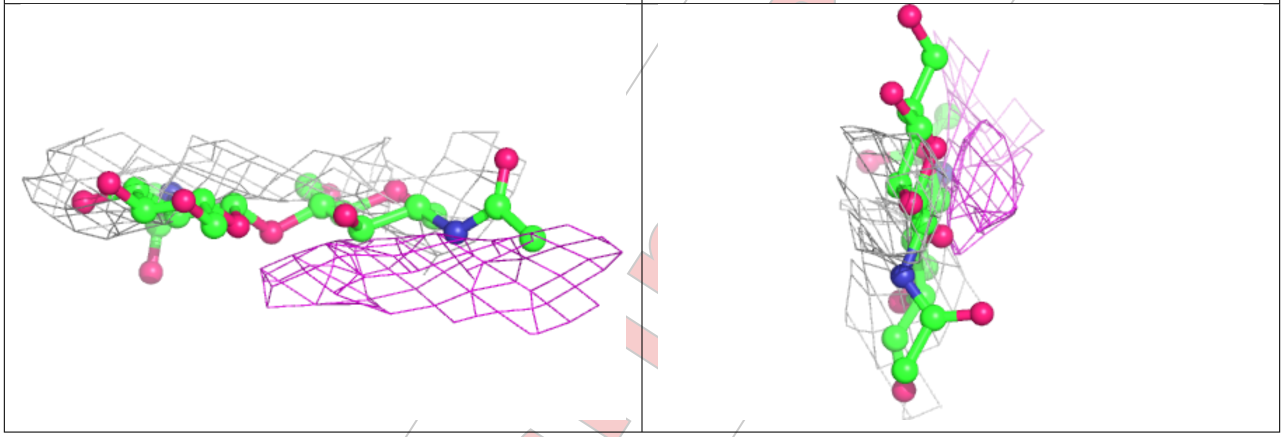

Electron density around Chain T:

$2mF_o$-DF_c (at 0.7 rmsd) in gray
mF_o-DF_c (at 3 rmsd) in purple (negative)
and green (positive)

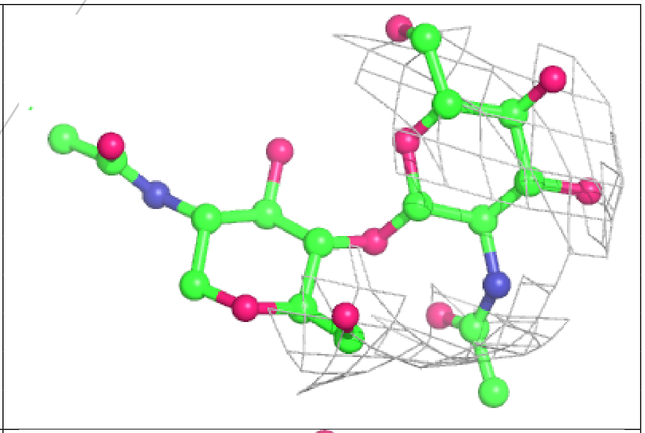

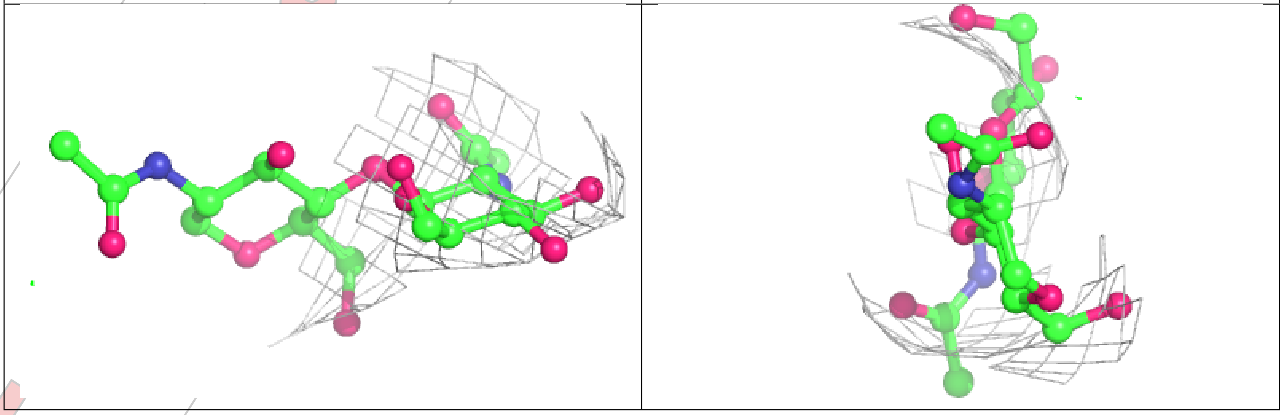

Electron density around Chain U:

$2mF_o$-DF_c (at 0.7 rmsd) in gray
mF_o-DF_c (at 3 rmsd) in purple (negative) and green (positive)

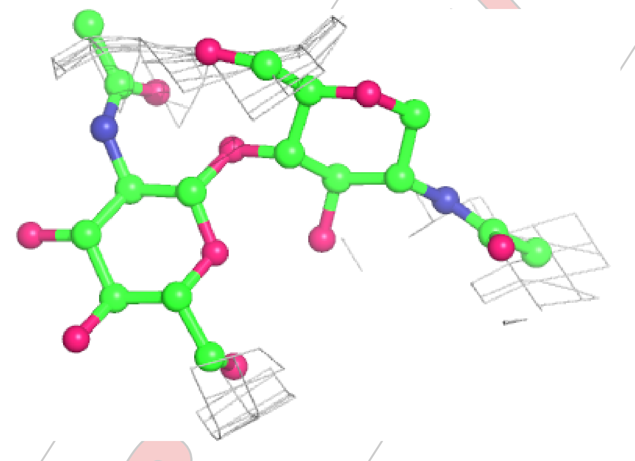

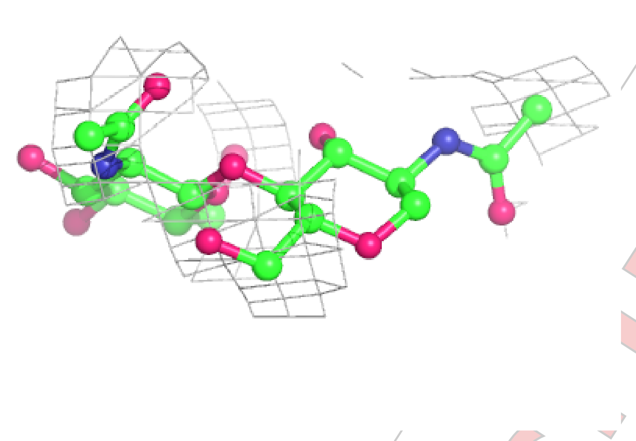

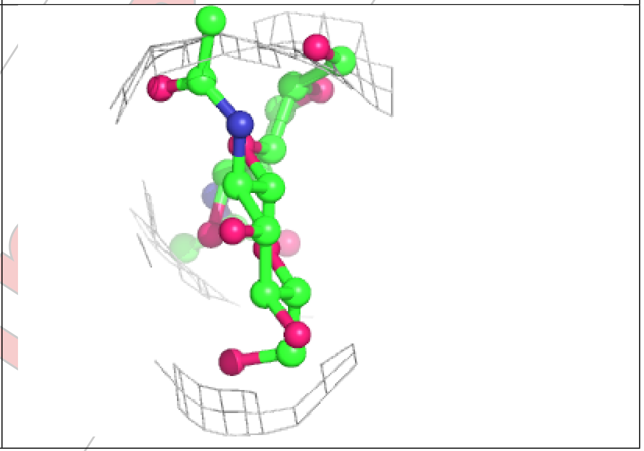

For Manuscript Review

Electron density around Chain W:

$2mF_o$-DF_c (at 0.7 rmsd) in gray
mF_o-DF_c (at 3 rmsd) in purple (negative)
and green (positive)

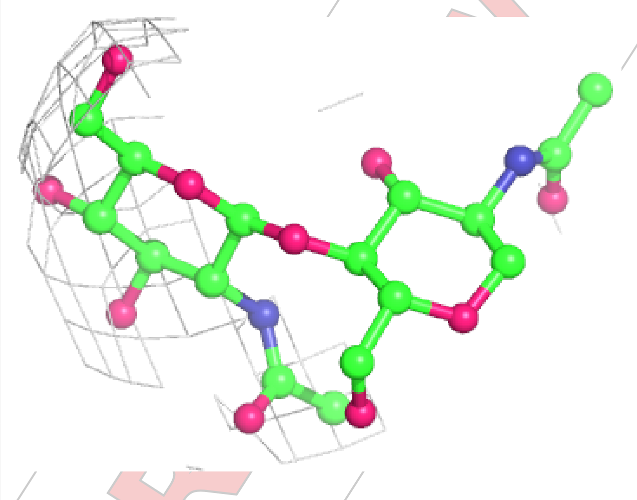

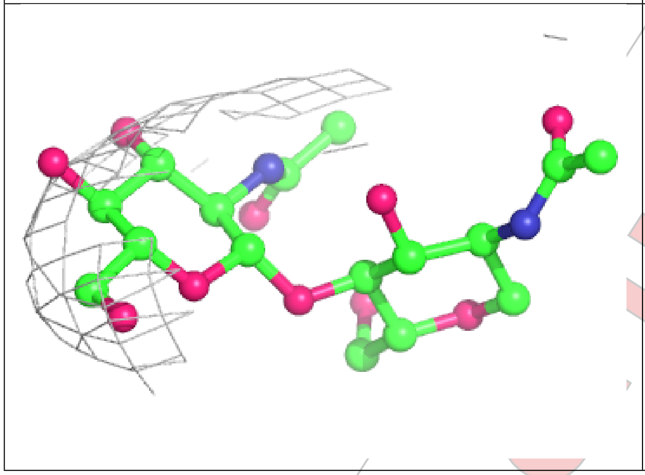

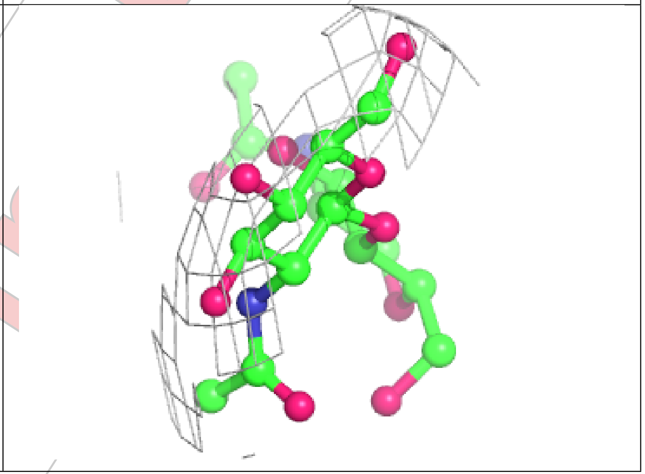

For Manuscript Review

Electron density around Chain X:

2mF_o-DF_c (at 0.7 rmsd) in gray
mF_o-DF_c (at 3 rmsd) in purple (negative)
and green (positive)

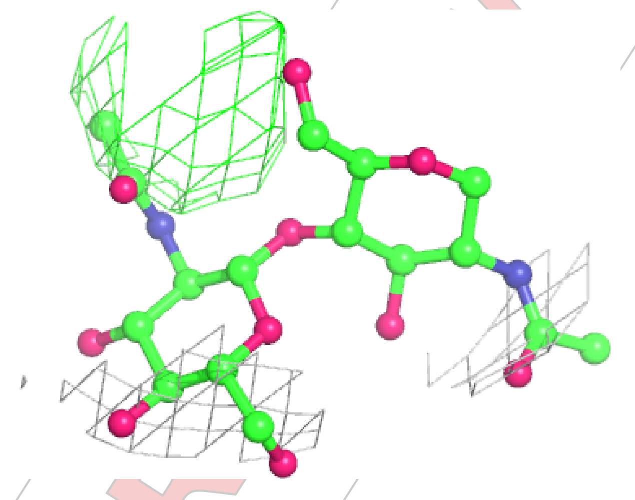

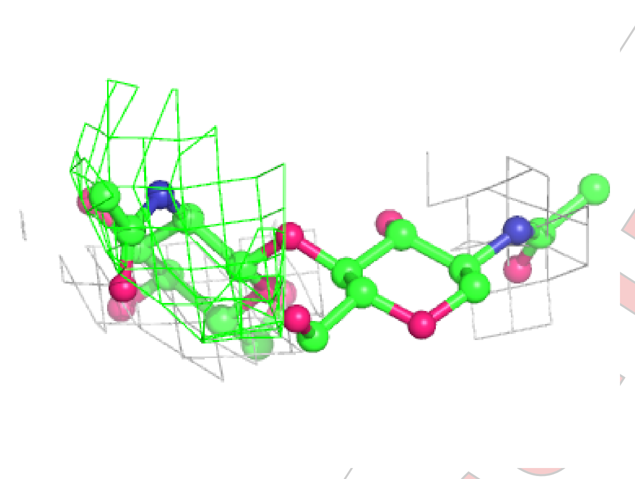

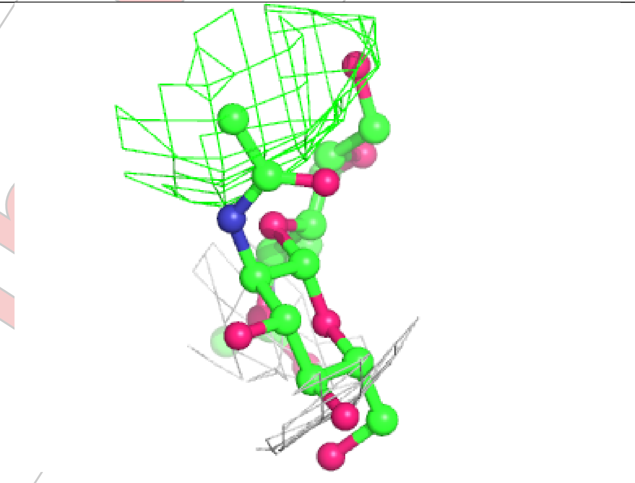

For Manuscript Review

Electron density around Chain g:

2mF$_o$-DF$_c$ (at 0.7 rmsd) in gray
mF$_o$-DF$_c$ (at 3 rmsd) in purple (negative)
and green (positive)

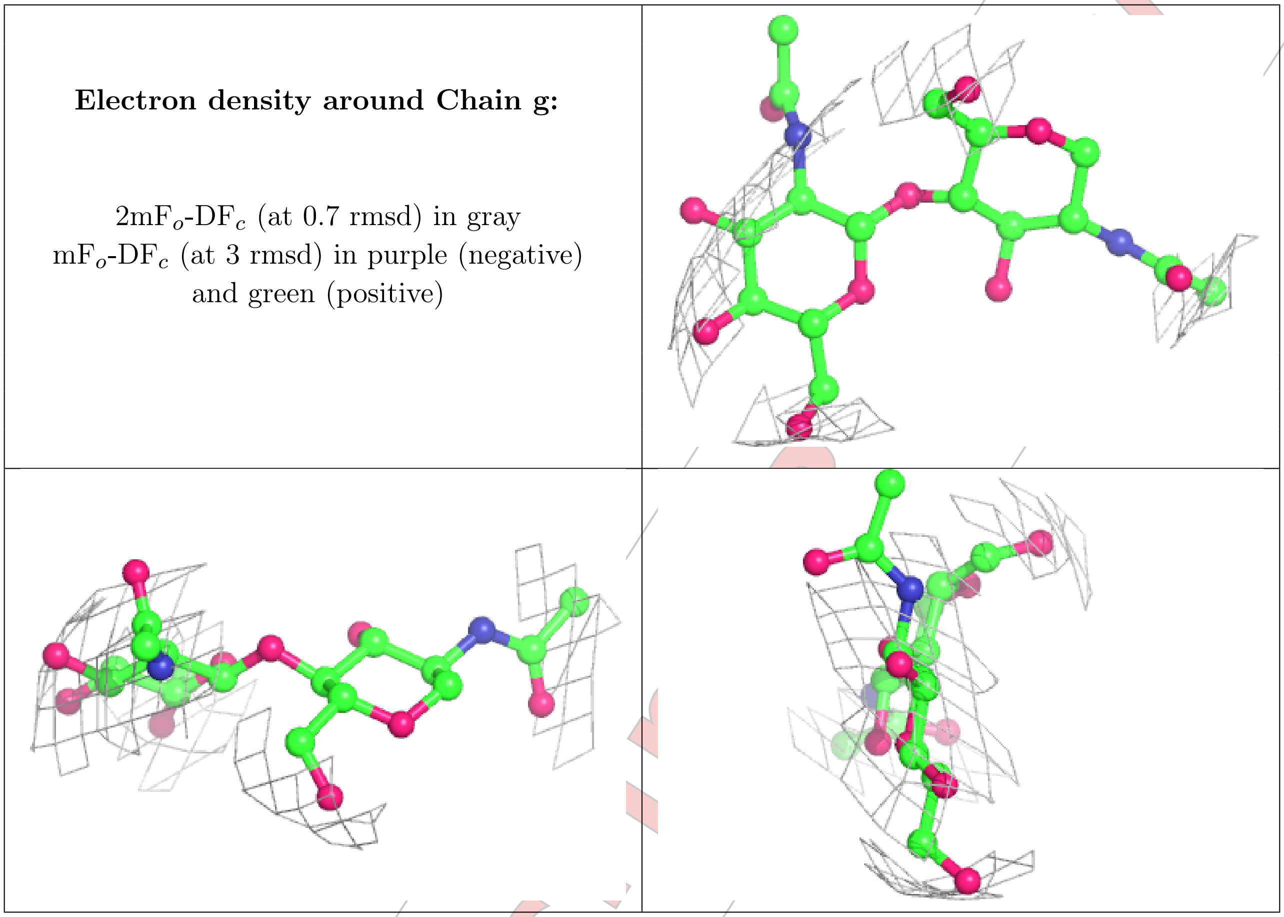

For Manuscript Review

Electron density around Chain h:

$2mF_o$-DF_c (at 0.7 rmsd) in gray
mF_o-DF_c (at 3 rmsd) in purple (negative)
and green (positive)

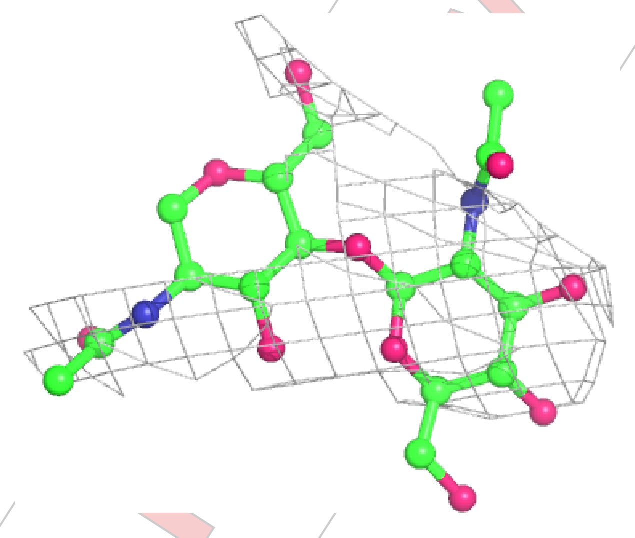

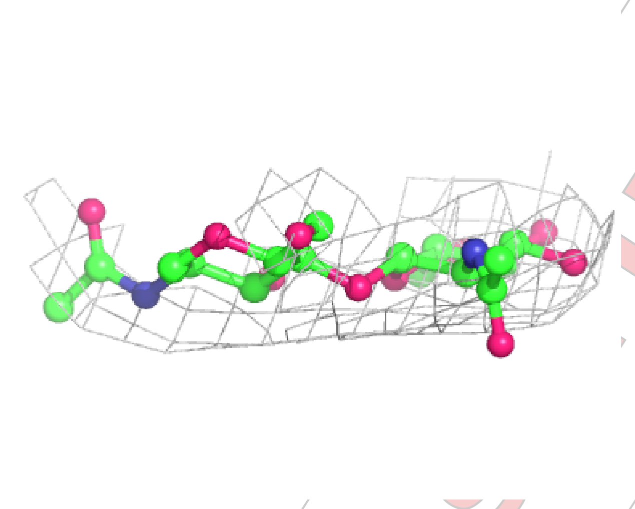

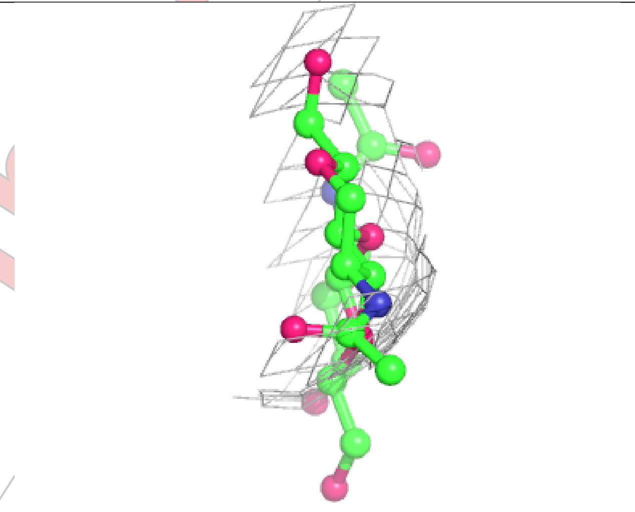

For Manuscript Review

Electron density around Chain j:

$2mF_o$-DF_c (at 0.7 rmsd) in gray
mF_o-DF_c (at 3 rmsd) in purple (negative)
and green (positive)

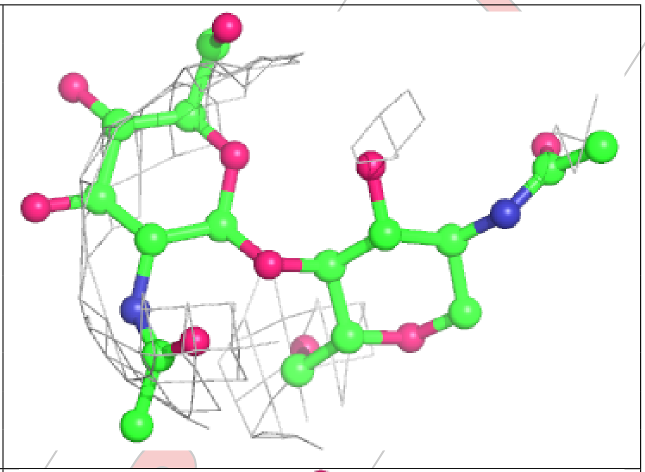

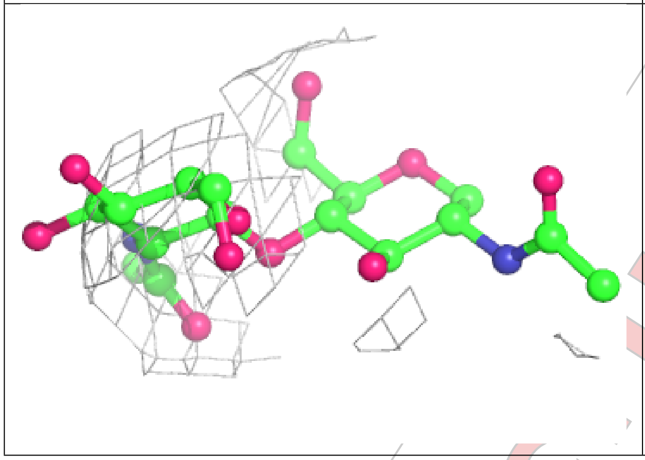

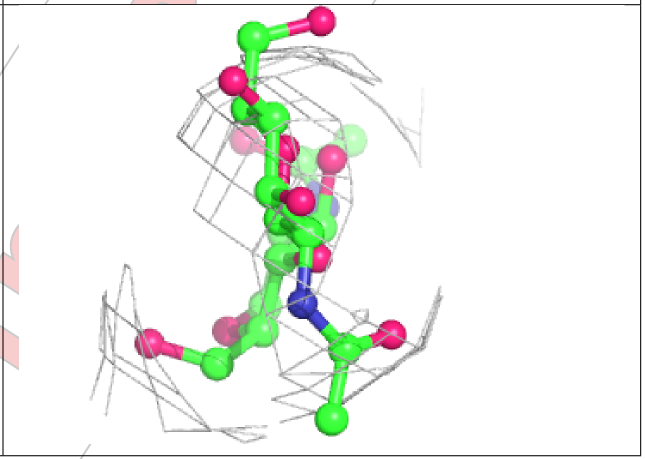

For Manuscript Review

Electron density around Chain l:

2mF$_o$-DF$_c$ (at 0.7 rmsd) in gray
mF$_o$-DF$_c$ (at 3 rmsd) in purple (negative)
and green (positive)

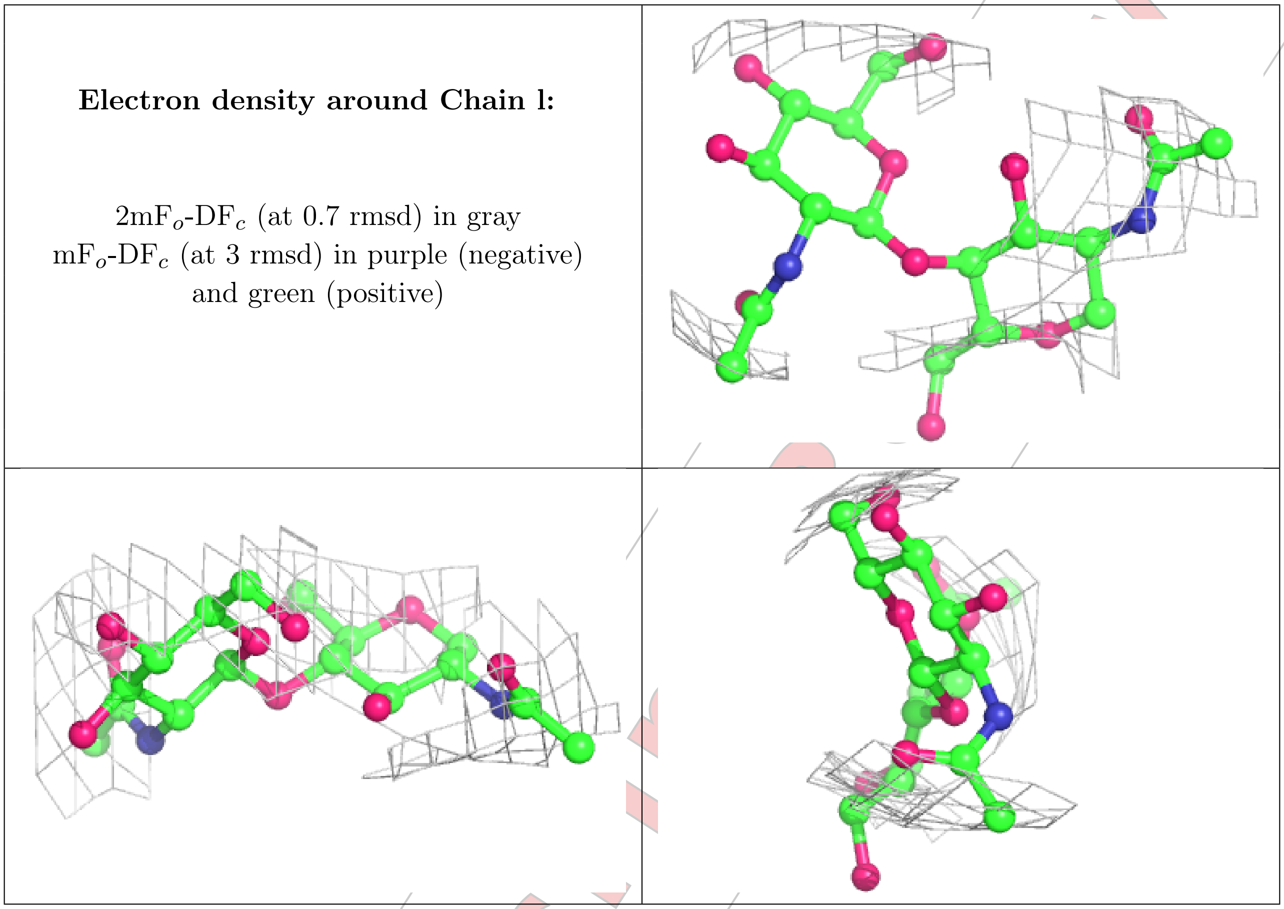

For Manuscript Review

Electron density around Chain o:

2mF$_o$-DF$_c$ (at 0.7 rmsd) in gray
mF$_o$-DF$_c$ (at 3 rmsd) in purple (negative)
and green (positive)

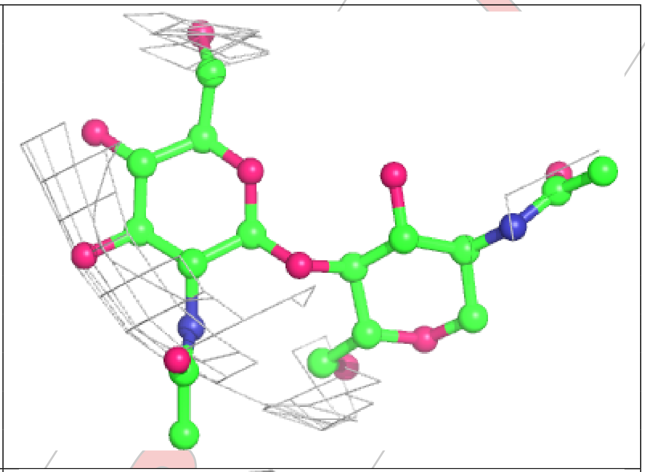

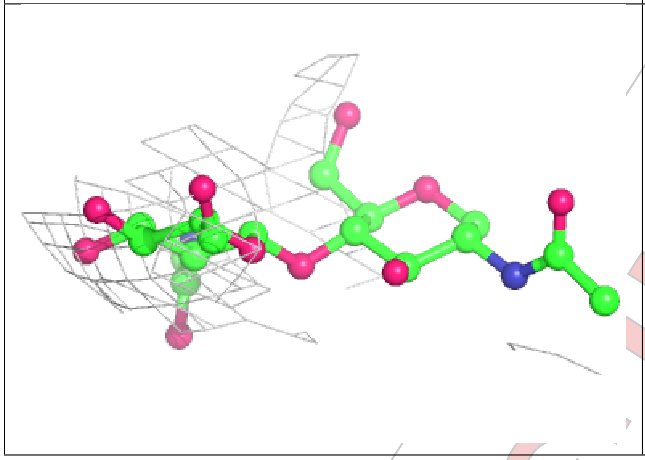

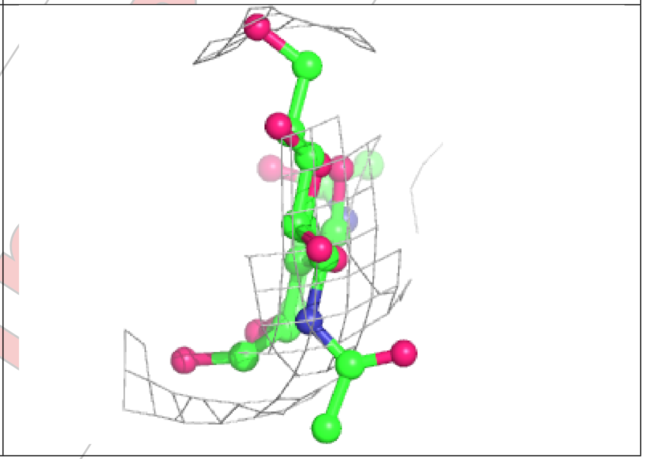

For Manuscript Review

Electron density around Chain p:

$2mF_o$-DF_c (at 0.7 rmsd) in gray
mF_o-DF_c (at 3 rmsd) in purple (negative)
and green (positive)

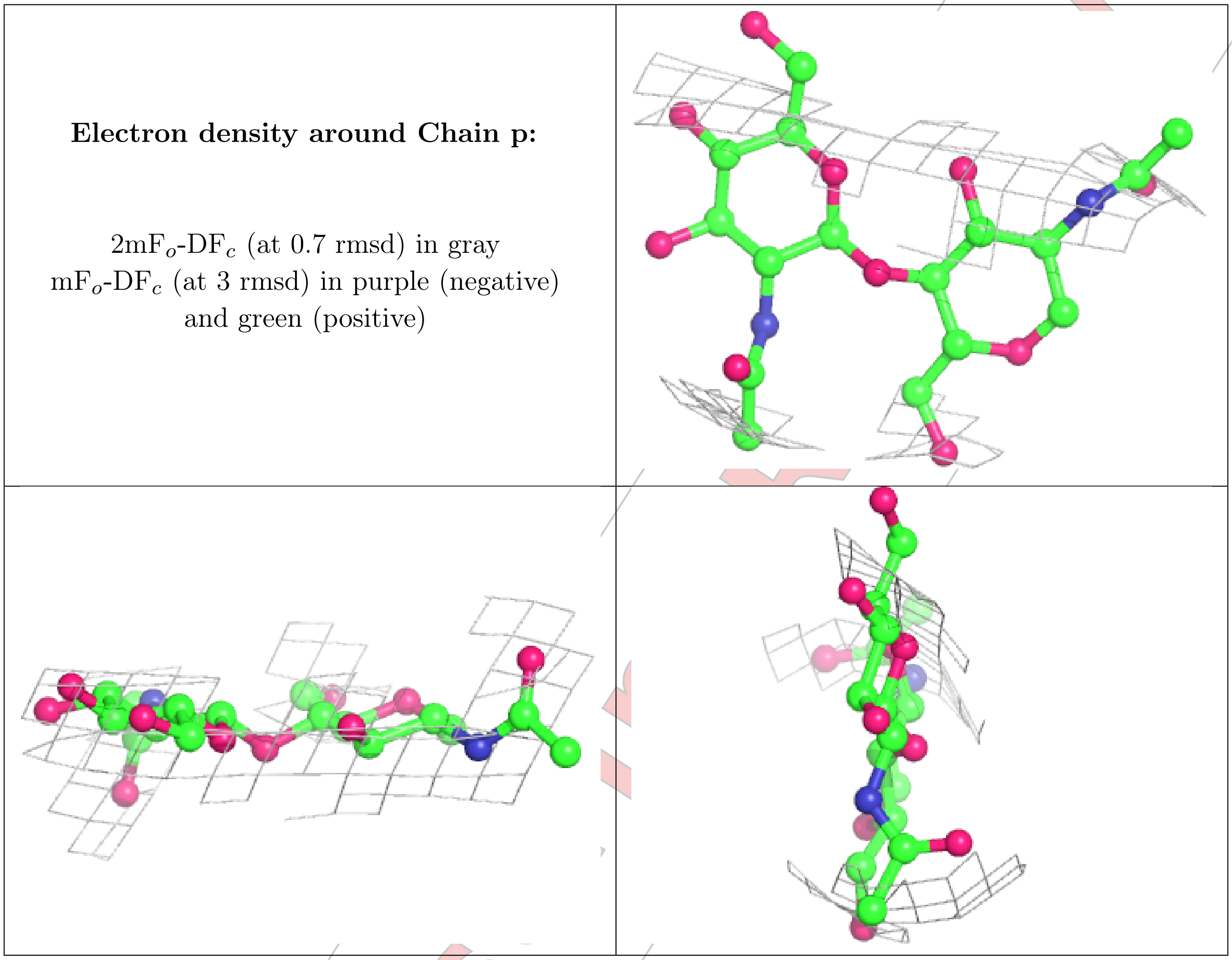

For Manuscript Review

Electron density around Chain r:

2mF$_o$-DF$_c$ (at 0.7 rmsd) in gray
mF$_o$-DF$_c$ (at 3 rmsd) in purple (negative)
and green (positive)

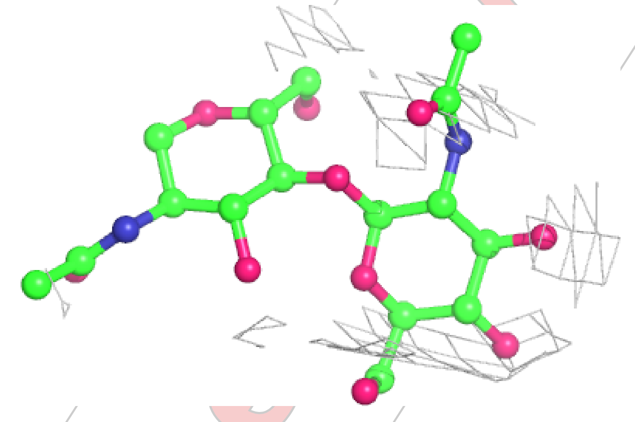

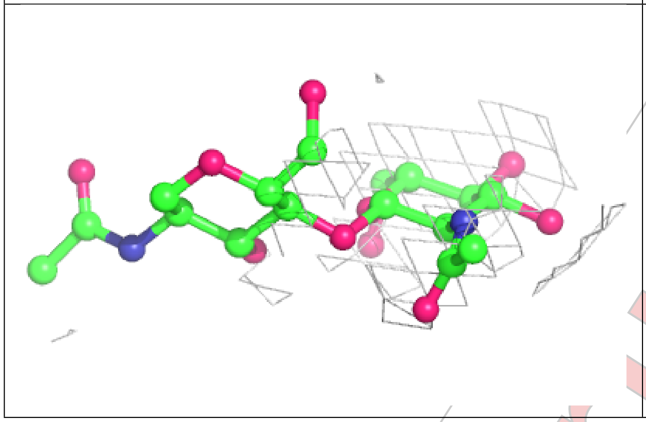

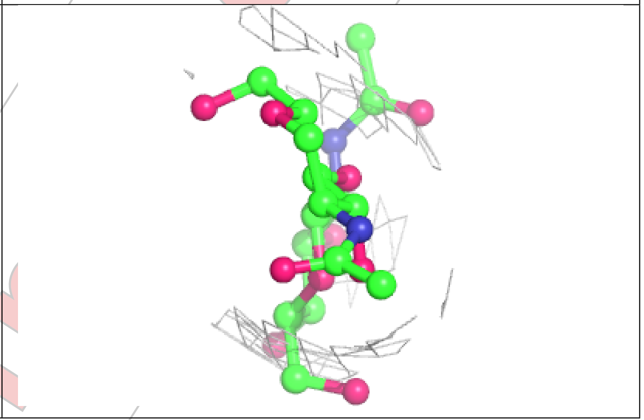

Electron density around Chain I:

2mF$_o$-DF$_c$ (at 0.7 rmsd) in gray
mF$_o$-DF$_c$ (at 3 rmsd) in purple (negative)
and green (positive)

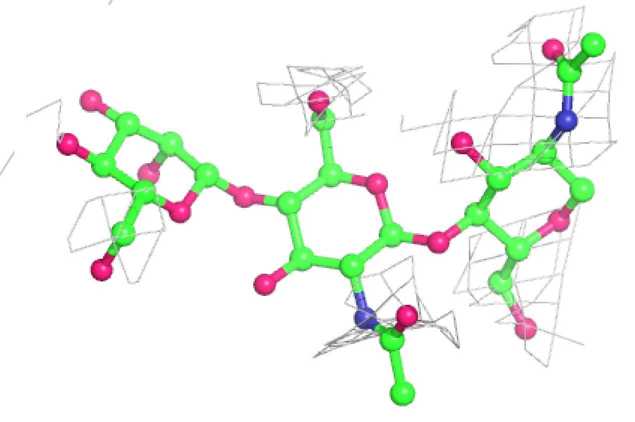

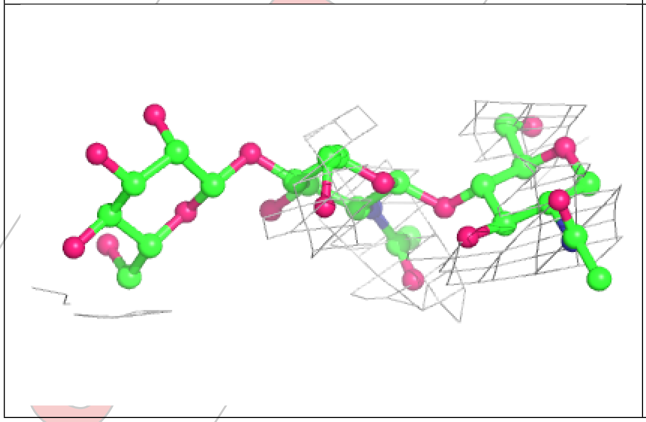

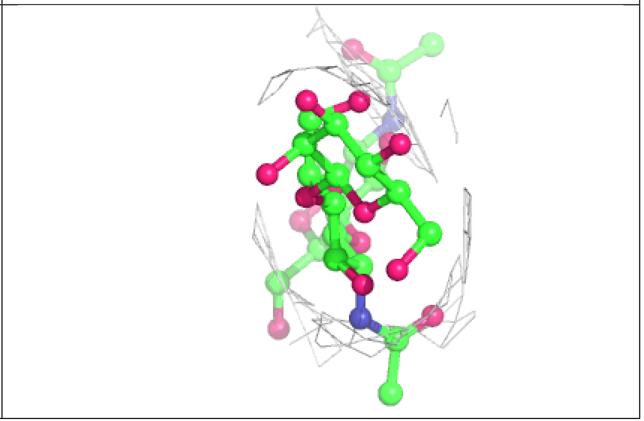

For Manuscript Review

Electron density around Chain J:

2mF$_o$-DF$_c$ (at 0.7 rmsd) in gray
mF$_o$-DF$_c$ (at 3 rmsd) in purple (negative)
and green (positive)

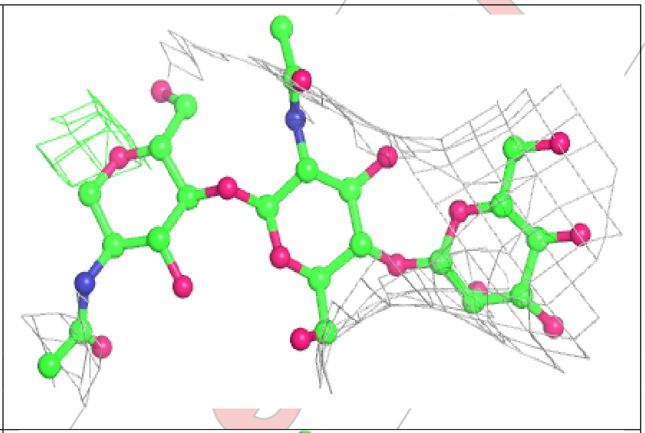

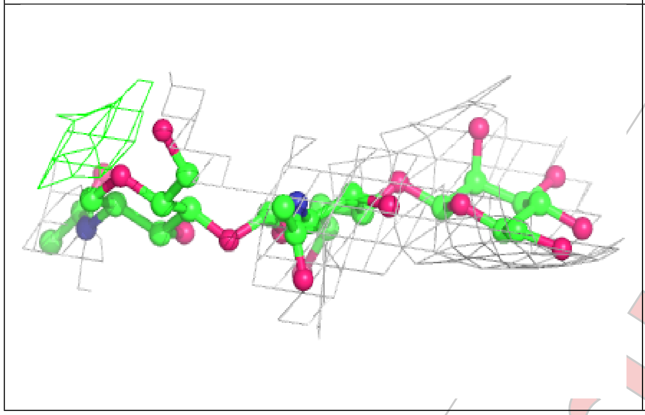

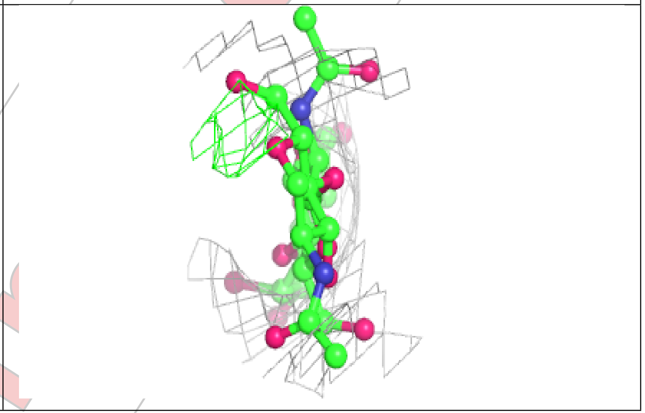

For Manuscript Review

Electron density around Chain Y:

$2mF_o$-DF_c (at 0.7 rmsd) in gray
mF_o-DF_c (at 3 rmsd) in purple (negative)
and green (positive)

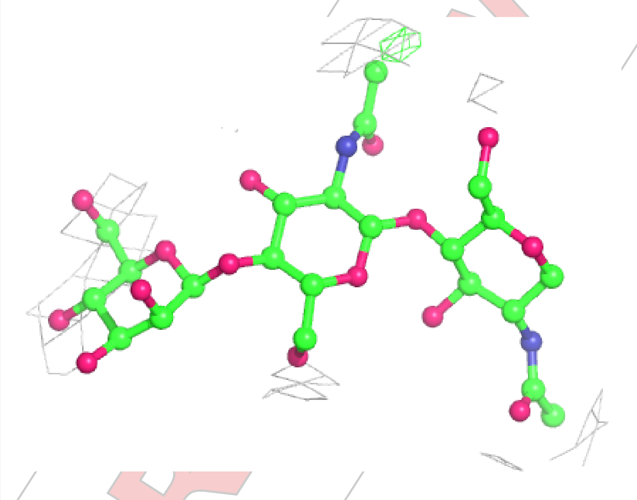

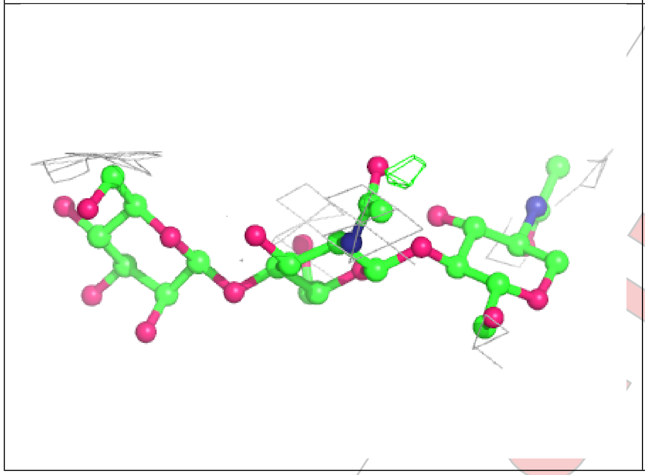

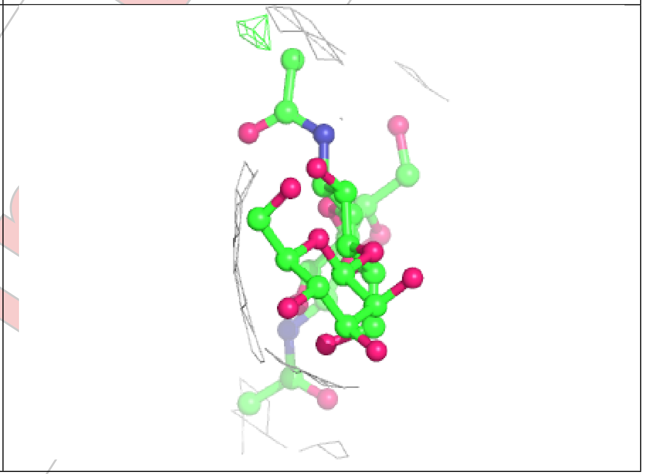

For Manuscript Review

Electron density around Chain Z:

$2mF_o$-DF_c (at 0.7 rmsd) in gray
mF_o-DF_c (at 3 rmsd) in purple (negative)
and green (positive)

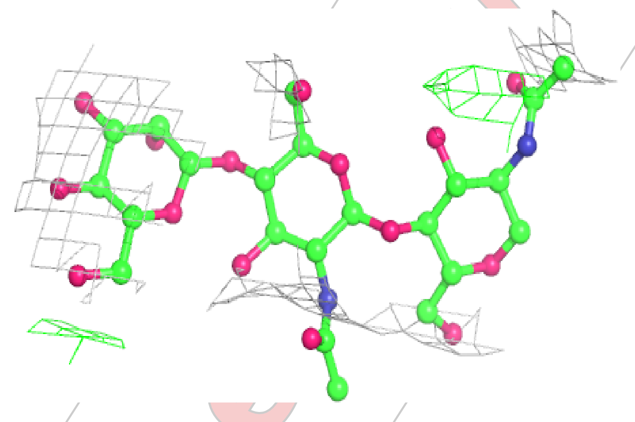

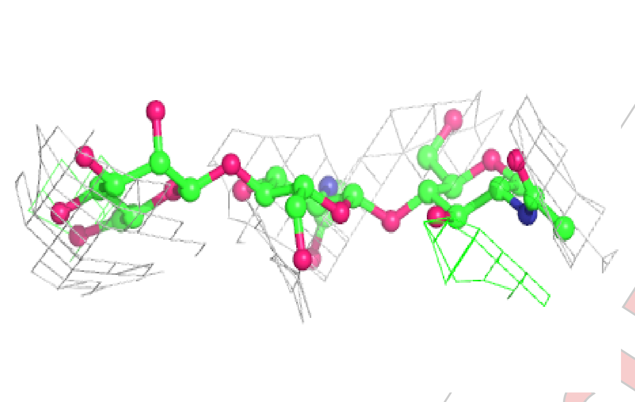

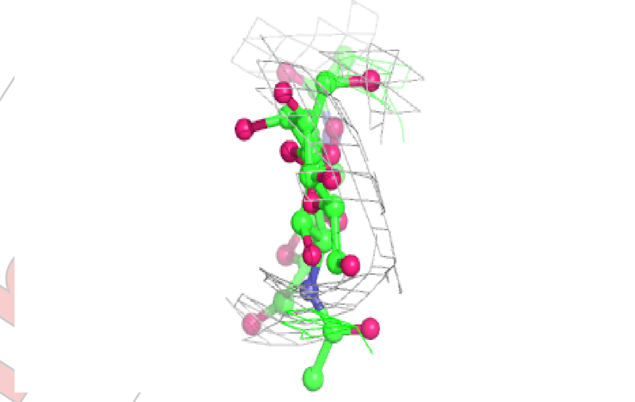

Electron density around Chain m:

$2mF_o$-DF_c (at 0.7 rmsd) in gray
mF_o-DF_c (at 3 rmsd) in purple (negative)
and green (positive)

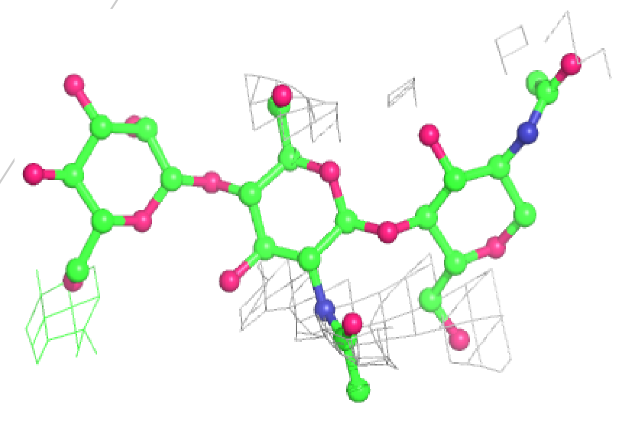

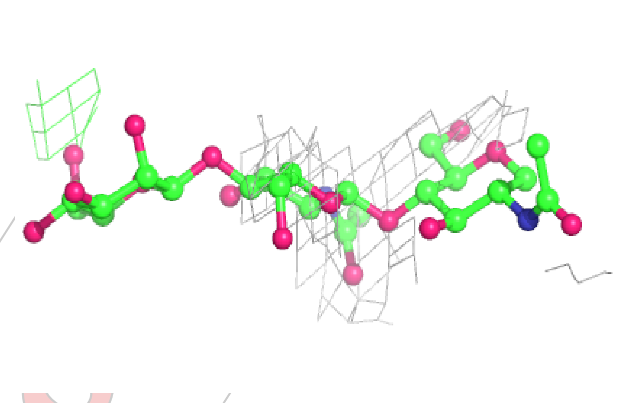

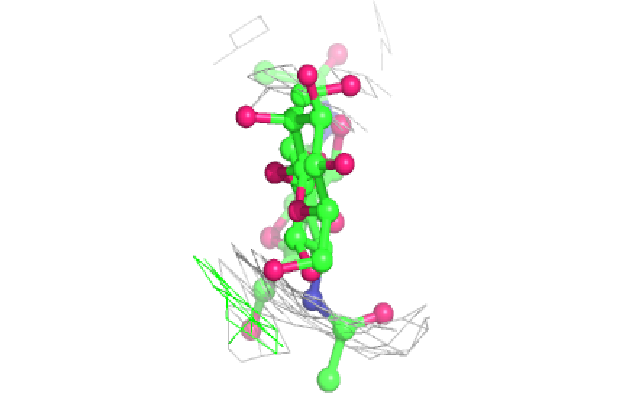

Electron density around Chain n:

$2mF_o$-DF_c (at 0.7 rmsd) in gray
mF_o-DF_c (at 3 rmsd) in purple (negative)
and green (positive)

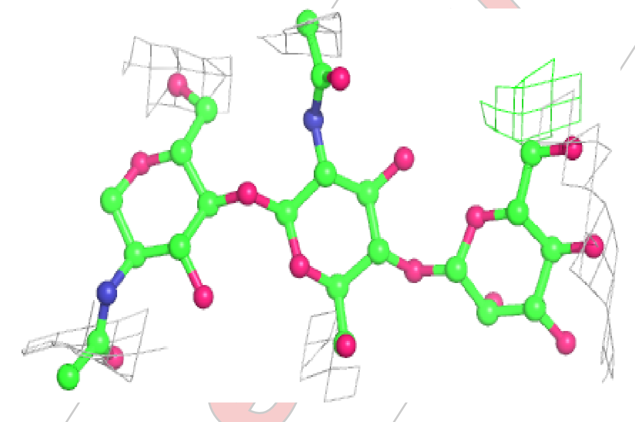

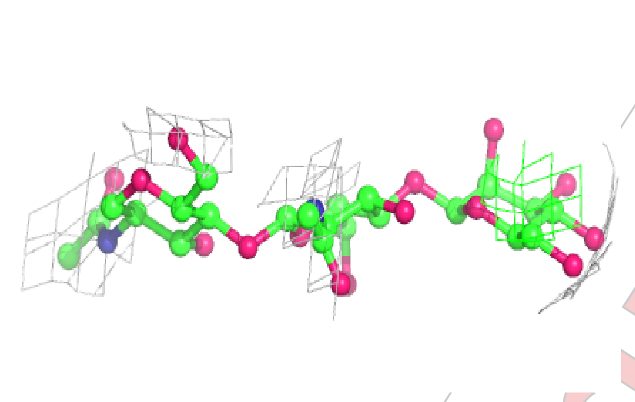

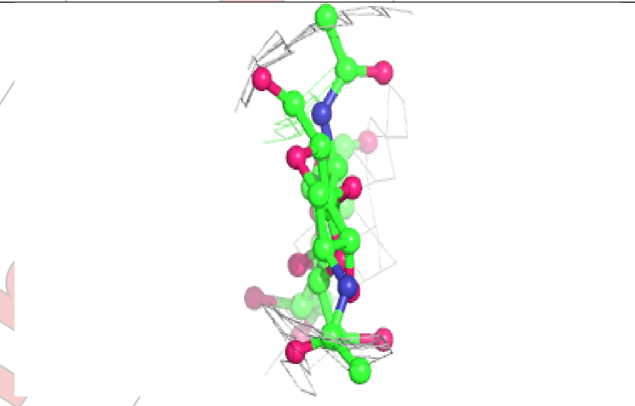

Electron density around Chain N:

$2mF_o$-DF_c (at 0.7 rmsd) in gray
mF_o-DF_c (at 3 rmsd) in purple (negative)
and green (positive)

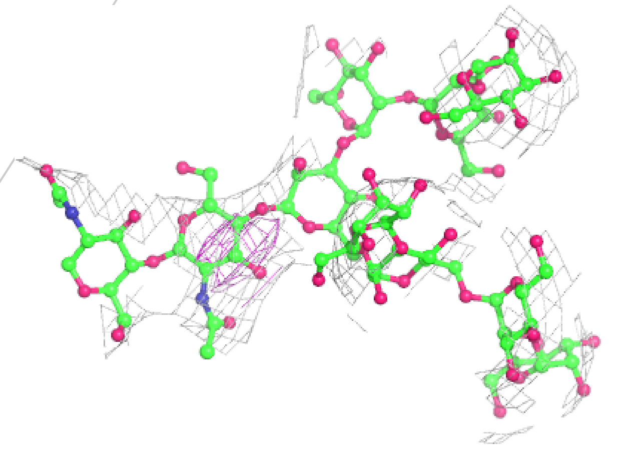

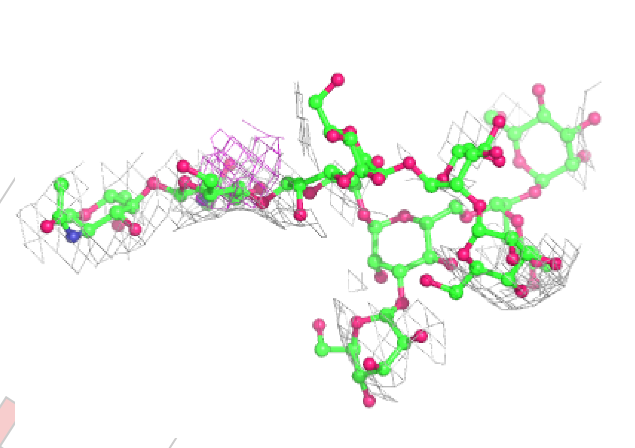

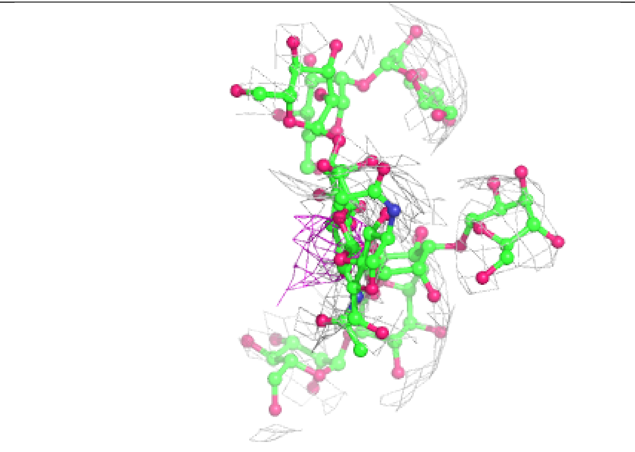

Electron density around Chain i:

2mF$_o$-DF$_c$ (at 0.7 rmsd) in gray
mF$_o$-DF$_c$ (at 3 rmsd) in purple (negative)
and green (positive)

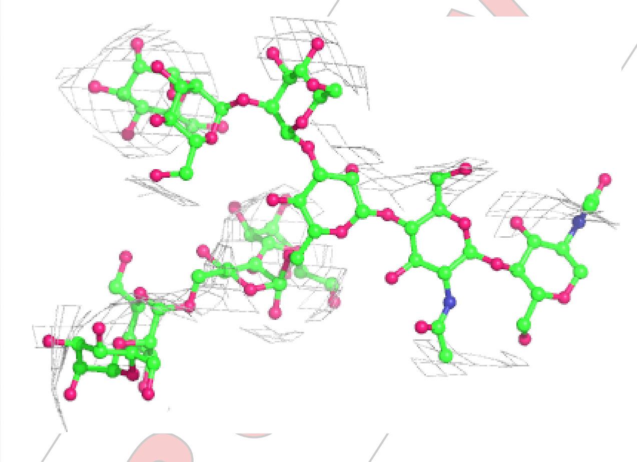

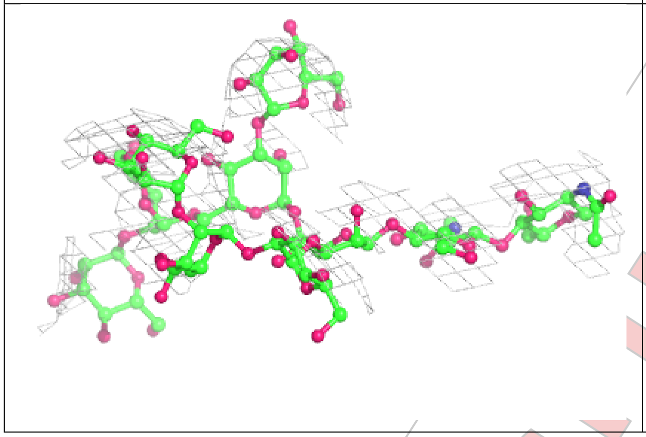

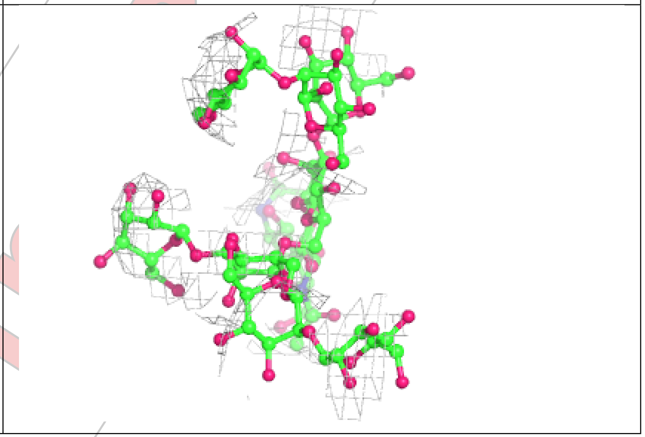

Electron density around Chain q:

2mF$_o$-DF$_c$ (at 0.7 rmsd) in gray
mF$_o$-DF$_c$ (at 3 rmsd) in purple (negative)
and green (positive)

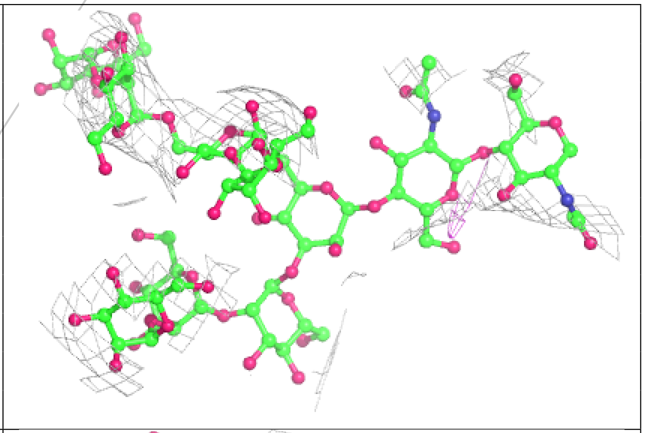

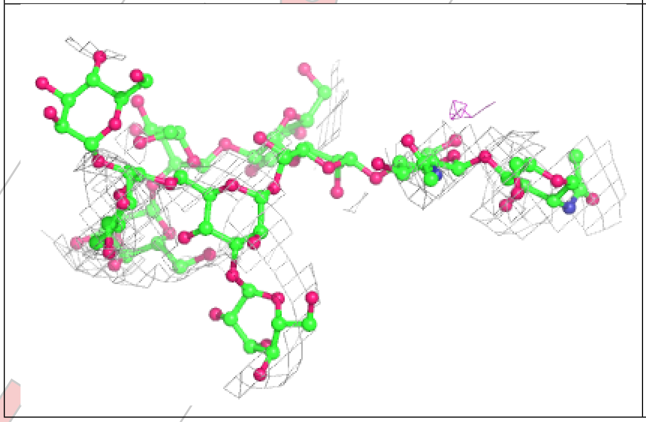

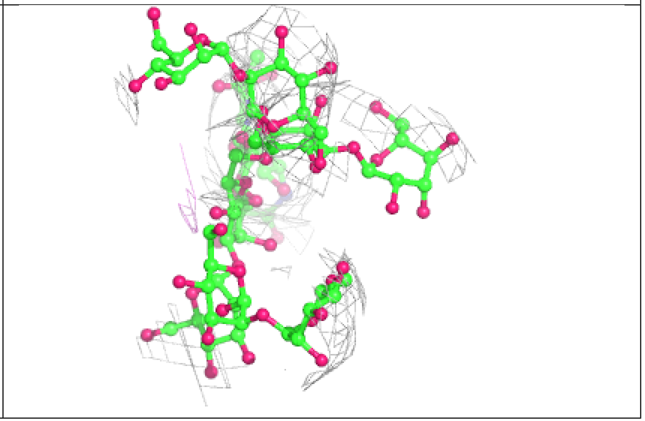

Electron density around Chain V:

$2mF_o$-DF_c (at 0.7 rmsd) in gray
mF_o-DF_c (at 3 rmsd) in purple (negative)
and green (positive)

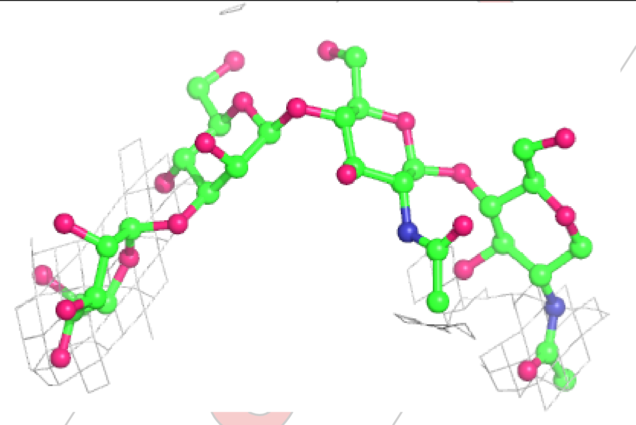

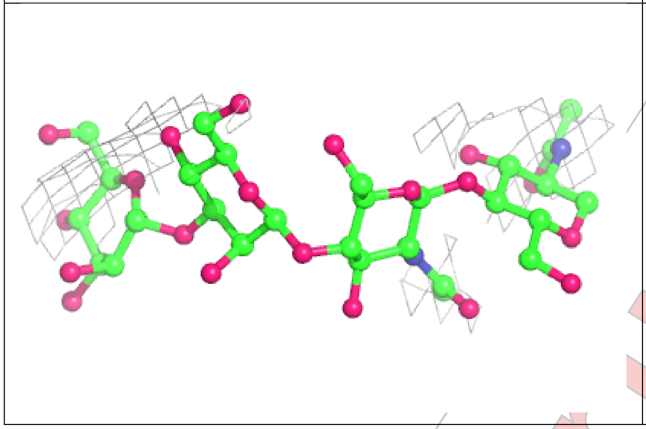

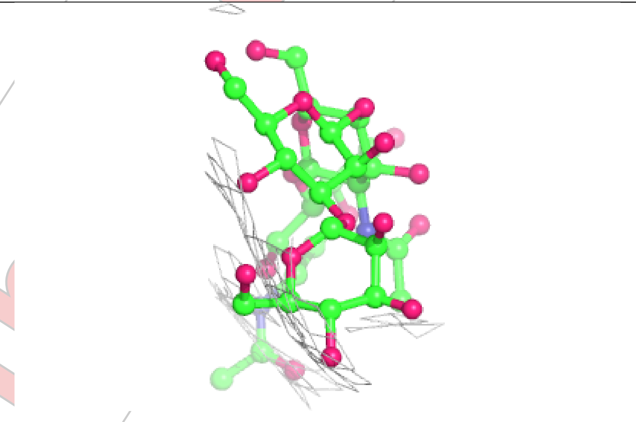

Electron density around Chain k:

$2mF_o$-DF_c (at 0.7 rmsd) in gray
mF_o-DF_c (at 3 rmsd) in purple (negative)
and green (positive)

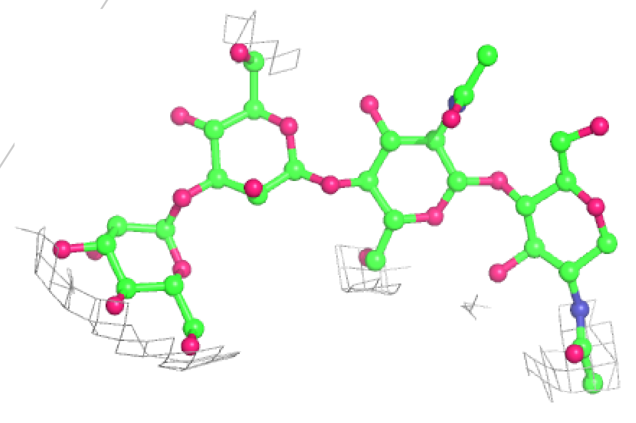

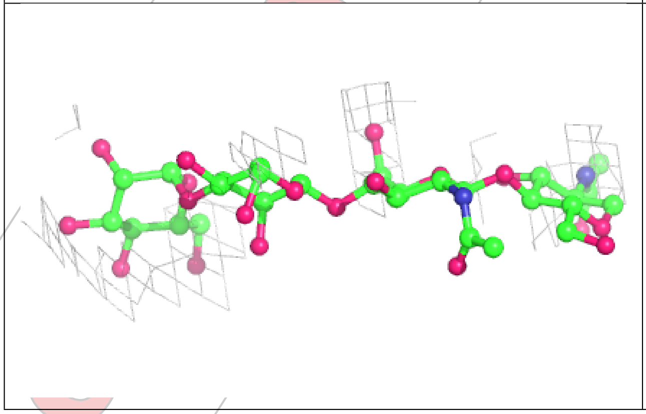

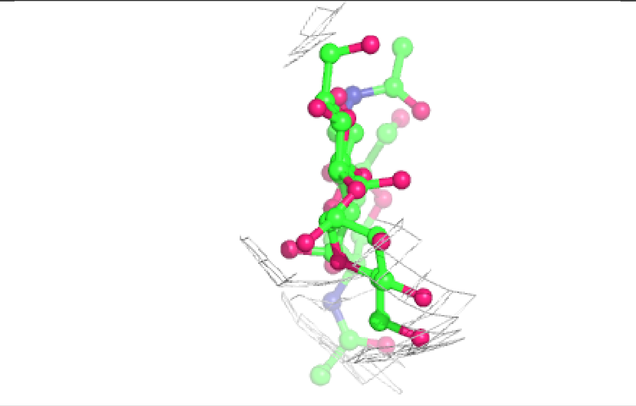

For Manuscript Review

6.4 Ligands (i)

Unable to reproduce the depositors R factor - this section is therefore empty.

6.5 Other polymers (i)

Unable to reproduce the depositors R factor - this section is therefore empty.

For Manuscript Review
